# BID expression determines the apoptotic fate of cancer cells after abrogation of the spindle assembly checkpoint by AURKB or TTK inhibitors

**DOI:** 10.1186/s12943-023-01815-w

**Published:** 2023-07-13

**Authors:** Jordi Bertran-Alamillo, Ana Giménez-Capitán, Ruth Román, Sara Talbot, Rebecca Whiteley, Nicolas Floc’h, Elizabeth Martínez-Pérez, Matthew J. Martin, Paul D. Smith, Ivana Sullivan, Mikkel G. Terp, Jamal Saeh, Cristina Marino-Buslje, Giulia Fabbri, Grace Guo, Man Xu, Cristian Tornador, Andrés Aguilar-Hernández, Noemí Reguart, Henrik J. Ditzel, Alejandro Martínez-Bueno, Núria Nabau-Moretó, Amaya Gascó, Rafael Rosell, J. Elizabeth Pease, Urszula M. Polanska, Jon Travers, Jelena Urosevic, Miguel A. Molina-Vila

**Affiliations:** 1 Laboratory of Oncology, Pangaea Oncology, Quiron Dexeus University Hospital, C/ Sabino Arana 5-19, 08913 Barcelona, Spain; 2 Bioscience, Research and Early Development, Oncology R&D, AstraZeneca, Cambridge, CB21 6GH UK; 3 Bioinformatics Unit, Fundación Instituto Leloir, Buenos Aires, C1405BWE Argentina; 4 Servicio de Oncología Médica, Hospital de la Santa Creu i Sant Pau, Barcelona, 08025 Spain; 5 Instituto Oncológico Dr. Rosell, Hospital Universitario Dexeus, Barcelona, 08028 Spain; 6 Department of Cancer and Inflammation Research, Institute of Molecular Medicine, University of Southern Denmark, Odense C, 5000 Denmark; 7 Bioscience, Research and Early Development, Oncology R&D, AstraZeneca, Waltham, MA 02451 USA; 8 Translational Medicine, Research and Early Development, Oncology R&D, AstraZeneca, Waltham, MA 02451 USA; 9 Teresa Moretó Foundation and Wholegenix SL, Barcelona, 08021 Spain; 10 Thoracic Oncology Unit, Department of Medical Oncology, Hospital Clínic, Barcelona, 08036 Spain; 11 Department of Oncology, Odense University Hospital, Odense, 5000 Denmark; 12 Bioscience, Research and Early Development, Oncology R&D, AstraZeneca, Gaithersburg, MD 20878 USA; 13 Germans Trias i Pujol Research Institute (IGTP), Badalona, 08916 Spain

**Keywords:** Spindle assembly checkpoint (SAC), Abrogation, BID, Biomarker, Tumor, CASP-2, AURKB inhibitor, TTK inhibitor, Cell cycle

## Abstract

**Background:**

Drugs targeting the spindle assembly checkpoint (SAC), such as inhibitors of Aurora kinase B (AURKB) and dual specific protein kinase TTK, are in different stages of clinical development. However, cell response to SAC abrogation is poorly understood and there are no markers for patient selection.

**Methods:**

A panel of 53 tumor cell lines of different origins was used. The effects of drugs were analyzed by MTT and flow cytometry. Copy number status was determined by FISH and Q-PCR; mRNA expression by nCounter and RT-Q-PCR and protein expression by Western blotting. CRISPR-Cas9 technology was used for gene knock-out (KO) and a doxycycline-inducible pTRIPZ vector for ectopic expression. Finally, in vivo experiments were performed by implanting cultured cells or fragments of tumors into immunodeficient mice.

**Results:**

Tumor cells and patient-derived xenografts (PDXs) sensitive to AURKB and TTK inhibitors consistently showed high expression levels of BH3-interacting domain death agonist (BID), while cell lines and PDXs with low BID were uniformly resistant. Gene silencing rendered BID-overexpressing cells insensitive to SAC abrogation while ectopic BID expression in BID-low cells significantly increased sensitivity. SAC abrogation induced activation of CASP-2, leading to cleavage of CASP-3 and extensive cell death only in presence of high levels of BID. Finally, a prevalence study revealed high *BID* mRNA in 6% of human solid tumors.

**Conclusions:**

The fate of tumor cells after SAC abrogation is driven by an AURKB/ CASP-2 signaling mechanism, regulated by BID levels. Our results pave the way to clinically explore SAC-targeting drugs in tumors with high BID expression.

**Supplementary Information:**

The online version contains supplementary material available at 10.1186/s12943-023-01815-w.

## Background

The spindle-assembly checkpoint (SAC), active during mitosis, ensures adequate segregation of sister chromatids by preventing the metaphase-anaphase transition until the spindle microtubules are properly attached to the kinetochores [1]. The signaling activity of the SAC is originated in the mitotic checkpoint complex (MCC), located in the kinetochores, which arrests the cell cycle by inhibiting the anaphase-promoting complex/cyclosome (APC/C) [2]. In addition to the core components of the MCC, other proteins involved in the SAC include Aurora kinase B (AURKB) and the dual specificity protein kinase TTK (also known as MPS1) [3–6].

In contrast to other cell cycle checkpoints, the SAC is an essential device for survival in all metazoan cells, including cancer cells; and agents against TTK, APC/C or AURKB have been developed [7]. Several TTK (TTKi) and AURKB inhibitors (AURKBi) are currently in clinical trials in different malignancies and partial responses have been observed [8–11]. However, the clinical application of anti-SAC agents has been hampered by on-target induced toxicity, resulting in a narrow therapeutic window, and by modest responses that could be attributed to several factors. First, it is known that SAC abrogation leads to abnormal segregation of chromosomes and polyploidy/aneuploidy, but the cell and molecular processes subsequently triggered are poorly understood [7]. In particular, some tumor cells experience cycle arrest and survival and others cell death after SAC abrogation due to factors so far unidentified [12–15]. Second, no predictive markers to select patients for treatment with drugs overriding SAC have been identified [8, 16]. Finally, in the case of AURKB, most of the inhibitors also show some activity against Aurora kinase A (AURKA) and vice versa, resulting in an imperfect differentiation of the effects of AURKA vs. AURKB blockade [17–20].

Here, we show that upregulation of BH3-interacting domain death agonist (BID) associates with sensitivity to SAC abrogation by AURKBi and TTKi, both in vitro and in vivo. We also demonstrate that high levels of BID, which are present in ~ 6% of human tumors, switch an AURKB/caspase-2 (CASP-2) checkpoint from arrest and survival to cell death after SAC abrogation. Our results indicate that BID expression could be a *bona fide* candidate for patient selection, bringing SAC-targeting drugs closer to the clinic.

## Methods

### Patients and tumor samples

The tumor samples used in the study derived from cancer patients diagnosed between 2015 and 2020 in three hospitals in Barcelona (Spain), Hospital Universitario Dexeus, Hospital de la Santa Creu i Sant Pau and Hospital Clínic de Barcelona. Studies were conducted in accordance with the Declaration of Helsinki, under an approved protocol of the Institutional Review Boards of the three participating hospitals (2020/122-ONC-DEX). Samples were de-identified for patient confidentiality and informed written consent was obtained from all subjects.

### Cell culture and animal models

Tumor cells were cultured and authenticated using standard protocols, further details can be found in Supplementary Methods. Parental cell lines used in the study were acquired from different sources (Table S1). Resistant cells were derived by exposing the corresponding parental cells to EGFR TKIs, as described [19, 21] (Figure S1A) and were maintained as polyclonal populations under concentrations of the appropriate drug ≥ 2.5 µM.

Animal studies were performed by Xenopat (Hospitalet de Llobregat, Spain) (NCI-H1819 xenograft study), Champions Oncology (Hackensack, NJ) (PC9-R5 xenograft, CTG-3429, CTG-1059 and CTG-3283 PDXs), XenoSTART (San Antonio, TX) (ST3632 PDX) and Astra Zeneca (Waltham, MA) (DCFI-403, CTG-2939 and DFCI-367 PDXs). All experiments were approved by Astra Zeneca Oncology Animal Welfare Committee. Experiments performed in Xenopat were additionally approved by the Ethical Committee of Animal Experimentation of the Parc Científic de Barcelona (PCB), following the Astra Zeneca Oncology Animal Welfare Criteria and the guidance of the Association for Assessment and Accreditation of Laboratory Animal Care (AAALAC, Unit 1155). Animal experiments were performed using standard protocols, further details can be found in Supplementary Methods.

### Cell assays

For viability assays, cells were seeded at a density of 2000–8000 per well in 96-well plates, allowed to attach for 24 h and treated with drugs for 2–3 doubling times, except otherwise specified. AZD2811 was kindly provided by AstraZeneca (Cambridge, UK), the rest of the drugs were purchased from Selleck Chemicals (Houston, TX) or MedChem Express (Monmouth Junction, NJ). Each concentration of drug was tested in six wells (technical replicates) in each experiment. After treatment, cell mass was estimated by the Thiazolyl Blue tetrazolium bromide (MTT) assay. Drugs and combinations were tested in a minimum of two independent experiments performed in different dates (biological replicates) [22]. Propidium iodide (PI), Annexin V and Beta-galactosidase staining were used for cell cycle, cell death and senescence analyses, respectively, using standard procedures. Phenotypic reversion and essentiality score determinations of transfected cells assays were performed using Incucyte® Live-Cell Analysis System (Sartorius, Gotinga, Germany). Phenotypic reversion refers to PC9-GR3 cells reverting from an apoptotic to a senescent phenotype after AZD2811 treatment when certain genes are knocked-out. For further details about cell assays, see Supplementary Methods.

### Western blot and FISH

Western blotting was performed by standard procedures, as described in Supplementary Methods. The primary antibodies used are listed in Table S2.

FISH was performed with the ZytoLight® SPEC DiGeorge Triple Color Probe and the ZytoLight® SPEC DiGeorge/Phelan McDermid Dual Color Probe (ZytoVision, Bremerhaven, Germany), according to manufacturer’s instructions. The number of color signals per cell in formalin-fixed paraffin embedded (FFPE) cell lines and tumor samples was evaluated by an expert pathologist in a minimum of 100 cells.

### DNA and RNA analyses

DNA and RNA were purified from cultured cells, lymphocytes and FFPE samples using the high purity FFPE RNA isolation kit (Roche Diagnostics, Mannheim, Germany), the GeneRead DNA FFPE Kit, the QIAamp DNA FFPE Tissue Kit, the DNeasy® Blood & Tissue Kit (Qiagen) and the High Pure RNA isolation Kit (Roche Diagnostics), according to the manufacturer’s instructions.

Quantitative PCR (Q-PCR) was used to estimate gene copy numbers. Template genomic DNA was added to Taqman Genotyping Master Mix (Applied Biosystems, Pleasanton, CA) in 10 µl reactions containing specific primers and probes for each gene analyzed (*BID*, *MAPK1*, *CRLK* and *SHANK3*). The primer and probe sets were purchased as Taqman™ Copy Number Assays (Applied Biosystems), *TERT* was selected as a reference gene and acquired as a Taqman™ Copy Number Reference assay. The methodology was validated by comparison with the gold standard (FISH) in a panel of cell lines and FFPE tumor tissues.

DNA-based next generation sequencing (NGS) was performed with the GeneRead® QIAact Lung DNA UMI Panel (Qiagen, Hilden, Germany), according to the manufacturer’s instructions. Results were analyzed using the Clinical Insight Interpret (QCI-I) web. Whole exome sequencing (WES) and whole transcriptome sequencing (WTS) were performed as described [19, 21]. Further details can be found in Supplementary Methods.

RNA expression levels were determined by reverse-transcription quantitative PCR (RT-Q-PCR) and nCounter. For RT-Q-PCR, total RNA was converted into cDNA using the M-MLV reverse transcriptase enzyme (Invitrogen, Carlsbad, CA). Quantification of gene expression was performed using a QuantStudio 6 Flex (Applied Biosystems). Levels of mRNA of different genes were quantified with specific primers and probes (Table S3), according to the comparative ΔCt method [23]. *ACTB* (β-actin) was employed as endogenous gene for normalization. nCounter was performed with a 45-gene custom panel (Table S4), a fusion-specific panel [24] or the IO360 panel, according to the manufacturer’s instructions (Nanostring, Seattle, WA); mRNA levels were subsequently quantified as described [24, 25]. For further details, see Supplementary Methods.

### Gene silencing and ectopic expression

For CRISPR/Cas9-mediated knockout (KO) screening, a sgRNA library targeting 80 genes was designed, synthesized, purified and provided in a 96-well plate by Synthego Corporation (Redwood City, CA, US). CRISPR edited PC9-GR3 screen ready cells were also provided by Synthego Corporation. PC9-ER cells stably expressing Cas9 were generated by the Molecular Cytogenetics and Genome Editing Unit, Centro Nacional de Investigaciones Oncológicas (CNIO) (Madrid, Spain), using transfection with Cas9-blasticidin lentiviral particles followed by blasticidin selection. Expression of Cas9 was tested by RT-Q-PCR and Western blotting. The 80-gene library was lipofected into PC9-ER/Cas9 cells, using the manufacturer’s instructions. Selected colonies were expanded and gene KO verified using RT-Q-PCR and Western blotting. Silencing of *BID* and *CRKL* was also achieved by stable transfection using shRNA Lentiviral Transduction Particles, designed and synthesized by the Molecular Cytogenetics and Genome Editing Unit, Human Cancer Genetics Program, CNIO (Madrid, Spain). Non-target shRNA particles were used as controls. Finally, for ectopic expression of BID, a pTRIPZ-derived vector was designed with the *BID* gene under the control of a doxycycline-inducible promoter and a puromycin resistance gene. The plasmid was encapsulated within lentiviral particles, which were used for cell transfection.

### Statistical analysis

GraphPad Prism v6.0 (GraphPad Software, Inc., La Jolla, USA) was used for all statistical analyses; statistical tests are indicated in the figure and supplementary figure legends. Welch’s correction was applied to Student’s t tests when variances in the two groups under comparison were significantly different by an F test. Data are presented as mean ± SEM or SD, as indicated; *p*-values of < 0.05 were considered statistically significant. In the case of the MTT assays, the average viability of the technical replicates was calculated for every experiment and drug concentration. Then, the average cell viability values of the ≥ 2 independent experiments (biological replicates) were used to calculate the mean ± SD and perform the Student’s t tests, when needed. In the ectopic expression experiments with a doxycycline gradient, results were adjusted using the least squares fit method to a “Michaelis-Menten-like” or saturation kinetics, according to the following equation

$$Growth\ inhibition=\frac{Bmax.\left(BID\right)}{Kd+\left(BID\right)}\;\left(\text{i}\right)$$

Where BID stands for the *BID* mRNA or protein levels while *Bmax* and *Kd* are constants that could be experimentally determined and are presented in the plots.

## Results

### Sensitivity to SAC abrogation in *EGFR*-mut tumor cells does not depend on cell cycle regulators or EGFR signal transduction pathway proteins

**Fig. 1:**
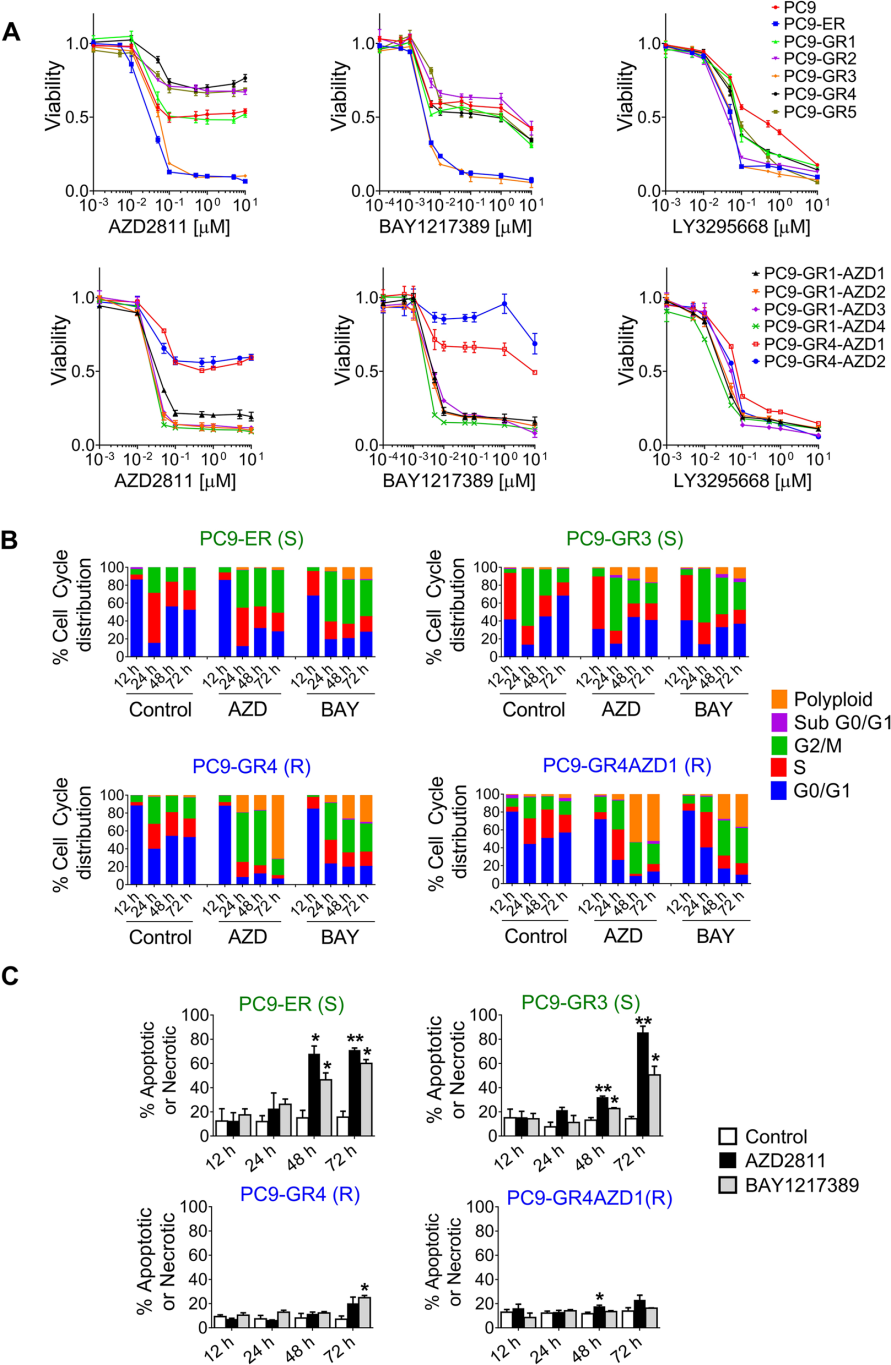
SAC abrogation has different effects in EGFR TKI resistant clones. **A** Dose-response plots to AZD2811 (left), BAY1217389 (middle) and LY3295668 (right panels) of EGFR TKI-resistant clones derived from the PC9 cell line. Values shown are means ± SD of three independent experiments. In each experiment, every concentration of drug was tested in six wells (*n* = 6). **B** Cell cycle analysis of PC9-ER, PC9-GR3, PC9-GR4 and PC-GR4AZD1 clones. Cells were serum-starved for 24 h, FBS (10%) and AZD2811 (150 nM) or BAY1217389 (50 nM) were added, and cultures submitted to PI staining at the indicated times. S (green) and R (blue), clones sensitive and resistant to SAC abrogation. **C** Percentage of apoptotic/necrotic cells in PC9-ER, PC9-GR4, PC9-GR4 and PC-GR4AZD1 cultures. Cells were treated with AZD2811 (150 nM) or BAY1217389 (50 nM) and cultures submitted to annexin V/propidium iodide staining at the indicated times. Bars indicate mean ± SEM of two independent experiments. **p<0.01, *p<0.05 (Student’s t test). S (green) and R (blue), clones sensitive and resistant to SAC abrogation

In previous studies, we had generated a panel of 18 EGFR TKI resistant clones from the *EGFR*-mut, non-small cell lung cancer (NSCLC) lines PC9 and 11–18 (Table S5, Fig. S1A). Unexpectedly, we had found that they showed dual responses to AURKBi but not to other drugs such as MET, AXL or FGFR inhibitors [19]. Here, we used this panel to explore cell fate after SAC abrogation and to investigate markers of sensitivity. First, we tested AZD2811, a drug ~ 3700 fold more potent for AURKB over AURKA in cell-free assays [26] and discovered that twelve of the 18 clones presented resistance (IC50 > 5 µM) while six were extremely sensitive, with IC50s values ≤ 50 nM. We then used BAY1217389, a highly specific TTKi currently in Phase I trials [11, 13, 27], finding a perfect coincidence in cell response with AZD2811; with the six clones sensitive to the AURKBi showing IC50s < 1 nM for the TTKi and the twelve AZD2811-resistant clones presenting IC50s > 200 nM (Fig. 1A and S1B, Table S5). In contrast, the AURKA inhibitor LY3295668 and the DNA-damaging agent cisplatin showed similar efficacy in the 18 clones (Fig. 1A and S1C). Subsequent flow cytometry experiments revealed that, after G2/M arrest, SAC abrogation by AZD2811 or BAY1217389 triggered extensive cell death only in sensitive clones (Fig. 1B-C and S1D).

Next, we determined the protein and mRNA levels of relevant cell cycle proteins in the 18 clones of our panel; including Rb, p21, p53, TTK or AURKB, together with several cyclins and CDKs. Protein levels were analyzed by Western blotting (Fig S 2) and mRNA expression by nCounter, using a 45-gene custom panel (Table S4). We did not find any consistent differences between clones sensitive and resistant to SAC abrogation (Figs. S2 and S3A, Table S6). We also tried to develop multi-gene expression signatures of sensitivity to SAC abrogation by using hierarchical and non-hierarchical clustering methods, selecting subsets of genes functionally related or comparing gene expression levels in presence and absence of AZD2811, but our attempts systematically failed (Fig. S3B). In addition, flow cytometry did not reveal any association of early entry into the S phase or a shorter doubling time with response to AURKBi/TTKi, although some sensitive clones showed a higher G2/M subpopulation 24 h after reentry into cell cycle (Table S5, Fig. S3C, D). Finally, the levels and phosphorylation of key proteins in the EGFR pathway did not correlate with cell fate after SAC abrogation (Fig. S2) and the combination of a MEK or a PI3K inhibitor with AZD2811 did not render resistant clones responsive to AURKB inhibition (Fig. S4).

### Sensitivity to SAC abrogation is associated with acquired Chr22q11 amplification in EGFR TKI resistant cells

**Fig. 2:**
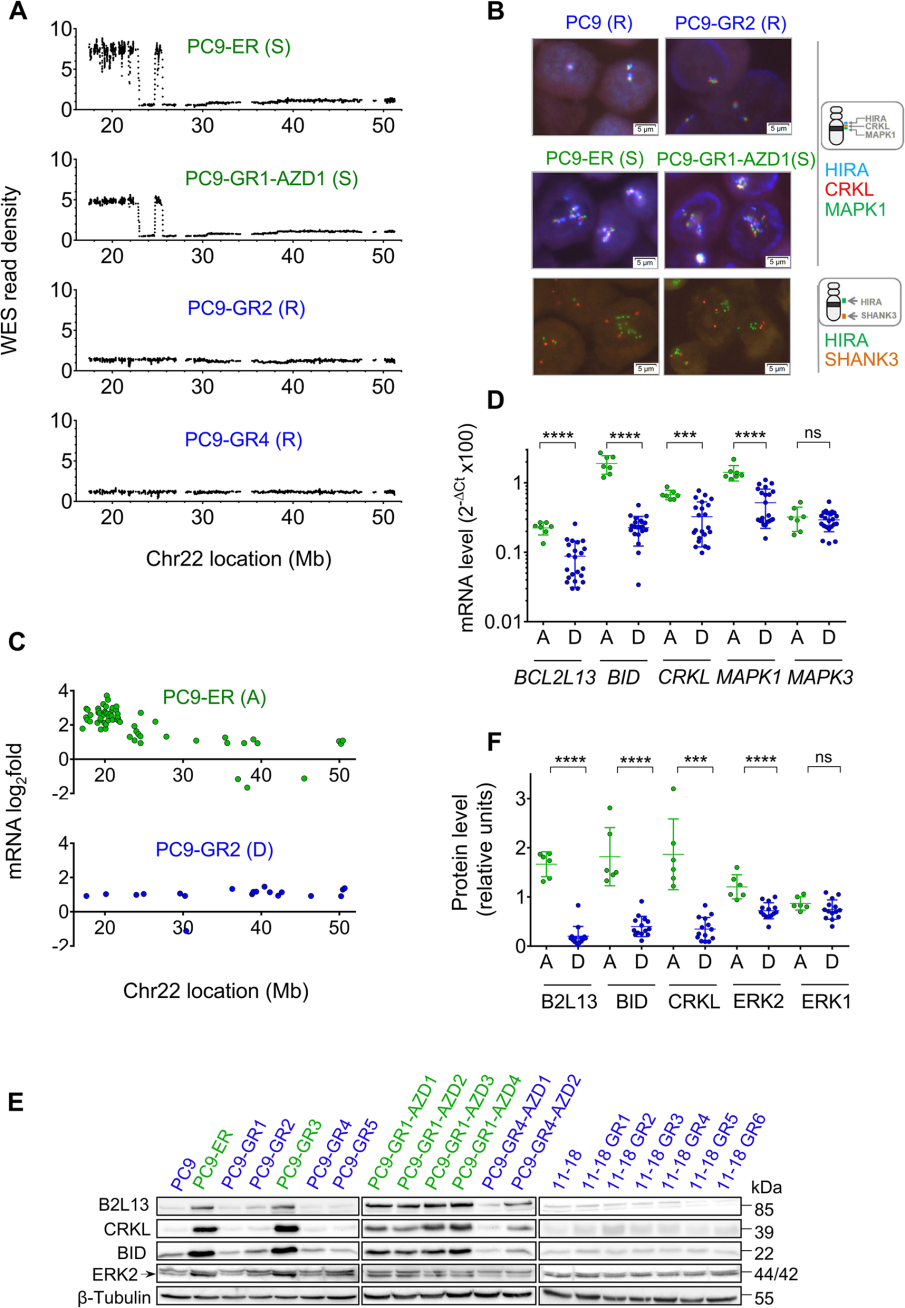
Sensitivity to SAC abrogation in EGFR TKI resistant clones is associated with Chr22q11 amplification and upregulation of Chr22q11 genes. **A** Chromosome 22 WES read density maps of four representative PC9-derived clones, sensitive (S, green) and resistant (R, blue) to SAC abrogation by AURKBi and TTKi. Data were normalized using the parental cell lines. **B** FISH analysis of representative PC9-derived clones sensitive (S, green) and resistant (R, blue) to SAC abrogation. Probes for the three Chr22q11 genes *HIRA*, *CRKL* and *MAPK1* (upper and middle panels) or for *HIRA* and the Chr22 telomeric gene *SHANK3* (lower panels) were used. **C** Chr22 genes significantly up- or down-regulated by Whole Transcriptome Sequencing (WTS) in two representative PC9-derived clones with Chr22q11 amplification (A, green) or diploid (D, blue). Data were normalized against parental PC9 as explained in Supplementary Methods. **D** mRNA levels of four Chr22q11 genes (*BCL2L13*, *BID*, *CRKL* and *MAPK1*) in PC9 and 11-18 derived clones, classified according to Chr22q11 status; amplified (A), green vs. diploid (D), blue. RT-Q-PCR was used for mRNA quantification, data were normalized against beta-actin. A fifth gene not in Chr22 (*MAPK3*) was included as a control. Each point represents a clone, lines indicate mean ± SD. ****p<0.0001, ***p<0.001; ns, not significant (Student’s t test). **E** Western blotting analysis of four proteins coded by Chr22q11 genes in PC9 and 11-18-derived clones. B2L13 is the protein codified by the *BCL2L13* gene. Erk2 (codified by *MAPK1*) corresponds to the lower band of the doublet and is indicated by an arrow, the upper band is Erk1 (codified *by MAPK3*). Green, clones with Chr22q11 amplification, sensitive to AURKBi and TTKi; blue, clones diploid for Chr22q11, resistant to both drugs. **F** Levels of four Chr22q11-codified proteins (B2L13, BID, CRKL and Erk1) in PC9 and 11-18 derived clones, quantified from the Western blotting image presented in (H). The intensity of the bands was normalized using β-tubulin. Clones are classified according to Chr22q11 status; A, amplified, green vs. D, diploid, blue. The Erk1 protein, not codified by a Chr22 gene, was included as a control. Each point represents a clone, lines indicate mean ± SD. ***p<0.001, **p<0.01, *p<0.05; ns, not significant (Student’s t test)

Next, we performed Whole Exome Sequencing (WES) on the 18 clones of our panel, together with the parental PC9 and 11–18 cell lines. We identified an amplification of the 11q region in chromosome 22 exclusively in the six clones sensitive to AURKBi and TTKi (Fig. 2A and S5, Table S7). FISH confirmed amplification of Chr22q11 in all the WES-positive clones, with 7–10 copies and a ratio ≥ 2 to the telomere (Fig. 2B and S 6, Table S8). We then analyzed 12 additional *EGFR*-mut, TKI-resistant clones generated in different laboratories (Table S9). Two of them (PC9-R5 and PC9-OR4) were sensitive to SAC abrogation; amplification of the Chr22q11 region was found in both by FISH and/or NGS (Fig. S6; Tables S7, S9). Although the eight clones with Chr22q11 amplification shared PC9 cells as the common ancestor, their detailed phylogeny suggested several independent amplification events (Fig. S7A). The boundaries of the amplified region in Chr22q11 were not fully coincident in all clones and microscopic examination of 3,800 parental PC9 cells did not reveal any case of Chr22q11 gain (Tables S7-S8), further reinforcing this hypothesis.

A comparison of sensitivity profiles to several drugs in the EGFR TKI-resistant clones revealed that Chr22q11 amplification was specifically associated with response to SAC abrogation, with a > 200-fold difference in IC50 for AZD2811 and BAY1217389 between Chr22q11-positive and negative clones; compared to < 2-fold for LY3295668 and no significant differences for cisplatin (Fig. S7B, Table S5). In a previous study we had found that the PC9-ER clone (Chr22q11-amplified) was sensitive to AURKBi not only in vitro but also in xenografts, while the PC9-GR4 (diploid) was resistant in both settings [19]. In this study, the PC9-R5 clone was used for an additional in vivo selection experiment since, according to FISH, it was a mixed population with ~ 65% of cells carrying the Chr22q11 amplification (Table S9). Copy numbers of Chr22q11 were preserved in xenografts treated with osimertinib, while SAC abrogation by AZD2811 selectively eliminated the amplified cells, as expected (Fig. S7C).

### Chr22q11 amplification is associated with mRNA and protein upregulation of the corresponding genes, particularly *BID*

Whole transcriptome sequencing (WTS) revealed extensive gene upregulation associated with Chr22q11 amplification (Fig. 2C and S8A, Table S10), which was confirmed by RT-Q-PCR of four Chr22q11 genes (*BCL2L13*, *MAPK1*, *CRKL* and *BID*) (Fig. 2D and S 8B). The levels of *BID* mRNA were found to be particularly high in positive vs. non-amplified clones (Fig. S8C).

Re-analysis of previous proteomics results [21] showed widespread overexpression of Chr22q11 proteins exclusively in amplified clones. Among the top 40 upregulated proteins in the Chr22q11-positive PC9-ER and PC9-GR3 cells, 17 (42.5%) and 15 (37.5%) corresponded to Chr22q11 genes; which only represent 1.5% (350/23,000) of the human coding genome (Fig. S8D, Table S10). Also, Western blotting analysis of B2L13 (coded by *BCL2L13*), CRKL, BID and ERK2 (coded by *MAPK1*) revealed protein upregulation exclusively in the amplified clones (Fig. 2E, F).

### Silencing of *BID* renders *EGFR*-mut cells with Chr22q11 amplification resistant to SAC abrogation

**Fig. 3:**
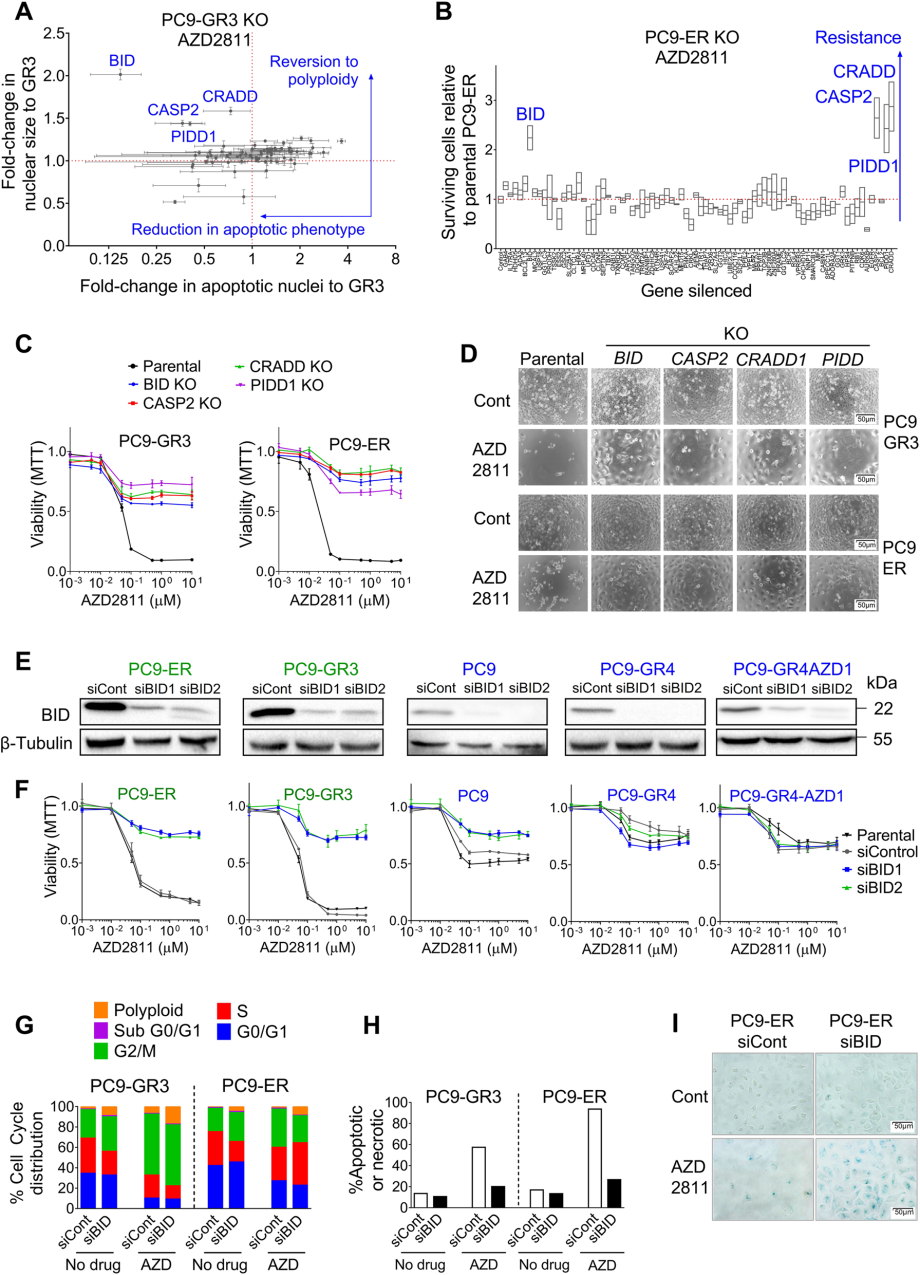
BID expression drives sensitivity to SAC abrogation in cells with Chr22q11 amplification. **A** PC9-GR3 cells (AURKBi/TTKi sensitive) were electroporated with a CAS9-gRNA ribonucleoprotein knock-out (KO) CRISPR library targeting genes located in Chr22q11 (Table S11). Resulting clones were treated with AZD2811 and submitted to a functional assay to determine apoptosis and polyploidy, as described in Methods. Values shown are means and range (*n*=2). **B** PC9-ER cells (AURKBi/TTKi sensitive) stably expressing Cas9 were lipofected with the same CRISPR library; the resulting clones were treated with AZD2811 and cell viability determined by MTT at 72 h. Viability data were normalized against the viability observed for PC9-ER Cas9 parental cells treated with AZD2811. A value >1 indicates that a lipofected clone is more resistant to the compound than the parental PC9-ER cells. Values shown are means and range (*n*=2). **C** Dose-response curves for AZD2811 of PC9-GR3 and PC9-ER cells CRISPR-KO for selected genes. Cells numbers were determined by MTT at 72 h. Values shown are means ± SD of ≥2 independent experiments. In each experiment, every concentration of drug was tested in six wells (*n* = 6). **D** Micrographs of PC9-GR3 and PC9-ER cells CRISPR-KO for selected genes. AZD2811 was added at 150 nM. **E** PC9-ER, PC9-GR3, PC9, PC9-GR4 and PC9-GR4AZD1 cells were transfected with lentiviral particles for shRNA-based silencing of *BID*. After puromycin selection, selected colonies were analyzed by Western blotting. Chr22q11-positive and negative cells are indicated in green and blue, respectively. **F** Dose-response curves for AZD2811 of PC9-ER, PC9-GR3, PC9, PC9-GR4 and PC9-GR4AZD1 colonies with shRNA-based silencing of* BID*. Cells numbers were determined by MTT at 72 h. Values shown are means ± SD of ≥2 independent experiments. In each experiment, every concentration of drug was tested in six wells (*n* = 6). **G** Cell cycle analysis of PC9-GR3 and PC9-ER colonies with shRNA-based silencing. Cells were allowed to attach for 24 h, AZD2811 (150 nM) was added and cultures submitted to PI staining at 72 h. **H** Percentage of apoptotic/necrotic cells in PC9-GR3 and PC9-ER colonies with shRNA-based silencing. Cells were treated as in (G) and submitted to Annexin V staining. **I** Acidic beta-galactosidase staining of PC9-ER with shRNA-based silencing after a 72 h treatment with AZD2811

A literature search revealed that none of the ~ 350 genes in the Chr22q11 segment had a known association with response to SAC abrogation. Consequently, we performed CRISPRn screening in two Chr22q11-amplified, AZD2811 sensitive clones (PC9-GR3 and PC9-ER) using a library targeting 72 relevant, protein-coding Chr22q11 genes (Table S11). The library also included *EGFR*, *CDK6*, *RB1*, *AURKB*, *TTK, BCL2* and, since AURKB has been described to interact with CASP-2 [28], the three genes coding for the components of the PIDDosome; *CASP2*, *PIDD1* and *CRADD* [29]. Transfected cells were analyzed by two different methods. In the case of PC9-GR3, an assay measuring apoptosis and nuclear size revealed that *BID* KO switched cells from apoptosis to polyploidy in response to SAC abrogation by AZD2811. KO of the three components of the PIDDosome had similar effects, while the abrogation of the rest of the genes in the library did not result in phenotype reversal (Fig. 3A). In the case of PC9-ER, viability assays revealed that only KO of *BID*, *CASP2*, *PIDD1* and *CRADD* rendered cells resistant to AZD2811 (Fig. 3B). KO of *AURKB* in both PC9-ER and PC9-GR3 was found to be quickly lethal, confirming the AURKB dependency identified with AZD2811. In agreement with DeepMap data (Table S11), PC9-ER colonies KO for *ESS2*, *UFD1*, *CDC45*, *PI4KA* and *CRKL* also showed impaired growth and subcultures were not viable (Fig. S9A). Additional experiments in PC9-GR3 revealed that *CRKL* contributes to resistance to EGFR TKIs in Chr22q11-positive cells (Fig. S9B-E).

Dose-response curves confirmed the shift to complete resistance to AZD2811 and BAY127389 in PC9-GR3 and PC9-ER after *BID*, *CASP2*, *PIDD1* and *CRADD* KO, with IC50s increasing to > 10 µM. In contrast, cells remained relatively sensitive to LY3295668 and no changes were observed in response to osimertinib (Figs. 3C, D and S10A-C). To further investigate the role of BID in sensitivity to SAC abrogation, we used shRNA-*BID* lentiviruses. MTT assays revealed that *BID* silencing abolished sensitivity to AZD2811 in Chr22q11-positive cells but had a limited effect on response to LY3295668 (Figs. 3E, F, S10D, E). Flow cytometry showed no effects on cell cycle distribution while Annexin V and beta-galactosidase staining demonstrated that AZD2811 triggered senescence instead of apoptosis in Chr22q11-positive, *BID*-silenced colonies (Fig. 3G-I, S11A); which showed a response to SAC abrogation indistinguishable from the response previously observed in non-amplified clones (Fig. 1A-C and [19]).

### Ectopic expression of *BID* renders Chr22q11 negative cells sensitive to SAC abrogation in a dose-dependent manner

**Fig. 4:**
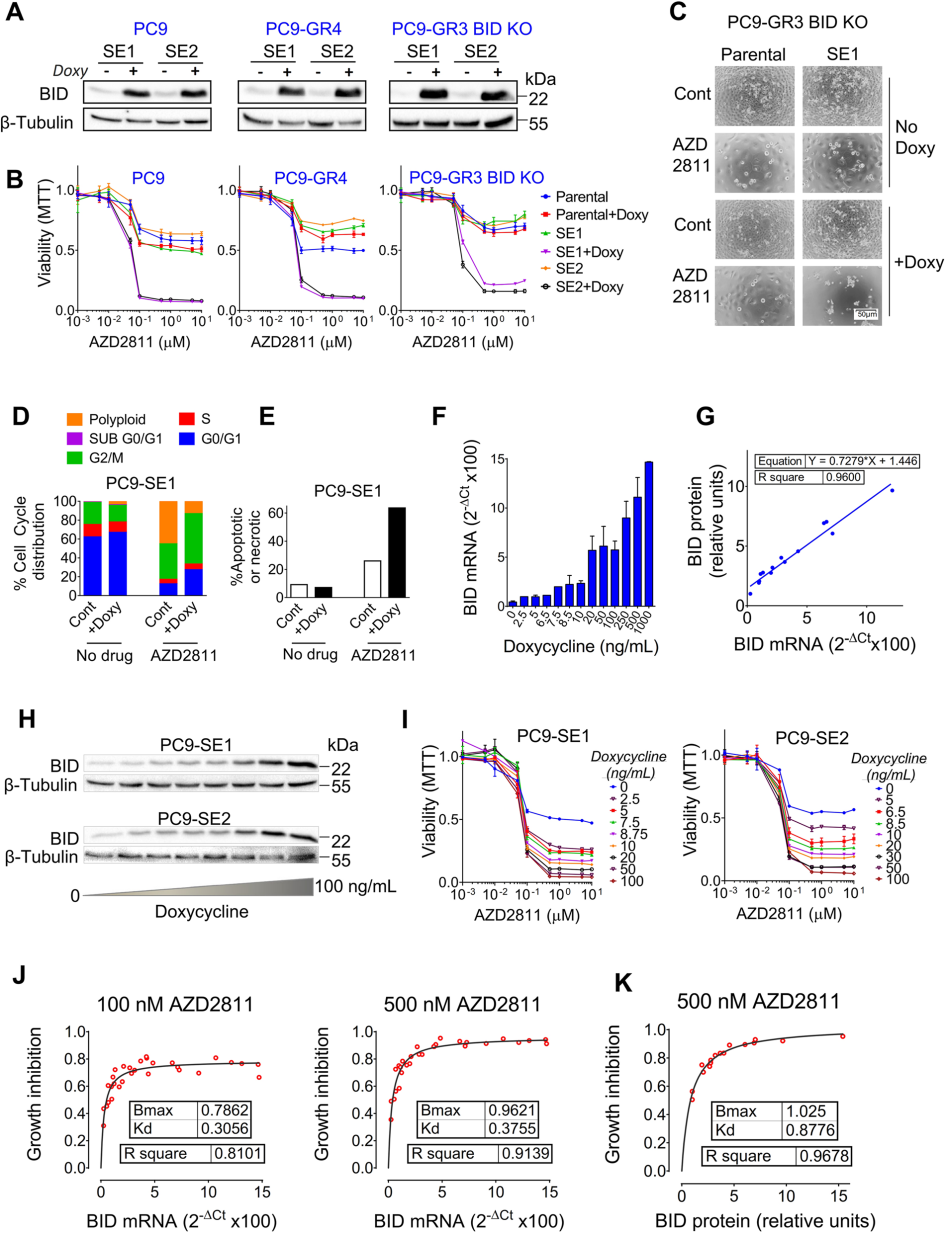
Ectopic expression of *BID* sensitizes Chr22q11 negative cells to SAC abrogation in a dose-dependent manner. **A** PC9, PC9-GR4 and PC9-GR3 BID KO cells (all of them resistant to AURKBi/TTKi) were transfected with a pTRIPZ lentiviral vector with the *BID* gene under the control of a doxycycline-inducible promoter. Two transfected colonies per cell type (SE1 and SE2) were cultured in presence of doxycycline (1 µg/mL), Western blotting was used to confirm ectopic expression of the BID protein. **B** Dose-response curves to AZD2811 of PC9, PC9-GR4 and PC9-GR3 BID KO cells transfected with the pTRIPZ *BID* vector (SE1 and SE2 colonies). Ectopic expression of BID was induced using 1 µg/mL doxycycline. Cells numbers were determined by MTT at 72 h. Values shown are means ± SD of ≥2 independent experiments. In each experiment, every concentration of drug was tested in six wells (*n* = 6). **C** Micrographs of PC9-GR3 BID KO parental and pTRIPZ *BID*-transfected cells (clone SE1) treated with AZD2811 (150 nM) in the presence and absence of 1 µg/mL doxycycline. **D** Cell cycle analysis of PC9-SE1 cells treated with AZD2811 (150 nM) for 72 h, in the absence and presence of 1 µg/mL doxocyclin. **E** Percentage of apoptotic/necrotic cells by Annexin V analysis in PC9-SE1 cells treated with AZD2811 (150 nM) for 72 h, in the absence and presence of 1 µg/mL doxycycline. **F** Effect of increasing concentrations of doxycycline on the ectopic expression of *BID* mRNA by PC9-SE1 and SE2 clones, as determined by RT-Q-PCR. **G** Correlation between the levels of BID protein and *BID* mRNA ectopic expression in PC9-SE1 and SE2 colonies represented in (F) and (H). **H** Effect of increasing concentrations of doxycycline on the ectopic expression of BID protein by PC9-SE1 and SE2 colonies, as determined by Western blotting. **I** Dose-response curves for AZD2811 of PC9-SE1 (left panel) and PC9-SE2 (right panel) clones, ectopic expression of BID protein was induced with increasing concentrations of doxycycline as shown. Cells numbers were determined by MTT at 72 h. Values shown are means ± SD of ≥2 independent experiments. In each experiment, every concentration of drug was tested in six wells (*n* = 6). **J** Plot of *BID* mRNA levels vs. cell growth inhibition at 100 nM AZD2811 (left panel) and 500 nM AZD2811 (right panel) in PC9-SE1 and SE2 cells. **K** Plot of BID protein levels vs. cell growth inhibition at 500 nM AZD2811 in PC9-SE1 and SE2 cells

Next, we determined the effects of BID ectopic expression in Chr22q11-negative, AURKBi/TTKi-resistant cells using a pTRIPZ lentiviral vector wherein *BID* gene expression was under the control of a doxycycline-inducible promoter. (Figs. 4A, S11B). Viability assays indicated that transfected colonies became extremely sensitive to SAC abrogation in presence of doxycycline, with IC50s ~ 50–100 nM for AZD2811 and a shift from polyploidy to apoptosis after G2/M arrest (Fig. 4B-E). In contrast, minor or no effects were observed on response to LY3295668 (Fig. S11C).

We then investigated the kinetics of the association of BID expression with response to SAC abrogation. To this end, we first tested a doxycycline gradient in two transfected PC9 colonies and found that *BID* mRNA and protein expression showed a statistically significant linear correlation (r^2^ = 0.96), being both dependent on doxycycline concentration (Fig. 4F-H). Next, we found that sensitivity to AZD2811 in transfected cells was also dose-dependent on doxycycline, with 50–100 ng/mL of antibiotic inducing the maximum inhibitory effect (Fig. 4I). Finally, when we plotted the *BID* mRNA or protein levels vs. the percentage of growth inhibition by AZD2811 at each point of the doxycycline gradient, we observed a “Michaelis-Menten-like” or saturation kinetics (r^2^ = 0.91 and 0.97) (Fig. 4J-K, see also Methods).

### BID expression determines response to SAC abrogation in cancer cell lines of diverse origins

**Fig. 5:**
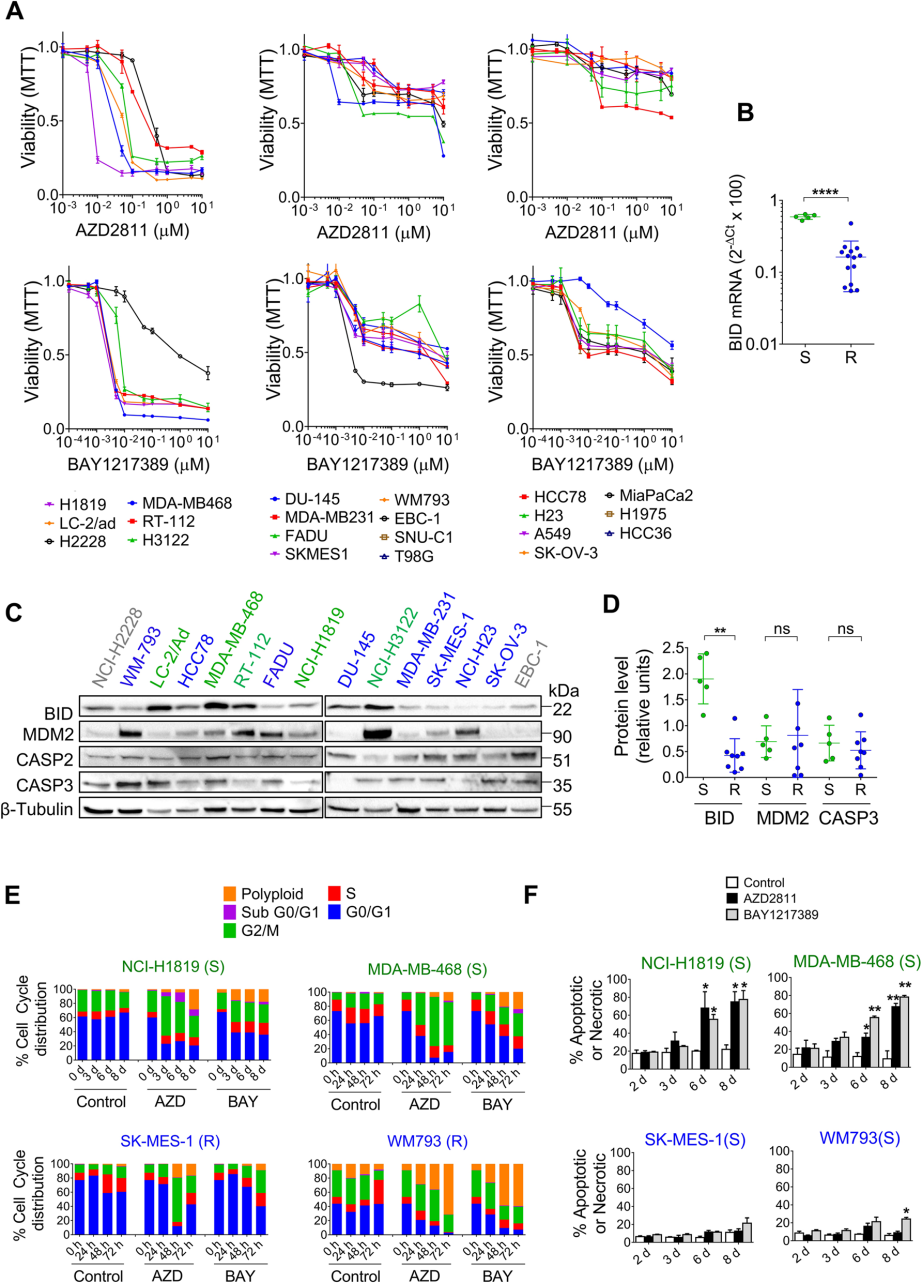
High expression of BID associates with sensitivity to SAC abrogation in cancer cell lines of diverse origins. **A** Dose-response plots to AZD2811 (upper panels) and BAY1217389 (lower panels) of cancer cell lines of lung, prostate, breast, pancreas, ovarian, colon and other origins. Values shown are means ± SD of ≥2 independent experiments. In each experiment, every concentration of drug was tested in six wells (*n* = 6). **B** *BID* mRNA levels in cancer cell lines of diverse origins. Cells classified according to response to SAC abrogation in S, sensitive (*n*=5) and R, resistant (*n*=14) to both AURKBi and TTKi. Each point represents a cell line, lines indicate mean ± SD. ****p<0.0001 (Student’s t test). **C** Western blotting analysis of BID, MDM2 and CASP-3 protein levels in cancer cell lines of diverse origins. The seven cell lines sensitive to AURKBi and/or TTKi, together with eight resistant cell lines, were selected for the analysis. Cells sensitive and resistant to both AURKBi and TTKi are indicated in green and blue, respectively. The two cell lines sensitive exclusively to one of the inhibitors are indicated in grey. **D** BID, MDM2 and CASP3 protein levels in cancer cell lines of diverse origins, quantified from the Western blotting image presented in (C). The intensity of the bands was normalized using β-tubulin. Cells are classified according to response to SAC abrogation in S, sensitive (*n*=5) and R, resistant (*n*=14) to both AURKBi and TTKi. Each point represents a cell line, lines indicate mean ± SD. **p<0.001; ns=not significant (Student’s t test). **E** Cell cycle analysis of the NCI-H1819, MDA-MB-468, SK-MES-1 and WM793 cultures. Cells were allowed to attach, serum starved for 24 h, FBS (10%) and AZD2811 (150 nM) or BAY1217389 (50 nM) were added and cultures submitted to PI staining at the indicated times. Lines sensitive (S) and resistant (R) to SAC abrogation are indicated in green and blue, respectively. **F** Percentage of apoptotic/necrotic cells in NCI-H1819, MDA-MB-468, SK-MES-1 and WM793 cultures. Cells were treated with AZD2811 (150 nM) or BAY1217389 (50 nM) and cultures submitted to annexin V/PI staining at the indicated times. Bars indicate mean ± SD of two independent experiments. *p<0.05 (Student’s t test). Lines sensitive and resistant to SAC abrogation are indicated in green and blue, respectively

The experiments presented so far firmly established the association of BID expression with response to SAC abrogation in *EGFR*-mut, NSCLC cells. Next, we decided to investigate tumor cells of other origins and genetic backgrounds. To this end, we selected 21 lines derived from lung, breast, pancreas, bladder, prostate, ovarian, head and neck and brain carcinomas and determined the dose-response curves to AZD2811 and BAY1217389 (Fig. 5A, Table S12). Five of the 21 cell lines were sensitive to both drugs; experiencing extensive cell death under visual inspection and showing IC50s < 300 nM for AZD2811 and < 7 nM for BAY1217389. Two of them were fusion-positive lung adenocarcinoma cell lines; the other three were of breast, bladder and lung origin and did not harbor known oncogenic drivers (Table S13). In contrast, 14 cell lines were resistant to both AZD2811 and BAY1217389, with IC50s > 1 µM and > 50% survival even at high concentrations of drugs. Finally, two cell lines were sensitive to one of the SAC-targeting agents but not to the other. When we determined the effects of LY3295668 in the same panel, we found that some cell lines sensitive to SAC abrogation were resistant to the AURKAi (RT-112, NCI-H1819) and vice versa (HCC366, NCI-H23, SK-MES-1) (Fig. S12A, Table S12).

The five cell lines sensitive to AZD2811 and BAY1217389 expressed significantly higher levels of *BID* mRNA and protein than the rest of the panel (Figs. 5B-D and S12B). In fact, among the 6 lines with high *BID* mRNA (2^−∆Ct^x100 ≥ 0.5) and protein, only the HCC78 cells were not sensitive to SAC abrogation; while the 15 lines with low *BID* (2^−∆Ct^x100 ≤ 0.3) were uniformly resistant to both AZD2811 and BAY1217389 (Fig. S 12B, Table S13). Interestingly, the two cell lines sensitive only to one drug had intermediate *BID* levels (2^−∆Ct^x100 ~ 0.3). In contrast, the expression of other proteins such as MDM2 or CASP3 did not correlate with sensitivity to SAC abrogation (Fig. 5D) and *BID* upregulation did not associate with response to LY3295668 (Fig. S12C). Remarkably, when we plotted the *BID* mRNA levels vs. the growth inhibition by AZD2811 and BAY1217389 of all the and *EGFR*-mut clones and *EGFR*-wt cell lines included in our study, a saturation kinetics was observed again; with r^2^ = 0.75 and 0.58, respectively (Fig. S12D).

Regarding Chr22q11 status; Q-PCR of three Chr22q11 genes (*BID*, *CRKL* and *MAPK1*) revealed *BID* copy number gains only in 2/21 cell lines (Fig. S13A-C). As previously described [30], NCI-H1819 cells were found to harbor > 6 copies of *BID* and *CRKL* but not *MAPK1* by Q-PCR, a result that was confirmed by FISH for *CRKL* and *MAPK1* (Fig. S13C, D); while the prostate DU-145 cells showed four copies of *BID* but were diploid for *MAPK1* and *CRKL*. Interestingly, NCI-H1819 but not DU-145 cells overexpressed *BID* according to the cut-off mentioned above (2^−∆Ct^x100 ≥ 0.5) and showed sensitivity to SAC-targeting agents (Fig. 5A).

**Fig. 6:**
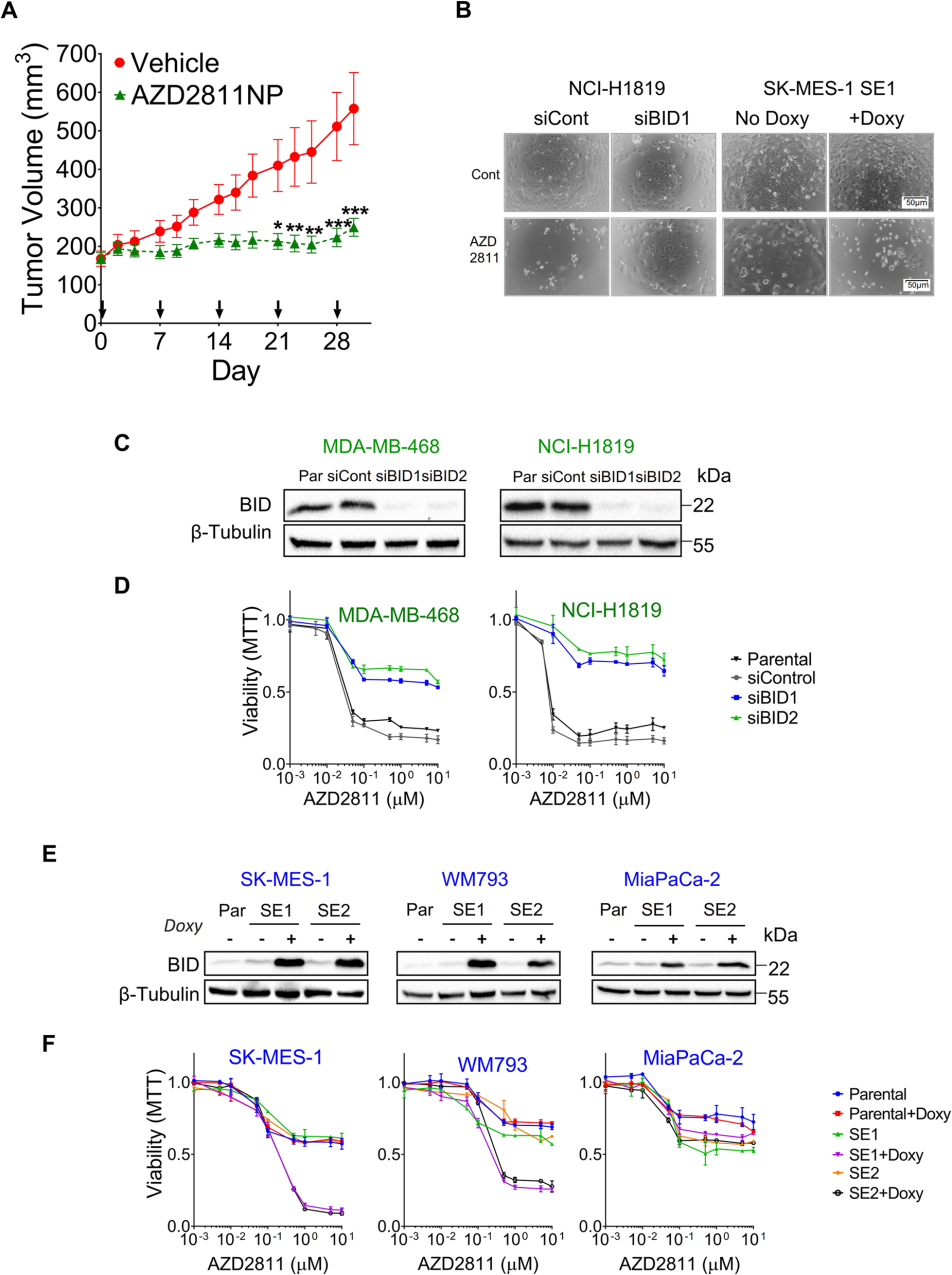
High expression of BID associates with sensitivity to SAC abrogation in cancer cell lines of diverse origins (II). **A** Athymic nude mice bearing NCI-H1819 xenografts were treated with vehicle or AZD2811 nanoparticle formulation (25 mg/kg) once weekly, as indicated by arrows. Tumor volumes were measured by caliper (mean ± SEM, *n*=9 for each group). Two-way RMANOVA and Bonferroni post-hoc test detected significant differences in Vehicle vs AZD2811 from Day 21 (Day 21,*p<0.05; Days 23 and 25, **p<0.01; Days 28 and 30, ***p<0.001). **B** Micrographs of SK-MES-1 and NCI-H1819 transfected cells, as explained in (I). AZD2811 was used at 150 nM and doxycycline at 1 µg/mL. **C** The AURKBi/TTKi-sensitive MDA-MB-468 and NCI-H1819 cells were transfected with shRNA lentiviral particles to silence *BID. *After puromycin selection, colonies were analyzed by Western blotting. **D** Dose-response plots to AZD2811 of the colonies selected in (C). **E** The AURKBi/TTKi-resistant MiaPaCa-2, SK-MES-1 and WM793 cell lines were transfected with the pTRIPZ-*BID* vector. After puromycin selection, colonies were analyzed by Western blotting. Doxycycline at 1 µg/mL was used to induce ectopic expression of BID. F) Dose-response plots to AZD2811 of the colonies selected in (E)

Flow cytometry of NCI-H1819, MDA-MB-468, SK-MES-1 and WM793 cells revealed that SAC abrogation by AZD2811 and BAY1217389 increased the fraction of cells in G2/M or aneuploid/polyploid; while Annexin V indicated subsequent, extensive induction of cell death in the BID-high NCI-H1819 and MDA-MB-468 but not in the BID-low SK-MES-1 and WM793 (Fig. 5E-F). Xenograft experiments showed significant inhibition in the subcutaneous growth in NCI-H1819 tumors treated with AZD2811 nanoparticles, without any effect on body weight (Figs. 6A and S13E, F). The Chr22q11 amplification was still present at the end of the experiment in vehicle-treated tumors and primary cultures derived from them maintained sensitivity to AZD2811 (Fig. S13D and G). In addition, published studies had showed that the BID-overexpressing MDA-MB-468 breast cancer cells established in nude mice as xenografts were sensitive to the TTK inhibitors CFI-402,257 [16] and BAY1217389 [14, 31].

To further confirm that BID is a general regulator of response to SAC abrogation in tumor cells, we used two approaches; (i) BID silencing with shRNA lentiviruses in two AURKBi/ TTKi-sensitive cell lines, one of them harboring the Chr22q11 amplification (NCI-H1819) and another diploid (MDA-MB-468) and (ii) BID ectopic expression with the pTRIPZ-*BID* vector described above in resistant cell lines of different origins and genotypes; MiaPaCa2 (*KRAS G12C*, pancreas), SK-MES-1 (wt for known drivers, lung squamous) and WM793 (*BRAF V600E*, melanoma). We found that *BID* silencing rendered NCI-H1819 and MDA-MB-468 cells resistant to AURKBi and TTKi, with no effects on response to AURKAi (Figs. 6B-D, S14A-C). Conversely, doxycycline-induced BID expression triggered sensitivity to AZD2811 but not to LY3295668 in SK-MES-1 and the *BRAF*-mut WM793 cells (Figs. 6E, F, S14D, E). Finally, in the *KRAS*-mut MiaPaCa-2 cells, *BID* mRNA levels in presence of doxycycline were significantly lower than in SK-MES-1 or WM793 (Fig. S14D) and cells remained resistant to AZD2811 (Fig. 6F).

### SAC abrogation triggers cell death through a pathway involving BID, CASP-2 and CASP-3

**Fig. 7:**
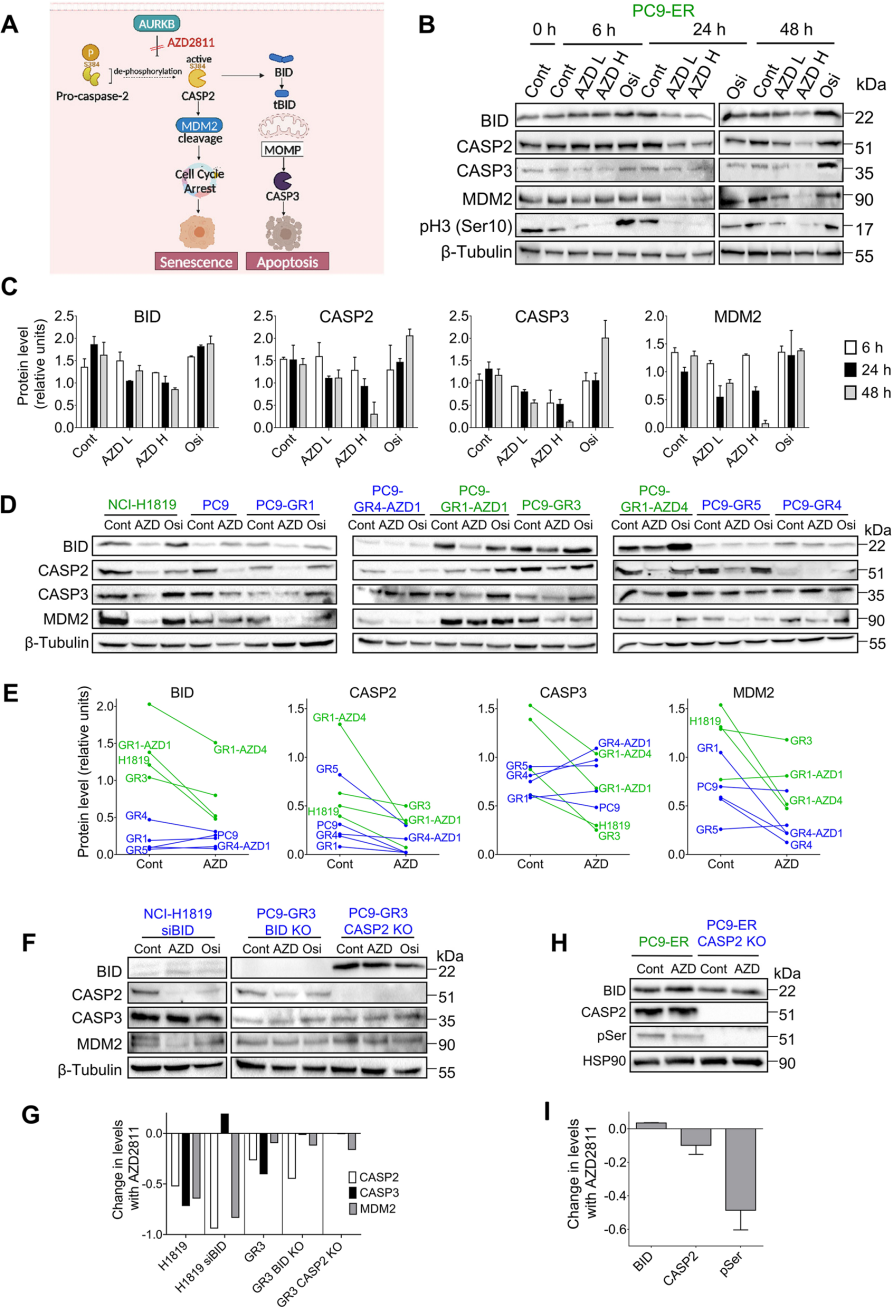
SAC abrogation triggers cell death through a pathway involving BID, CASP-2 and CASP-3. **A** Schematic depicting the hypothetical transduction pathways triggered by TTK and AURKB inhibitors in cancer cells. Modified from [28]. **B** PC9-ER cells, sensitive to SAC abrogation, were treated with the AZD2811 150 nM (AZD L), AZD2811 500 nM (AZD H) or Osimertinib 500 nM (osi) and selected proteins of the pathway presented in (**A**) were assessed by Western blotting. The images shown are a representative of two different experiments. **C** Quantification of the immunoblots shown in (**B**). Bars represent mean ± SEM of two independent experiments. **D** Parental PC9, seven PC9-derived clones and NCI-H1819 cells were treated with Osimertinib 500 nM (osi) or AZD2811 150 nM (AZD) for 24 h (48 h for NCI-H1819) and selected proteins of the pathway presented in (**A**) were analyzed by Western blotting. Cells sensitive and resistant to SAC abrogation are indicated in green and blue, respectively. **E** Quantification of the immunoblots shown in (**D**). The intensity of the bands was normalized against β–tubulin. **F** NCI-H1819 cells silenced for *BID* and PC9-GR3 cells knocked-out for *BID* or *CASP2* were treated with AZD2811 (150 nM) or osimertinib (500 nM) and selected proteins assessed by Western blotting. **G** Quantification of the bands in the Western blottings in (**D**) and (**F**), showing changes in protein levels after AZ2811 treatment. The intensity of the bands was normalized against β–tubulin. **H** Parental PC9-ER cells and PC9-ER CASP-2 KO were treated with AZD2811 (150 nM) and assessed by Western blotting. **I** Quantification of the bands corresponding to PC9-ER parental cells in the Western blotting shown in (**H**). The intensity of the bands was normalized against HSP90

Sustained aberrant mitosis has been reported to ultimately reduce AURKB activity, enabling CASP-2 dephosphorylation at S384 and subsequent activation [28]. Activated CASP-2 can then induce MDM-2 cleavage and cell cycle arrest or caspase-3 (CASP-3) mediated apoptosis. In addition, TTK has been demonstrated to activate AURKB through phosphorylation of Borealin [5]. Based on these published data and the results presented thus far; we hypothesized that SAC-targeting drugs activate CASP-2 in tumor cells and ultimately trigger CASP-3 mediated apoptosis or aneuploidy/polyploidy and survival, the balance between the two outcomes being determined by BID levels (Fig. 7A).

We performed a series of experiments to validate this hypothesis. First, we combined AZD2811 and BAY1217389 with navitoclax, a compound that inhibits pro-survival bcl-2 family proteins, mimicking the effect of BH3-only proteins such as BID [32]. We found that the interaction was almost invariably synergistic in cells sensitive to SAC abrogation. In contrast, the BH3 mimetic had no effect on response to osimertinib in cells sensitive to this drug (Figs S15, S16A). We also tested Q-VD-Oph, a pan-caspase inhibitor targeting CASP-1, 3, 8 and 9 [33] and found that it suppressed cell death induced by AZD2811 and BAY1217389 in the BID-high PC9-ER and by AD2811 in NCI-H1819 cells (Fig. S16B).

Next, a time-course experiment in PC9-ER cells revealed a decrease in full-length BID, CASP-2, CASP-3 and MDM2 levels by Western blotting, indicative of cleavage, starting after 24 h of treatment with AZD2811. Osimertinib, which was tested in parallel, failed to induce any of these effects (Fig. 7B, C). Further testing in additional clones and cell lines revealed that AZD2811 but not osimertinib triggered a widespread reduction in full-length CASP-2 and MDM2 at 24 h, independently of BID expression levels. In contrast, the AURKBi only induced a detectable decrease in full-length BID and CASP-3 in cells with high BID (Fig. 6D, E), while osimertinib failed to show these effects (Fig. S16C). We also found that AZD2811 did not alter BID or CASP-3 levels in *CASP2* KO cells while, in *BID* KO cells, the AURKBi effectively reduced full-length CASP-2 but failed to trigger CASP-3 cleavage (Fig. 7F, G). Finally, we observed a 48-kDa band recognized by anti-phospho-Ser antibodies in PC9-ER but not in CASP-2 KO cells. After treatment with AZD2811 for 6 h, the intensity of the band was significantly reduced while total CASP-2 was unaltered, indicating dephosphorylation of CASP-2 serine residues prior to cleavage (Fig. 7H, I). However, since there are no commercially available antibodies specific for pSer386, we could not determine if AZD2811 triggered CASP-2 dephosphorylation in this particular position. Two lines of evidence indicate that the CASP-2 dephosphorylation observed after AURKB inhibition is specific and not a consequence of widespread changes in protein phosphorylation levels. First, we had previously performed a comprehensive phosphoproteomics study and we had not observed widespread alterations in phosphor-protein levels upon AZD2811 treatment [34]. Second, AZD2811 is a drug highly selective for AURKB that, at the nM concentrations and short incubation times used in our study, does not reduce phosphorylation in proteins other than AURKB substrates. This is apparent in Fig S2B, where AZD2811 treatment does not alter the phosphorylation levels of pRb (Ser780) or pEGFR (pTyr1068); in contrast with the strong decrease in phosphor-histone H3 (Ser10) which can be observed, for instance, in Fig. 7B.

Taken together, these results supported the hypothesis in Fig. 7A, demonstrating universal cleavage and activation of CASP-2 after SAC abrogation by AZD2811, followed by BID-mediated CASP-3 cleavage exclusively in cells with high BID expression.

### Amplification of Chr22q11 is a rare event in human tumors

**Fig. 8:**
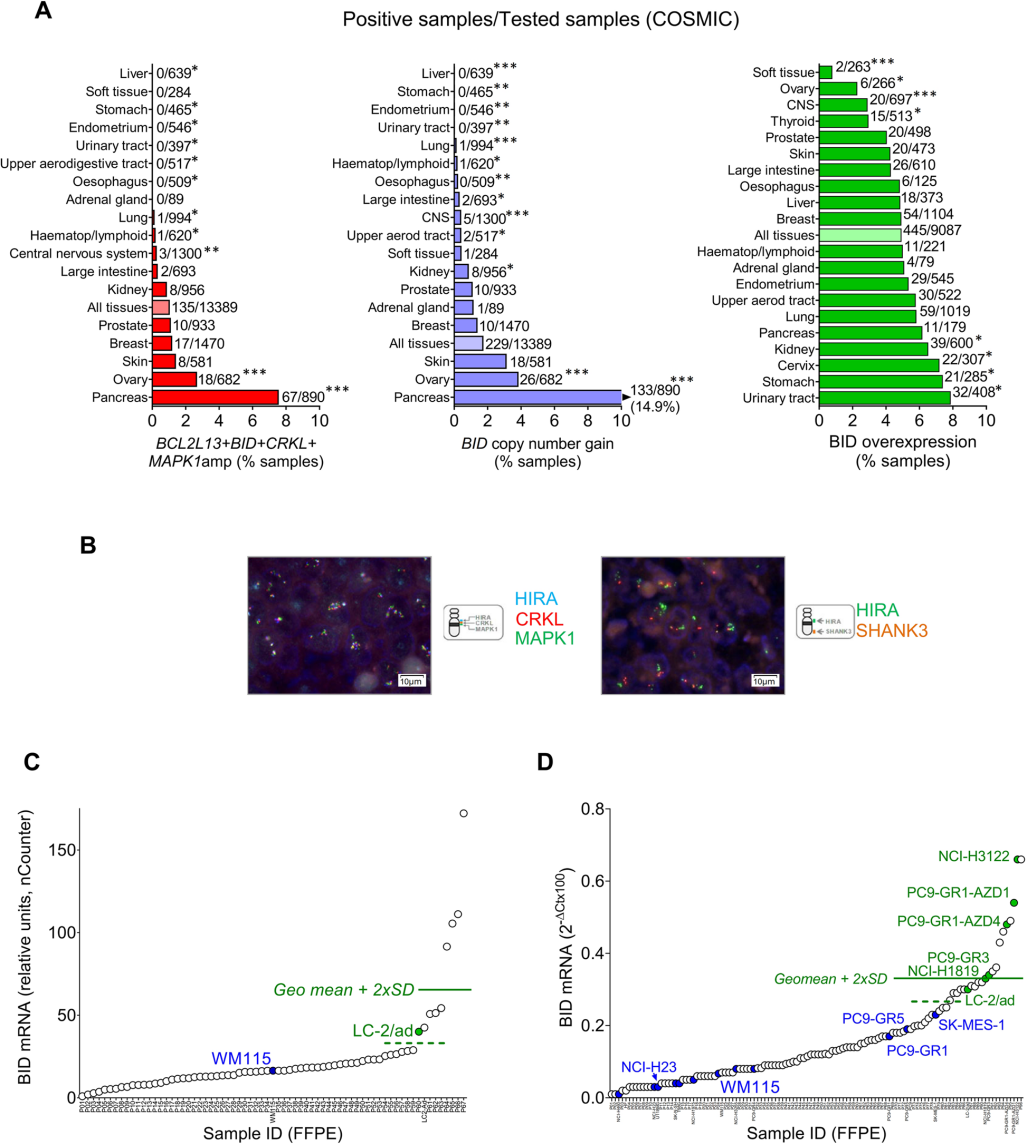
High expression of BID is frequent in human tumors while Chr22q11 amplification is a rare event. **A** Frequency of co-amplification of Chr22q11 genes (left panel, red bars), *BID* copy number gains (middle panel, blue bars) and *BID* mRNA overexpression (right panel, green bars) in human malignancies, as found in the COSMIC database. Asterisks denote significant differences with the entire tumor cohort (indicated by lighter bars) ***p<0.001, **p<0.01, *p<0.05 (two-tailed z-score). **B** FISH analysis of a tumor harboring Chr22q11 amplification. Probes for the *HIRA*, *CRKL* and *MAPK1* (upper panel) or for *HIRA* and *SHANK3* (lower panel) were used. The sample corresponded to a 54-year-old, non-smoker woman with squamous carcinoma of the lung and showed an average of 8 copies of *HIRA*, *CRKL* and *MAPK1* genes with a 3.0 ratio to the telomeric probe. **C** Levels of *BID* mRNA, as quantified by nCounter, in FFPE blocks from a cohort of 67 advanced lung tumors (white dots), AURKB/TTKi-sensitive (green dot) and AURKB/TTKi-resistant (blue dot) cell lines. The names of the cell lines are indicated. The solid and dotted lines indicate the geomean + 2xSD and + SD, respectively. **D** Levels of *BID* mRNA, as quantified by RT-Q-PCR in FFPE blocks from a cohort of 94 tumor samples (white dots, Table S14), AURKB/TTKi-sensitive (green dots) and AURKB/TTKi-resistant (blue dots) cell lines. The names of some cell lines are shown on the plot. The solid and dotted lines indicate the geomean + 2xSD and + SD, respectively, of the FFPE tumor samples

During our study, we had found BID upregulation in several cancer cell lines, accompanied by Chr22q11 amplification exclusively in one case (Table S13). Next, we decided to explore the frequency of both alterations in human tumors. In the cBioPortal database, we found a frequency of 0.5–1.2% samples with *CRKL* and *MAPK1* co-amplification with a trend to mutual exclusivity with *KRAS*, *NRAS* and *BRAF* mutations in NSCLC and melanoma (Fig. S17A-C). In the Catalogue of Somatic Mutations in Cancer (COSMIC) database; 135/13,389 (1.0%) samples showed co-amplification of *BCL2L13, BID, CRKL* and *MAPK1* and frequencies > 2% were found in ovarian or pancreatic carcinomas (Fig. 8A). The frequency of *BID* copy number gains in COSMIC samples was approximately the same as the frequency of co-amplification of *BCL2L13, BID, CRKL* and *MAPK1* in all histologies with the only exception of pancreatic cancer (Fig. 8A), indicating that copy number gains in *BID* are almost invariably associated with co-amplification of the other three Chr22q11 genes. Finally, in The Cancer Genome Atlas (TCGA), co-amplification of *BCL2L13, BID, CRKL* and *MAPK1* was present in 0.5% of reported samples (Figure S17D, E), being > 2% among sarcomas or lung squamous cell carcinomas.

Given the discrepancies among databases, we decided to analyze by Q-PCR a cohort of 143 solid tumors from patients of our institutions (Table S14). We identified three cases with copy numbers of *BID, CRKL* and *MAPK1* above the cut-off for positivity (Fig. S17F); which were submitted to FISH. One of them, a squamous carcinoma of the lung clearly showed Chr22q11 amplification (Fig. 8B) and RT-Q-PCR analysis revealed high levels of *BID* mRNA (2^−∆Ct^x100 = 0.8). Of the other two samples positive by Q-PCR, one was monosomic with 2.5 average copies of Chr22q11 (Fig. S18A) and the other showed Chr22q11 focal amplification (Fig. S18B). In contrast, 14 samples with copy number values by Q-PCR relatively high but below the cut-off, were consistently negative by FISH.

### BID upregulation is frequent across human tumors and associates with response to SAC abrogation in patient-derived xenografts

Regarding BID upregulation, COSMIC reports 445/9087 (5%) tumor samples as BID overexpressors. Frequencies in the most common malignancies range between 4 and 6%, being higher in kidney, urinary tract, stomach, and cervix tumors (Fig. 8A). We also found that, in COSMIC samples, BID upregulation significantly associates with *TP53* mutations but shows a trend to mutual exclusivity with *KRAS* or *EGFR* driver mutations, which reaches statistical significance in the case of *BRAF* (Figure S18C).

To validate the COSMIC data, we performed a prevalence study of BID upregulation in samples from our institutions. First, we re-analyzed the results previously obtained in 67 FFPE Stage IIIB-IV lung cancer samples tested with a 770-gene expression panel that includes *BID*. When using the geomean plus two times the standard deviation (geomean + 2xSD) as a cut-off value, as described [24, 25]; four tumors (6.2%) showed *BID* mRNA upregulation (Fig. 8C). Then, we prospectively tested 96 FFPE tumor samples of different histologies (Table S14) by RT-Q-PCR; together with FFPE blocks of 11 cell lines of known BID status (Table S13). Using the geomean + 2xSD as a cut-off, we found that 6/96 (6.3%) samples presented *BID* upregulation. Remarkably, this cut-off value (0.34) was very similar to the *BID* mRNA levels in the FFPE blocks of the lowest-expressing cell lines sensitive to SAC abrogation (Fig. 8D). The six tumor samples overexpressing *BID* by RT-Q-PCR were wt for *EGFR*, *NRAS*, *KRAS* and *BRAF* but three of them harbored *TP53* mutations (Table S15). In contrast, when we tested lymphocytes from non-cancer individuals and cancer patients together with FFPE biopsies from healthy lung, we found low levels of *BID* mRNA, consistently below the cut-off value mentioned above (Fig. S18D).

**Fig. 9:**
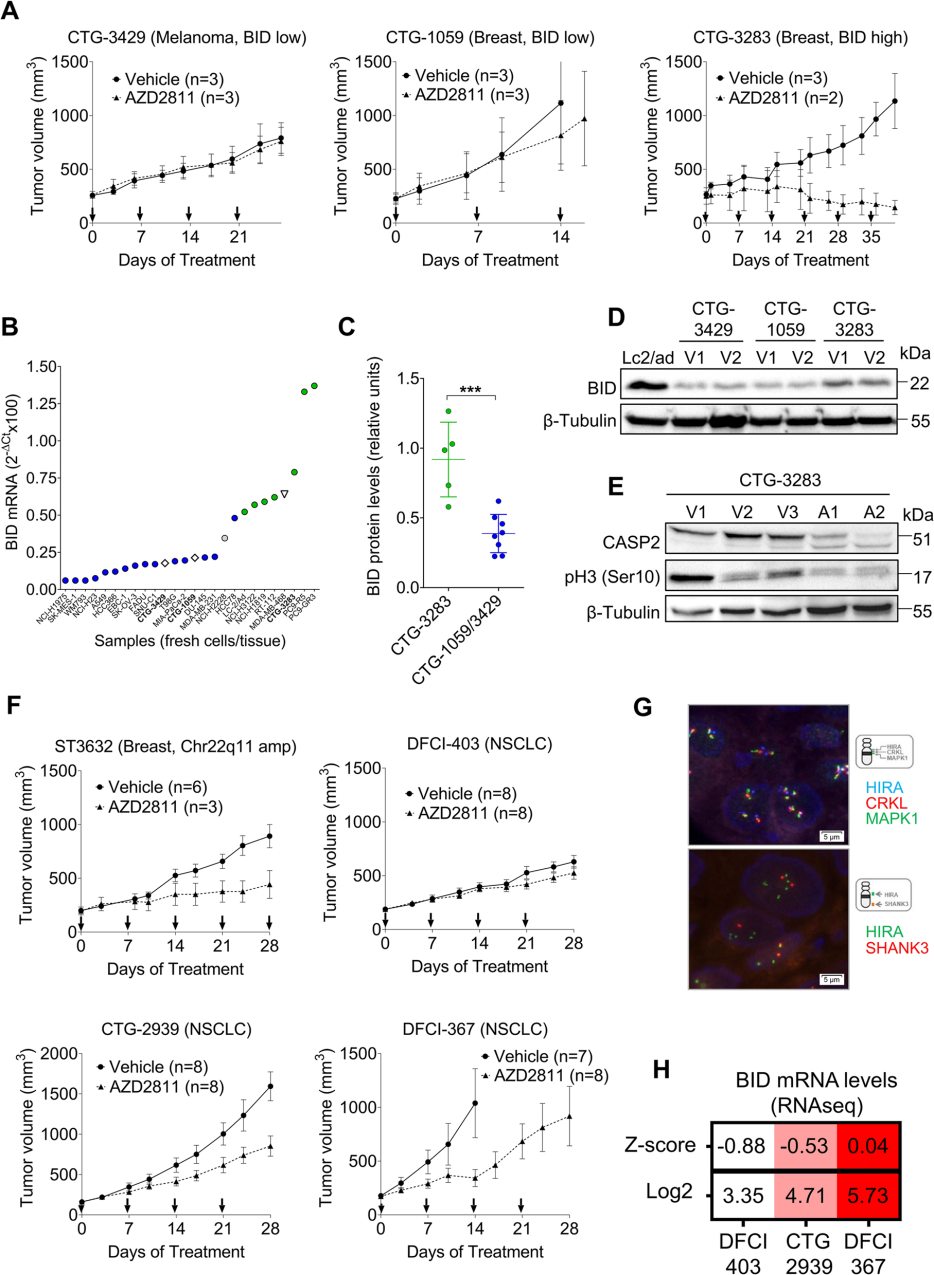
High expression of BID associates with response to SAC abrogation in patient-derived xenografts (PDXs). **A** Athymic nude mice bearing CTG-3429, CTG-1059 and CTG-3283 PDXs were treated with vehicle or an AZD2811 nanoparticle formulation (25 mg/kg) once weekly, as indicated by arrows. Panels show tumor volumes as measured by caliper (mean ± SEM). The number of mice per group is indicated in the plots. **B** Average levels of *BID* mRNA, as quantified by RT-Q-PCR, in fresh tissue of CTG-3283 (inverted triangle, *n*=5), CTG3429 and CTG-1059 (diamonds, *n*=6) patient-derived xenografts. *BID* mRNA levels in fresh cultures of cell lines sensitive (green dots) and resistant (red dots) to SAC abrogation are also plotted. **C** Quantification of the BID bands in the Western blotting analyses of PDXs (shown in Figs 9I and S18F). **D** Western blotting analysis of BID protein in vehicle-treated (V1, V2) PDXs. The results for the rest of PDXs tested are shown in Fig. S18F. **E** Western blotting analysis of CASP-2 and the AURKB product phosphor-histone H3 (pH3) in CTG-3282 patient-derived xenografts. V1-V3, xenografts treated with vehicle; A1-A2, xenografts treated with AZD2811. **F** Immunodeficient female mice bearing ST3632, DCFI-403, CTG-2939 and DCFI-367 PDXs were treated with vehicle or an AZD2811 nanoparticle formulation (25 mg/kg) once weekly, as indicated by arrows. Tumor volumes as measured by caliper are shown (mean ± SEM). The number of mice per group is indicated in the plots. **G** FISH analysis of ST3632 xenografts, revealing 4.6 copies of *HIRA*, *CRKL* and *MAPK1* per cell (upper panel) and a ratio of 2.1 of *HIRA* to the Chr22 telomeric gene *SHANK3* (lower panel). (H) *BID* mRNA levels in the NSCLC PDXs presented in (**F**), as calculated from RNA seq data. The Z-scores and log2 (value + 1) are presented

Next, we assayed the effects of SAC abrogation in a melanoma (CTG-3429) and two estrogen-receptor positive breast carcinoma (CTG-1059, CTG-3283) patient-derived xenografts (PDXs). AZD2811 nanoparticles induced regression only in the case of CTG-3283, with no effects on body weight (Fig. 9A, S18E). Fresh tumors recovered at the end of the experiment showed upregulation of BID mRNA and protein in CTG-3283 but not in CTG-1059 or CTG-3429 (Fig. 9B-D). Remarkably, *BID* mRNA levels in CTG-3283 were within the range previously observed in cell lines sensitive to SAC abrogation (Fig. 9B). Western blotting also revealed reduced CASP-2 levels associated with AZD2811 treatment in CTG-3283 (Fig. 9E, S18F). Then, we tested response to AZD2811 in four additional PDXs (Fig. 9F). One of them, an estrogen-receptor positive breast carcinoma with a Chr22q11 amplification (ST3632), as revealed by FISH, was found to be sensitive to the AZD2811 nanoparticles (Fig. 9G). The other three were NSCLC PDXs expressing different levels of *BID* mRNA, according to RNAseq data. Growth inhibition by AZD2811 nanoparticles was found to correlate with *BID* expression, with the lowest-expressing PDXs being resistant to the drug (Fig. 9F, H).

## Discussion

The antitumor activity of SAC-abrogating drugs, such as AURKBi or TTKi, varies not only among patients in clinical trials [8, 11, 35] but also in vitro; with some cell lines showing high sensitivity and others almost complete resistance. This diversity of responses, which was discovered early during the development of SAC-targeting agents [36–39], prompted the search for predictive biomarkers [40]. We had previously found that 6/18 TKI-resistant clones generated in our laboratory from *EGFR*-mut NSCLC cells were sensitive to AURKBi [19]. In this study, we used these clones to explore markers of response to SAC-abrogating drugs. First, we tested a TTKi (BAY12173899) and an AURKBi (AZD2811) in the 18 clones and discovered a perfect coincidence in response to both drugs. WES unveiled that all clones sensitive to SAC abrogation harbored Chr22q11 amplification, associated with upregulation of the corresponding genes. Amplification of Chr22q11 has been reported at low frequency in some malignancies [41, 42]; including lung cancer patients progressing to EGFR TKIs [30], although the finding was not confirmed in later studies [43]. When analyzing a cohort of solid tumors from our institutions, we also found that Chr22q11 amplification is a rare event. The cohort included 20 *EGFR*-mut biopsies at progression to TKIs (Table S14); all were negative for Chr22q11 gains.

The Chr22q11 segment contains > 350 genes, 72 were included in a silencing library that we used to transfect two *EGFR*-mut clones sensitive to SAC abrogation. Among the 72 Chr22q11 genes, only the knock-out of *BID* rendered cells resistant to AZD2811. The association of BID levels with sensitivity to SAC abrogation was then extensively validated, not only in a total of 32 *EGFR*-mut clones but also in 21 tumor cell lines of different origins. Independently of Chr22q11 status, only tumor cells with high BID levels experienced extensive cell death after SAC abrogation by AURKBi and TTKi, and cut-off values for sensitivity could be established. In addition, *BID* silencing rendered high expressing cells resistant to SAC abrogation and, conversely, ectopic expression of BID conferred sensitivity to resistant cells.

Even though both are SAC-abrogating drugs, the search for biomarkers of response to TTKi and AURKBi has been performed independently so far. Our results indicate that, at least in vitro, the sensitivity profiles of tumor cells to both types of drugs are almost identical and suggest BID upregulation as a candidate biomarker to select patients for treatment. Remarkably, analysis of databases indicates that BID overexpression is present in a significant percentage of human tumors (5–6%), a result that we validated in two cohorts from our institutions. Although there are no biomarkers in clinical use for SAC-abrogating drugs, Myc amplification and/or overexpression and *RB1* loss have been suggested as predictors of sensitivity to AURKBi, particularly in SCLC [17, 40, 44]. Regarding TTKi, mutations in *TP53* or *PTEN* were initially associated with sensitivity [45, 46], although additional studies yielded negative results [16, 39, 47] and, more recently, activating *CTNNB1* mutations have been proposed [47]. One of the limitations of our study was that it did not include any SCLC cell line. However, the 7 *EGFR*-mut clones and 3/5 cell lines sensitive to SAC abrogation were pRb-proficient and did not harbor *MYC* amplifications (Table S13). Regarding *CTNNB1*, none of the clones and cell lines included in our study carried activating mutations and the investigators proposing this biomarker also recognized that *CTNNB1* cannot be the only determinant of response to TTKi.

One of the major limitations for the clinical use of SAC inhibitors is the narrow therapeutic window they have shown in clinical trials, either as single agents [48–51] or in combination with taxanes [11, 35, 48]. The dose-limiting toxicities observed were primarily hematological, neutropenia and febrile neutropenia; with fatigue, nausea and diarrhoea being also common. Some attempts have been made to mitigate these toxic effects, at least in the case of AURKBi. Examples are the investigation of non-ATP-competitive inhibitors, which could show reduced bone marrow toxicity [52] or the development of nanoparticle formulations allowing an extended exposure profile of the drug [53, 54]. Our findings offer another potential strategy to increase the therapeutic window of SAC-targeting drugs by selecting the appropriate target population. According to the results presented here, patients with high BID expression levels could be particularly sensitive to AURKBi and TTKi, opening the possibility that they might respond to lower dosages, which reduced toxicities. However, only further exploration in clinical trials could determine the adequateness of this approach.

Our work also reveals the mechanism responsible for the association of BID upregulation with sensitivity to SAC abrogation. Although initially described as a pro-apoptotic mediator of the BH3-only family [55], BID has been subsequently recognized to be at the crossroads of several damage-response pathways [56–59]. Here, we demonstrate that BID levels regulate an AURKB/CASP-2 mitotic checkpoint that determines the fate of tumor cells when the SAC is overridden; senescence if BID expression is low but cell death in case of high BID levels (Fig. 7A). This mechanism integrates previous, disperse findings in a unified molecular framework. TTK has been described to activate AURKB through the phosphorylation of borealin during chromosome alignment [5] and, consequently, treatment of cancer cells with TTKi leads to the inhibition of AURKB, as evidenced by a decrease in phosphor-histone H3 levels [60]. AURKB, in its turn, has been reported to phosphorylate CASP-2 during normal mitosis, maintaining the caspase in an inactive state. However, in the case of cytokinesis failure, AURKB activity is reduced, leading to CASP-2 dephosphorylation and activation [28]. Multiple studies have demonstrated that SAC abrogation induces cytokinesis failure, aneuploidy/polyploidy and cell cycle arrest [5, 6, 16, 60–63]. The subsequent generation of extra centrosomes triggers the association of PIDD1, CRADD and pro-CASP2 to form the so-called PIDDosome, which activates CASP-2 by dimerization followed by autoproteolytic cleavage [64, 65]. In addition, it has been shown that PIDDosome-activated CASP-2 can either lead to cell death through BID cleavage and CASP-3 activation or to cell cycle arrest and survival through MDM2/p53/p21 dependent or independent pathways [29, 66–69]. Interestingly, it has also been recognized that PIDDosome-based activation of CASP-2 is not necessarily toxic and that a second signal is required for CASP-2 mediated apoptosis [29]. Finally, BID was identified as one of the top 15 candidate genes associated with sensitivity to TTKi in a CRISPR/Cas9 screening of triple negative breast cancer cells, although it was not further investigated [15] and, in a study published when this manuscript was in preparation, Aurora inhibitors combined with BH3-mimetics were shown to induce apoptosis through a pathway involving select BH3-only proteins and CASP-2 [70].

We observed CASP-2 cleavage after SAC abrogation in all cell lines and clones tested; independently of cell fate. However, cleavage of CASP-3 followed by apoptosis was strictly dependent on BID upregulation. In addition, silencing of *PIDD1*, *CRADD* or *CASP-2* abolished BID and CASP-3 activation and sensitivity to AURKBi and TTKi. Taken together, these findings indicate that inhibition of AURKB triggers PIDDosome-mediated CASP-2 activation and identify BID upregulation as the “second signal” mentioned above, demonstrating that high levels of BID switch cells from cycle arrest to cell death after CASP-2 cleavage (Fig. 7A). Although low levels of truncated BID seem to be sufficient to trigger cytochrome c release [71], CASP-2 has been described to be relatively inefficient at catalyzing BID cleavage [72]. Our results prove that BID concentration is indeed the limiting factor in CASP-2 induced apoptosis, resulting in a “saturation” kinetics where BID acts as the substrate and cell death as the ultimate “product” of the enzymatic reaction catalyzed by CASP-2.

In our study, we focused on the pathway leading to cell death after the AURKB/CASP-2 checkpoint. Further research is needed to characterize the cell cycle arrest/senescence pathway; in particular, if it is triggered by the MDM2/p53/p21 cascade. It has been demonstrated that active CASP-2 can cleave MDM2, resulting in p53 stabilization and cell cycle arrest [73, 74], but p53-independent CASP-2 activities have been also identified [66, 75]. Contradictory evidence also exists in the case of senescence after SAC abrogation, which has been described to be mediated by ATM/Chk2 in some cells [76] but p53-p21 in others [77, 78]. In our panel of cell lines, no differences were apparent in response to SAC-targeting agents between p53 wild-type and p53-null cell lines (Table S13), suggesting p53-independence at least in the null cases.

## Conclusion

We have demonstrated that an AURKB/CASP-2 mechanism, regulated by the levels of BID, determines the fate of tumor cells after abrogation of the spindle assembly checkpoint by AURKB or TTK inhibitors (Fig. 7A). If BID is upregulated, CASP-3 and apoptosis are triggered; but if BID expression is low, the tumor cell survives and enters senescence. Our results pave the way for the clinical exploration of drugs targeting SAC using BID overexpression, which appears in 6% of human tumors, as a biomarker for patient selection.

## Supplementary Information

**Additional file 1.**

## Data Availability

Raw NGS and WES data of cell lines and clones related to Fig. 2A-S5 and Table S5 are deposited in the Sequence Read Archive (SRA) of the National Center for Biotechnology Information (NCBI), under the accession codes PRJNA524804 (project) and SAMN11035311-SAMN11035324. The raw numerical data corresponding to the plots presented in the figures and supplementary figures, the sequences of the 253 sgRNAs, shRNAs and plasmids used for gene knock-out and ectopic expression experiments, and the sequences of the 96 nCounter probes of the custom panel employed for expression analysis are available upon reasonable request from the corresponding authors. All other data supporting the findings of this study are presented within the article and its Supplementary files. Requests for materials should also be addressed to the corresponding authors. AZD2811 nanoparticle formulation could not be made available due to restriction of license agreements with Pfizer for nanoparticle technology.
