# Supplementary material for: BID expression determines the apoptotic fate of cancer cells after abrogation of the spindle assembly checkpoint by AURKB or TTK inhibitors
Source: Mol Cancer. 2023 Jul 13;22:110. doi: 10.1186/s12943-023-01815-w (PMC10339641; doi:10.1186/s12943-023-01815-w)
Supplement: Supplementary file 1 — Additional file 1. [file 12943_2023_1815_MOESM1_ESM.zip › Figure Supplementary_rev2.pptx]

## Slide 1
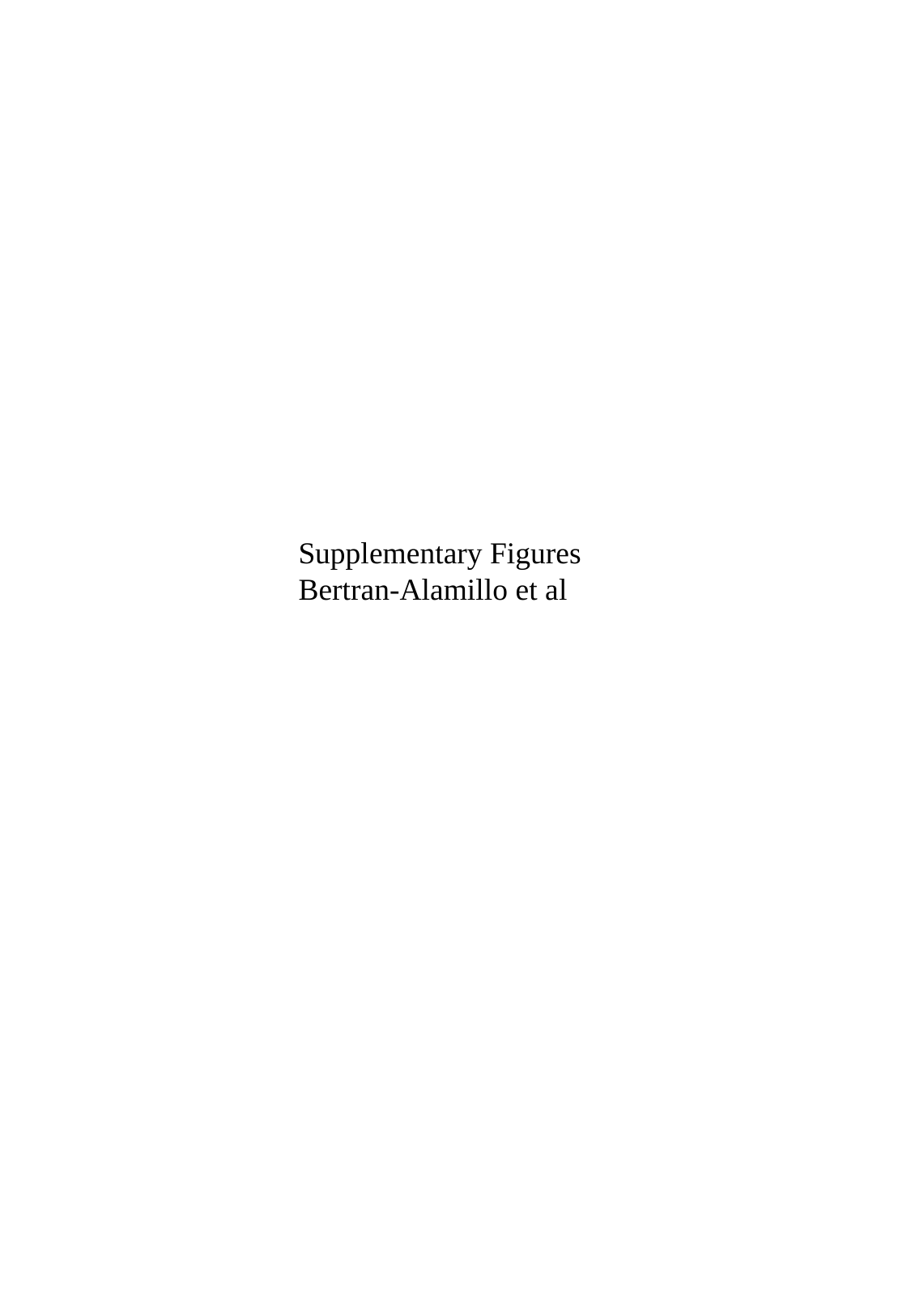

Supplementary Figures
Bertran-Alamillo et al

## Slide 2
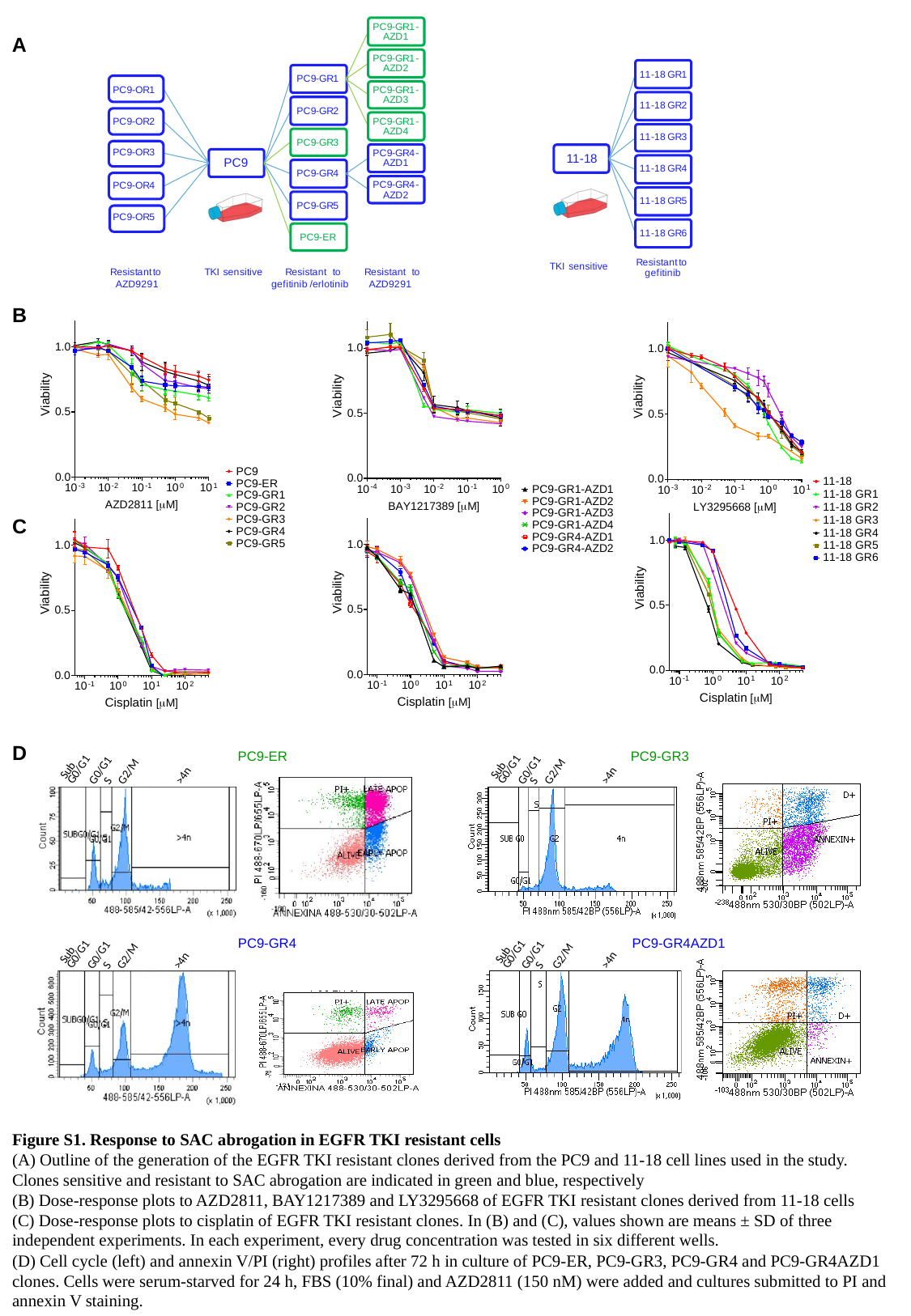

A
B
C
D
PC9-ER
PC9-GR3
Sub G0/G1
Sub G0/G1
G0/G1
S
G2/M
>4n
G0/G1
S
G2/M
>4n
PC9-GR4
PC9-GR4AZD1
Sub G0/G1
Sub G0/G1
G0/G1
S
G2/M
>4n
G0/G1
S
G2/M
>4n
Figure S1. Response to SAC abrogation in EGFR TKI resistant cells
(A) Outline of the generation of the EGFR TKI resistant clones derived from the PC9 and 11-18 cell lines used in the study. Clones sensitive and resistant to SAC abrogation are indicated in green and blue, respectively
(B) Dose-response plots to AZD2811, BAY1217389 and LY3295668 of EGFR TKI resistant clones derived from 11-18 cells
(C) Dose-response plots to cisplatin of EGFR TKI resistant clones. In (B) and (C), values shown are means ± SD of three independent experiments. In each experiment, every drug concentration was tested in six different wells.
(D) Cell cycle (left) and annexin V/PI (right) profiles after 72 h in culture of PC9-ER, PC9-GR3, PC9-GR4 and PC9-GR4AZD1 clones. Cells were serum-starved for 24 h, FBS (10% final) and AZD2811 (150 nM) were added and cultures submitted to PI and annexin V staining.

## Slide 3
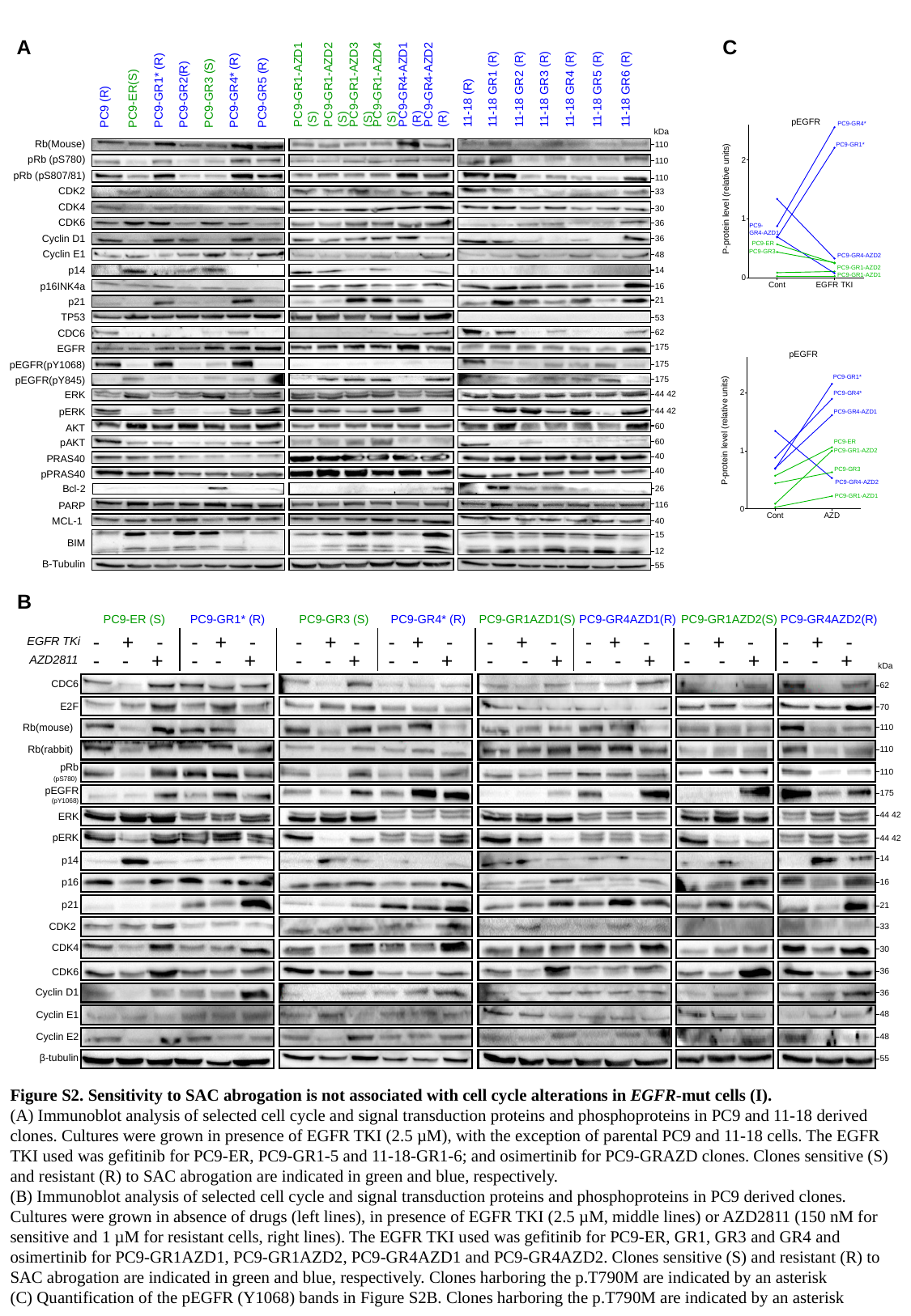

PC9-GR1-AZD1 (S)
PC9-GR1-AZD2 (S)
PC9-GR1-AZD3 (S)
PC9-GR1-AZD4 (S)
PC9-GR4-AZD1 (R)
PC9-GR4-AZD2 (R)
11-18 (R)
11-18 GR1 (R)
11-18 GR2 (R)
11-18 GR3 (R)
11-18 GR4 (R)
11-18 GR5 (R)
11-18 GR6 (R)
PC9 (R)
PC9-GR1* (R)
PC9-GR4* (R)
PC9-GR5 (R)
PC9-ER(S)
PC9-GR2(R)
PC9-GR3 (S)
A
C
kDa
 Rb(Mouse)
110
pRb (pS780)
110
pRb (pS807/81)
110
CDK2
33
CDK4
30
CDK6
36
Cyclin D1
36
Cyclin E1
48
p14
14
p16INK4a
16
21
p21
TP53
53
CDC6
62
175
EGFR
pEGFR(pY1068)
175
175
pEGFR(pY845)
ERK
44 42
44 42
pERK
60
AKT
60
pAKT
40
PRAS40
40
pPRAS40
Bcl-2
26
PARP
116
MCL-1
40
15
BIM
12
Β-Tubulin
55
B
PC9-ER (S)
PC9-GR1* (R)
PC9-GR3 (S)
PC9-GR4* (R)
PC9-GR1AZD1(S)
PC9-GR4AZD1(R)
PC9-GR1AZD2(S)
PC9-GR4AZD2(R)
- + - - + - - + - - + - - + - - + - - + - - + -
EGFR TKi
- - + - - + - - + - - + - - + - - + - - + - - +
AZD2811
kDa
CDC6
62
E2F
70
Rb(mouse)
110
Rb(rabbit)
110
pRb
 (pS780)
110
pEGFR
(pY1068)
175
44 42
ERK
pERK
44 42
14
p14
p16
16
p21
21
CDK2
33
CDK4
30
CDK6
36
Cyclin D1
36
48
Cyclin E1
Cyclin E2
48
β-tubulin
55
Figure S2. Sensitivity to SAC abrogation is not associated with cell cycle alterations in EGFR-mut cells (I).
(A) Immunoblot analysis of selected cell cycle and signal transduction proteins and phosphoproteins in PC9 and 11-18 derived clones. Cultures were grown in presence of EGFR TKI (2.5 µM), with the exception of parental PC9 and 11-18 cells. The EGFR TKI used was gefitinib for PC9-ER, PC9-GR1-5 and 11-18-GR1-6; and osimertinib for PC9-GRAZD clones. Clones sensitive (S) and resistant (R) to SAC abrogation are indicated in green and blue, respectively.
(B) Immunoblot analysis of selected cell cycle and signal transduction proteins and phosphoproteins in PC9 derived clones. Cultures were grown in absence of drugs (left lines), in presence of EGFR TKI (2.5 µM, middle lines) or AZD2811 (150 nM for sensitive and 1 µM for resistant cells, right lines). The EGFR TKI used was gefitinib for PC9-ER, GR1, GR3 and GR4 and osimertinib for PC9-GR1AZD1, PC9-GR1AZD2, PC9-GR4AZD1 and PC9-GR4AZD2. Clones sensitive (S) and resistant (R) to SAC abrogation are indicated in green and blue, respectively. Clones harboring the p.T790M are indicated by an asterisk
(C) Quantification of the pEGFR (Y1068) bands in Figure S2B. Clones harboring the p.T790M are indicated by an asterisk

## Slide 4
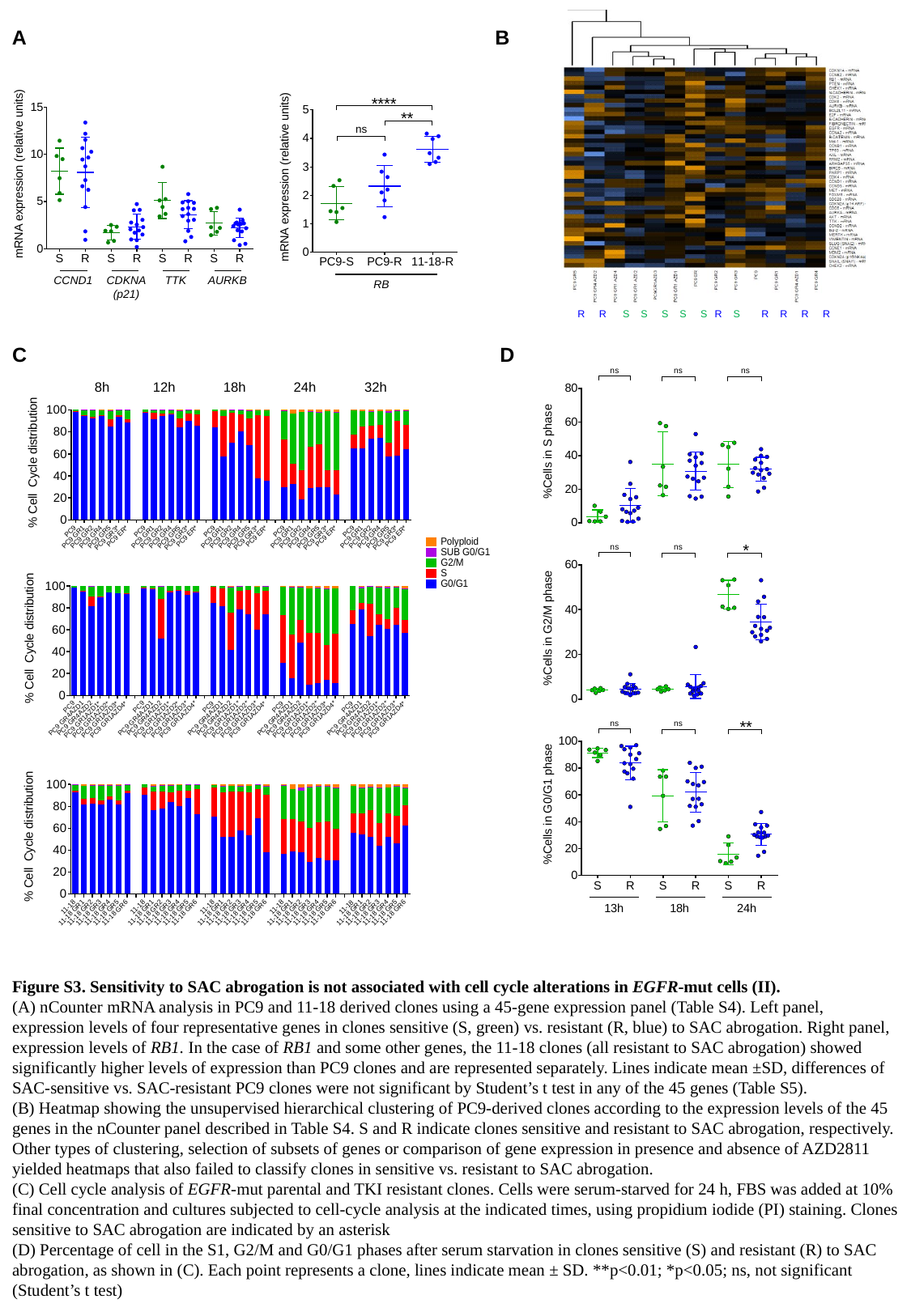

A
B
R R
S S S S S
R
S
R R R R
C
D
8h
12h
18h
24h
32h
Figure S3. Sensitivity to SAC abrogation is not associated with cell cycle alterations in EGFR-mut cells (II).
(A) nCounter mRNA analysis in PC9 and 11-18 derived clones using a 45-gene expression panel (Table S4). Left panel, expression levels of four representative genes in clones sensitive (S, green) vs. resistant (R, blue) to SAC abrogation. Right panel, expression levels of RB1. In the case of RB1 and some other genes, the 11-18 clones (all resistant to SAC abrogation) showed significantly higher levels of expression than PC9 clones and are represented separately. Lines indicate mean ±SD, differences of SAC-sensitive vs. SAC-resistant PC9 clones were not significant by Student’s t test in any of the 45 genes (Table S5).
(B) Heatmap showing the unsupervised hierarchical clustering of PC9-derived clones according to the expression levels of the 45 genes in the nCounter panel described in Table S4. S and R indicate clones sensitive and resistant to SAC abrogation, respectively. Other types of clustering, selection of subsets of genes or comparison of gene expression in presence and absence of AZD2811 yielded heatmaps that also failed to classify clones in sensitive vs. resistant to SAC abrogation.
(C) Cell cycle analysis of EGFR-mut parental and TKI resistant clones. Cells were serum-starved for 24 h, FBS was added at 10% final concentration and cultures subjected to cell-cycle analysis at the indicated times, using propidium iodide (PI) staining. Clones sensitive to SAC abrogation are indicated by an asterisk
(D) Percentage of cell in the S1, G2/M and G0/G1 phases after serum starvation in clones sensitive (S) and resistant (R) to SAC abrogation, as shown in (C). Each point represents a clone, lines indicate mean ± SD. **p<0.01; *p<0.05; ns, not significant (Student’s t test)

## Slide 5
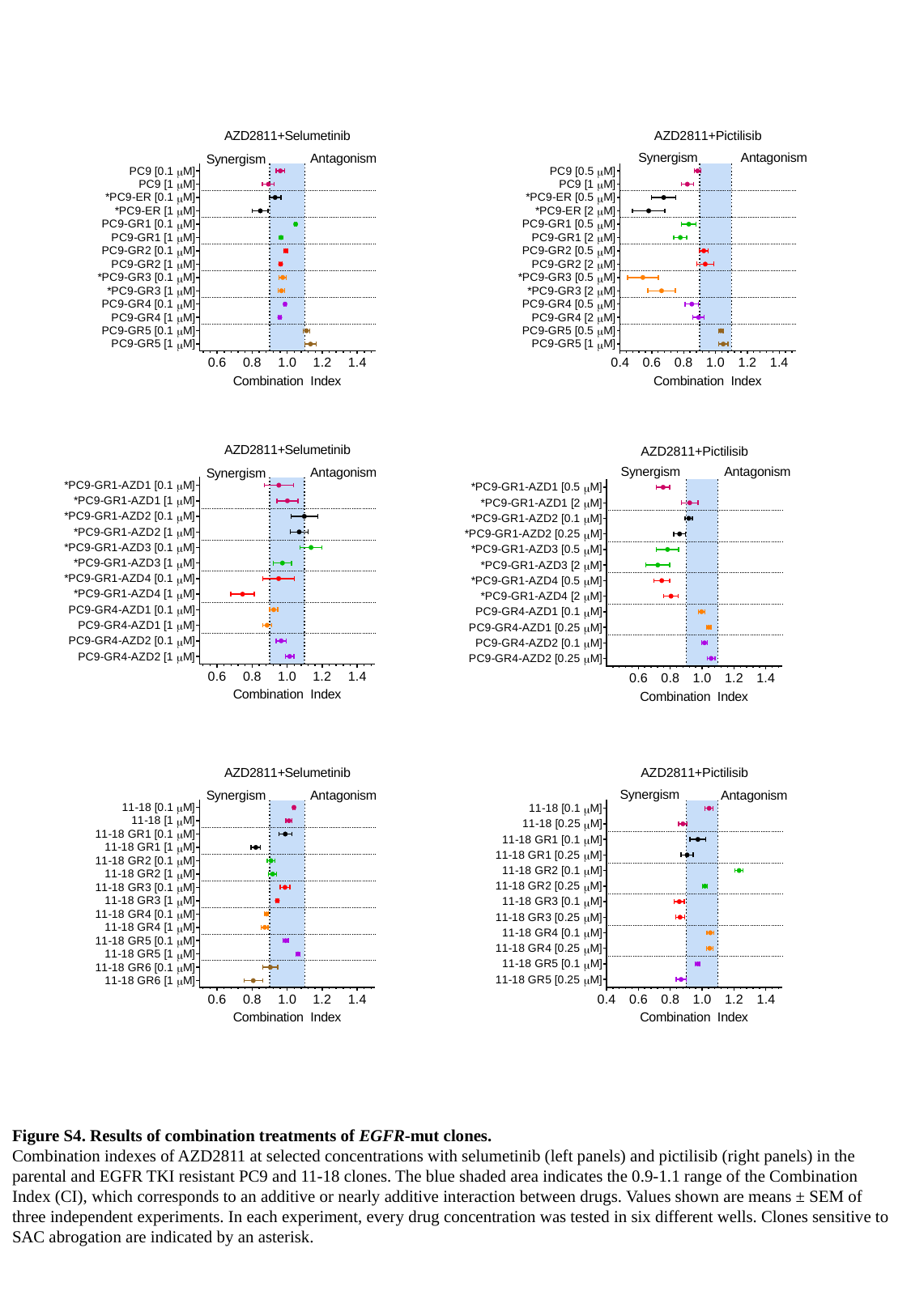

Figure S4. Results of combination treatments of EGFR-mut clones.
Combination indexes of AZD2811 at selected concentrations with selumetinib (left panels) and pictilisib (right panels) in the parental and EGFR TKI resistant PC9 and 11-18 clones. The blue shaded area indicates the 0.9-1.1 range of the Combination Index (CI), which corresponds to an additive or nearly additive interaction between drugs. Values shown are means ± SEM of three independent experiments. In each experiment, every drug concentration was tested in six different wells. Clones sensitive to SAC abrogation are indicated by an asterisk.

## Slide 6
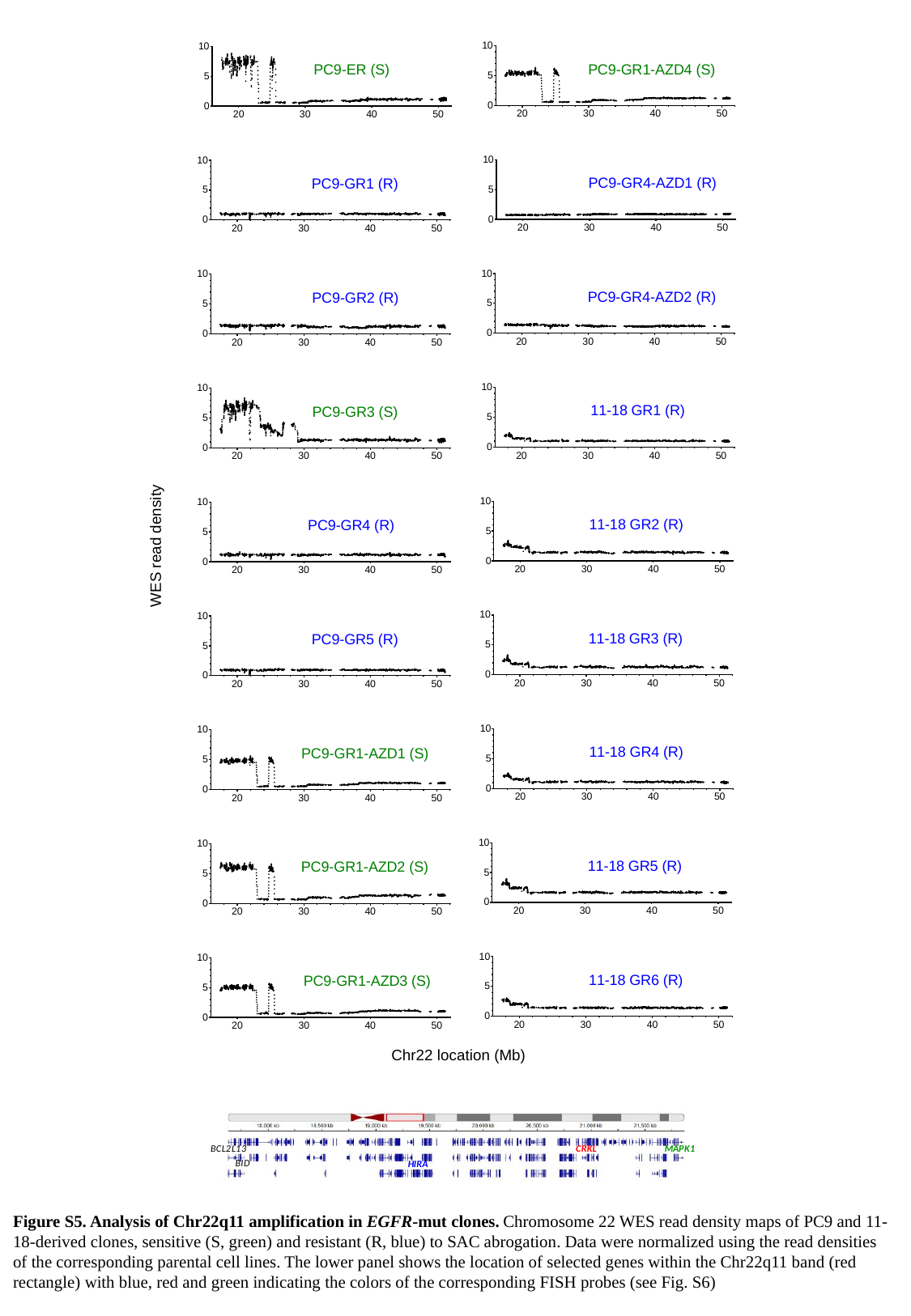

WES read density
Chr22 location (Mb)
Figure S5. Analysis of Chr22q11 amplification in EGFR-mut clones. Chromosome 22 WES read density maps of PC9 and 11-18-derived clones, sensitive (S, green) and resistant (R, blue) to SAC abrogation. Data were normalized using the read densities of the corresponding parental cell lines. The lower panel shows the location of selected genes within the Chr22q11 band (red rectangle) with blue, red and green indicating the colors of the corresponding FISH probes (see Fig. S6)

## Slide 7
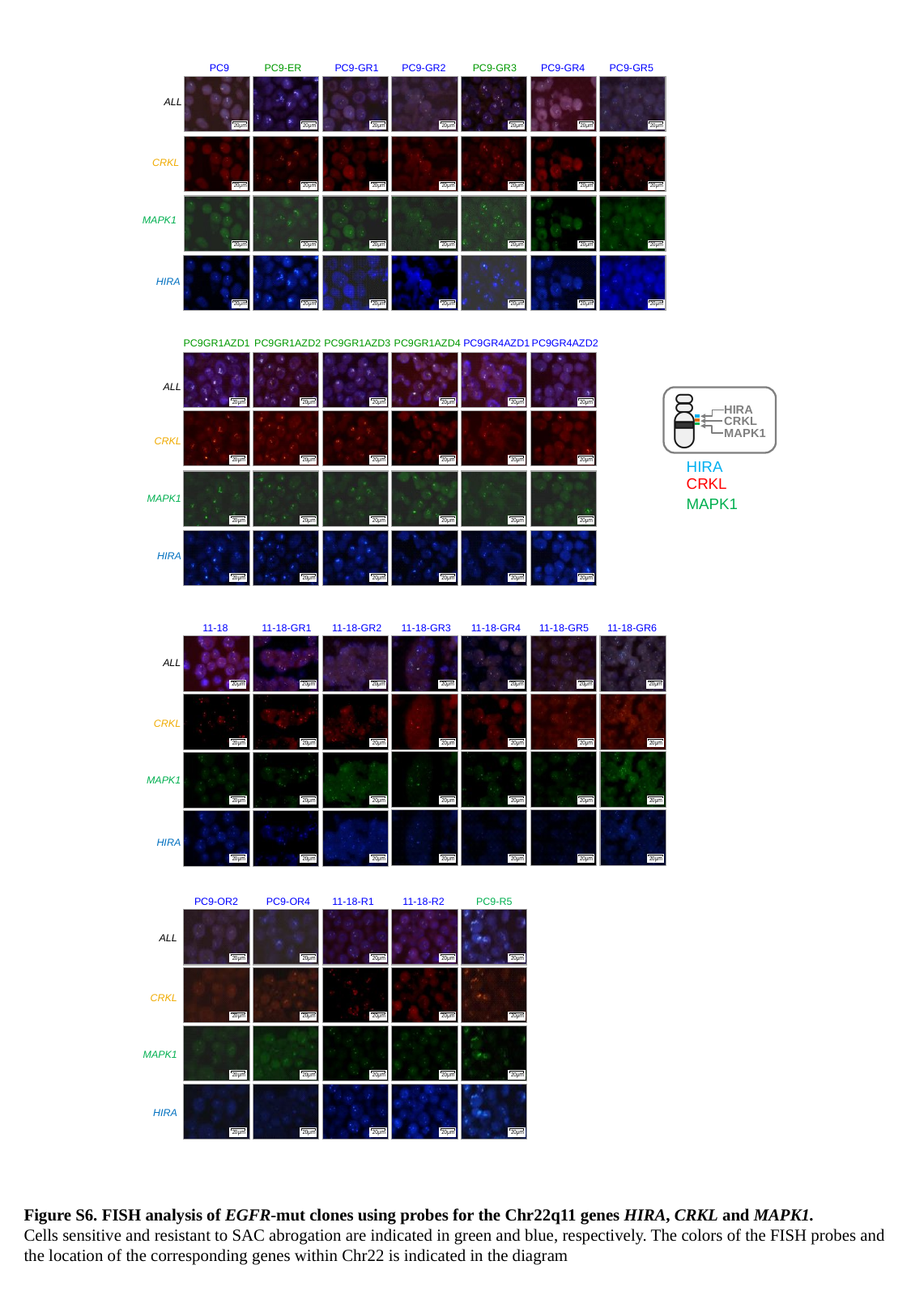

PC9
PC9-ER
PC9-GR1
PC9-GR2
PC9-GR3
PC9-GR4
PC9-GR5
ALL
CRKL
MAPK1
HIRA
PC9GR1AZD1
PC9GR1AZD2
PC9GR1AZD3
PC9GR1AZD4
PC9GR4AZD1
PC9GR4AZD2
ALL
CRKL
HIRA
CRKL
MAPK1
MAPK1
HIRA
11-18
11-18-GR1
11-18-GR2
11-18-GR3
11-18-GR4
11-18-GR5
11-18-GR6
ALL
CRKL
MAPK1
HIRA
PC9-OR2
PC9-OR4
11-18-R1
11-18-R2
PC9-R5
ALL
CRKL
MAPK1
HIRA
Figure S6. FISH analysis of EGFR-mut clones using probes for the Chr22q11 genes HIRA, CRKL and MAPK1.
Cells sensitive and resistant to SAC abrogation are indicated in green and blue, respectively. The colors of the FISH probes and the location of the corresponding genes within Chr22 is indicated in the diagram

## Slide 8
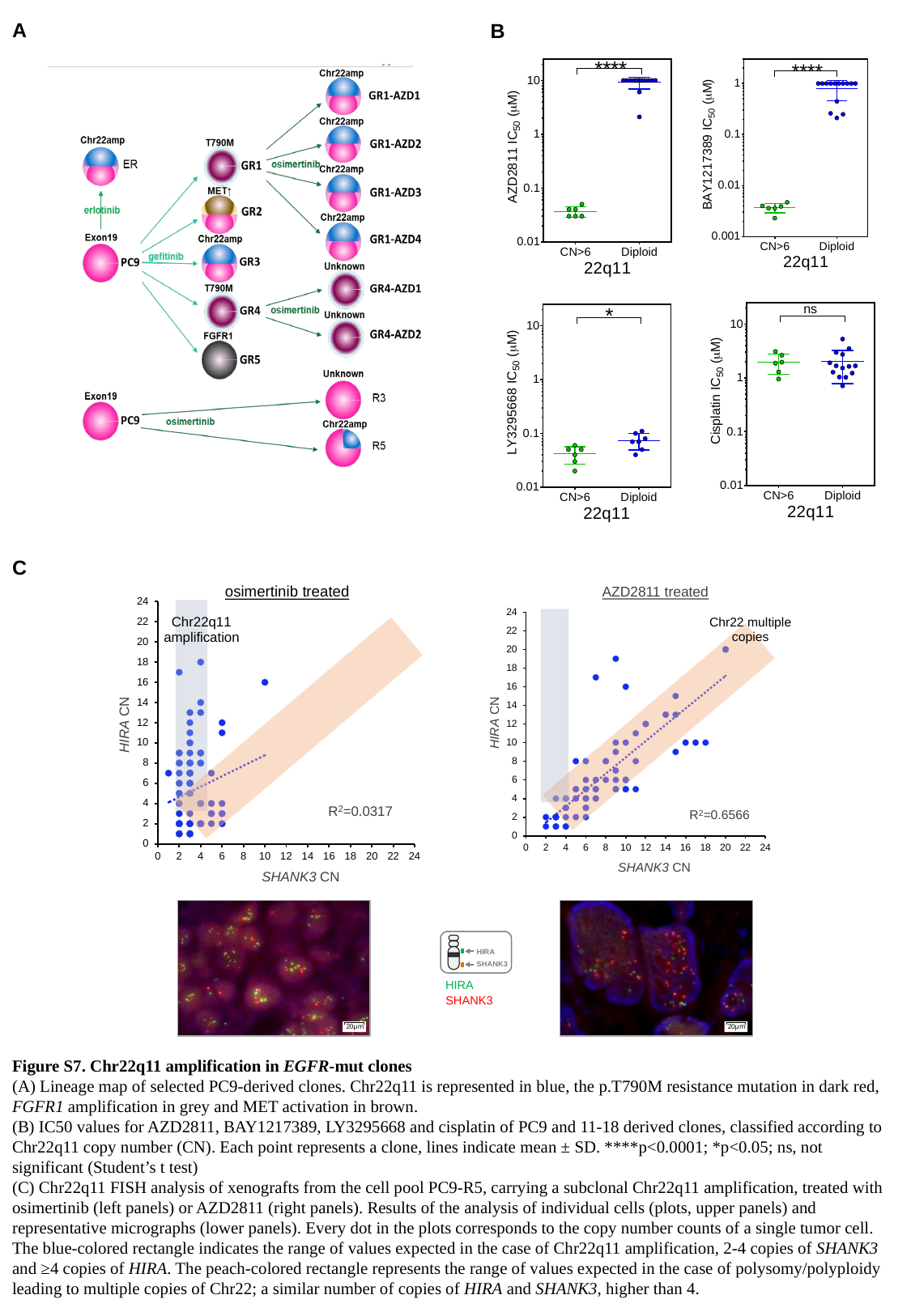

A
B
C
HIRA
SHANK3
 20μm
 20μm
Figure S7. Chr22q11 amplification in EGFR-mut clones
(A) Lineage map of selected PC9-derived clones. Chr22q11 is represented in blue, the p.T790M resistance mutation in dark red, FGFR1 amplification in grey and MET activation in brown.
(B) IC50 values for AZD2811, BAY1217389, LY3295668 and cisplatin of PC9 and 11-18 derived clones, classified according to Chr22q11 copy number (CN). Each point represents a clone, lines indicate mean ± SD. ****p<0.0001; *p<0.05; ns, not significant (Student’s t test)
(C) Chr22q11 FISH analysis of xenografts from the cell pool PC9-R5, carrying a subclonal Chr22q11 amplification, treated with osimertinib (left panels) or AZD2811 (right panels). Results of the analysis of individual cells (plots, upper panels) and representative micrographs (lower panels). Every dot in the plots corresponds to the copy number counts of a single tumor cell. The blue-colored rectangle indicates the range of values expected in the case of Chr22q11 amplification, 2-4 copies of SHANK3 and ≥4 copies of HIRA. The peach-colored rectangle represents the range of values expected in the case of polysomy/polyploidy leading to multiple copies of Chr22; a similar number of copies of HIRA and SHANK3, higher than 4.

## Slide 9
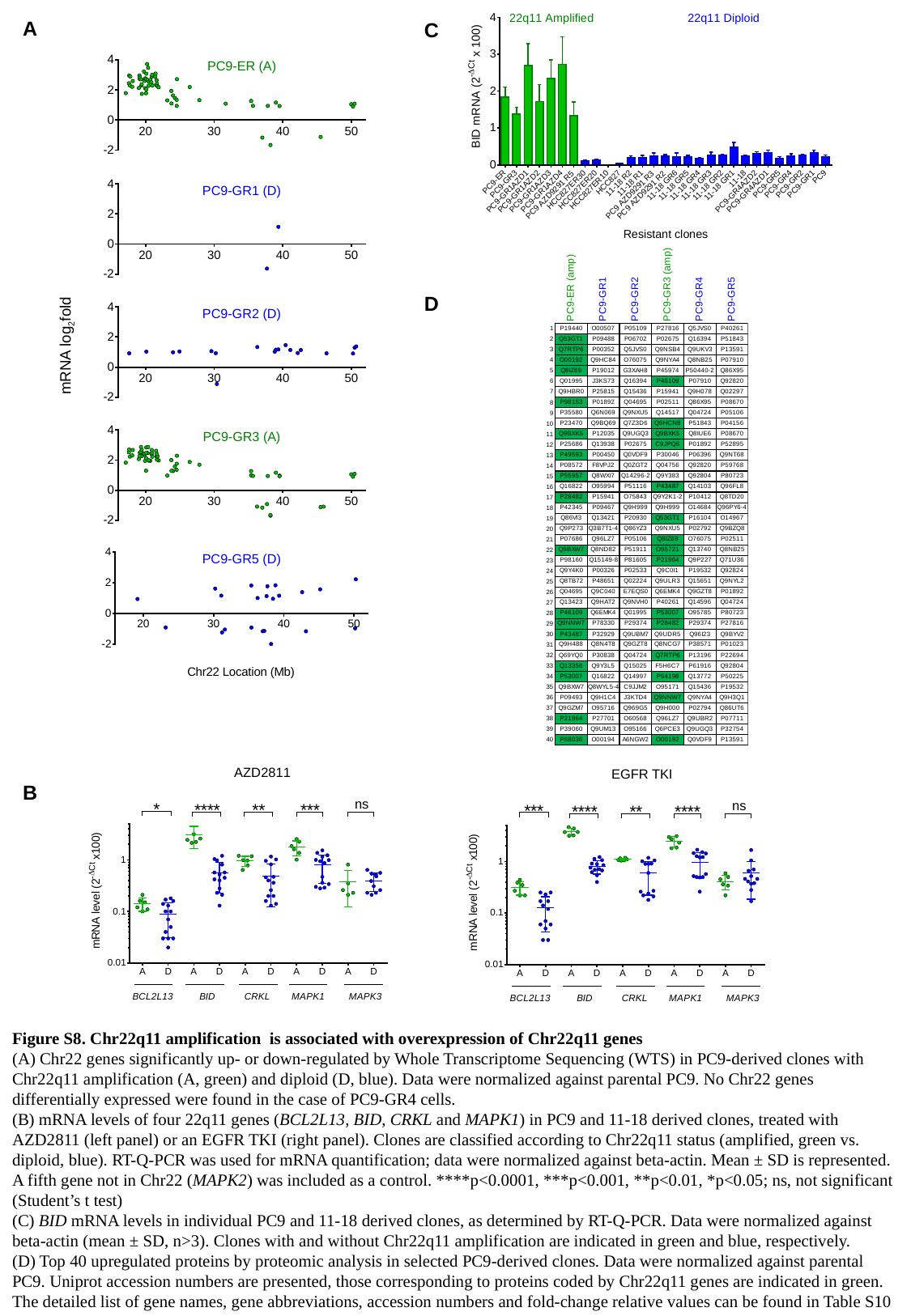

A
C
D
mRNA log2fold
B
Figure S8. Chr22q11 amplification is associated with overexpression of Chr22q11 genes
(A) Chr22 genes significantly up- or down-regulated by Whole Transcriptome Sequencing (WTS) in PC9-derived clones with Chr22q11 amplification (A, green) and diploid (D, blue). Data were normalized against parental PC9. No Chr22 genes differentially expressed were found in the case of PC9-GR4 cells.
(B) mRNA levels of four 22q11 genes (BCL2L13, BID, CRKL and MAPK1) in PC9 and 11-18 derived clones, treated with AZD2811 (left panel) or an EGFR TKI (right panel). Clones are classified according to Chr22q11 status (amplified, green vs. diploid, blue). RT-Q-PCR was used for mRNA quantification; data were normalized against beta-actin. Mean ± SD is represented. A fifth gene not in Chr22 (MAPK2) was included as a control. ****p<0.0001, ***p<0.001, **p<0.01, *p<0.05; ns, not significant (Student’s t test)
(C) BID mRNA levels in individual PC9 and 11-18 derived clones, as determined by RT-Q-PCR. Data were normalized against beta-actin (mean ± SD, n>3). Clones with and without Chr22q11 amplification are indicated in green and blue, respectively.
(D) Top 40 upregulated proteins by proteomic analysis in selected PC9-derived clones. Data were normalized against parental PC9. Uniprot accession numbers are presented, those corresponding to proteins coded by Chr22q11 genes are indicated in green. The detailed list of gene names, gene abbreviations, accession numbers and fold-change relative values can be found in Table S10

## Slide 10
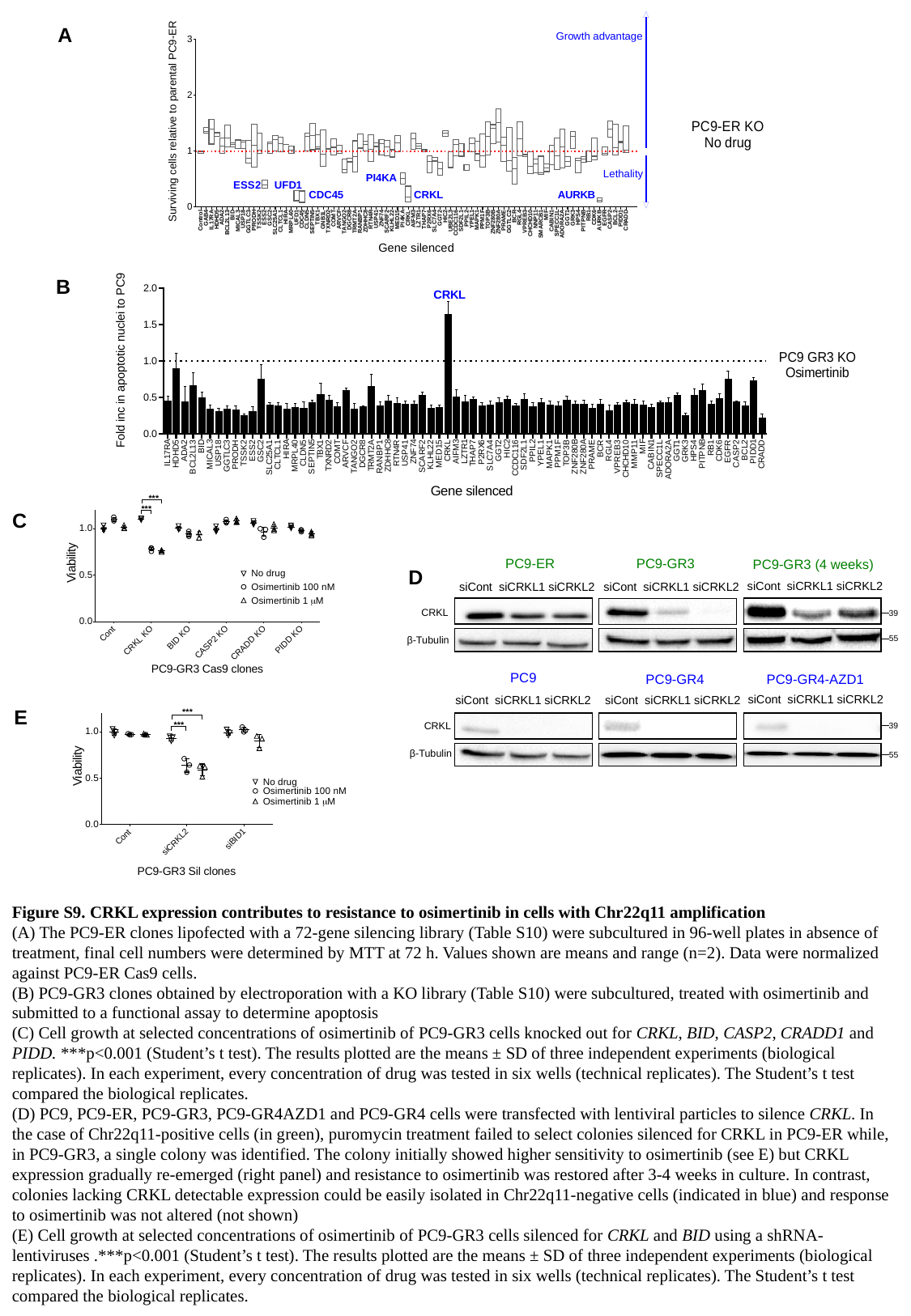

A
B
C
PC9-GR3
PC9-ER
PC9-GR3 (4 weeks)
D
siCont siCRKL1 siCRKL2
siCont siCRKL1 siCRKL2
siCont siCRKL1 siCRKL2
CRKL
39
55
β-Tubulin
PC9
PC9-GR4
PC9-GR4-AZD1
siCont siCRKL1 siCRKL2
siCont siCRKL1 siCRKL2
siCont siCRKL1 siCRKL2
E
CRKL
39
β-Tubulin
55
Figure S9. CRKL expression contributes to resistance to osimertinib in cells with Chr22q11 amplification
(A) The PC9-ER clones lipofected with a 72-gene silencing library (Table S10) were subcultured in 96-well plates in absence of treatment, final cell numbers were determined by MTT at 72 h. Values shown are means and range (n=2). Data were normalized against PC9-ER Cas9 cells.
(B) PC9-GR3 clones obtained by electroporation with a KO library (Table S10) were subcultured, treated with osimertinib and submitted to a functional assay to determine apoptosis
(C) Cell growth at selected concentrations of osimertinib of PC9-GR3 cells knocked out for CRKL, BID, CASP2, CRADD1 and PIDD. ***p<0.001 (Student’s t test). The results plotted are the means ± SD of three independent experiments (biological replicates). In each experiment, every concentration of drug was tested in six wells (technical replicates). The Student’s t test compared the biological replicates.
(D) PC9, PC9-ER, PC9-GR3, PC9-GR4AZD1 and PC9-GR4 cells were transfected with lentiviral particles to silence CRKL. In the case of Chr22q11-positive cells (in green), puromycin treatment failed to select colonies silenced for CRKL in PC9-ER while, in PC9-GR3, a single colony was identified. The colony initially showed higher sensitivity to osimertinib (see E) but CRKL expression gradually re-emerged (right panel) and resistance to osimertinib was restored after 3-4 weeks in culture. In contrast, colonies lacking CRKL detectable expression could be easily isolated in Chr22q11-negative cells (indicated in blue) and response to osimertinib was not altered (not shown)
(E) Cell growth at selected concentrations of osimertinib of PC9-GR3 cells silenced for CRKL and BID using a shRNA-lentiviruses .***p<0.001 (Student’s t test). The results plotted are the means ± SD of three independent experiments (biological replicates). In each experiment, every concentration of drug was tested in six wells (technical replicates). The Student’s t test compared the biological replicates.

## Slide 11
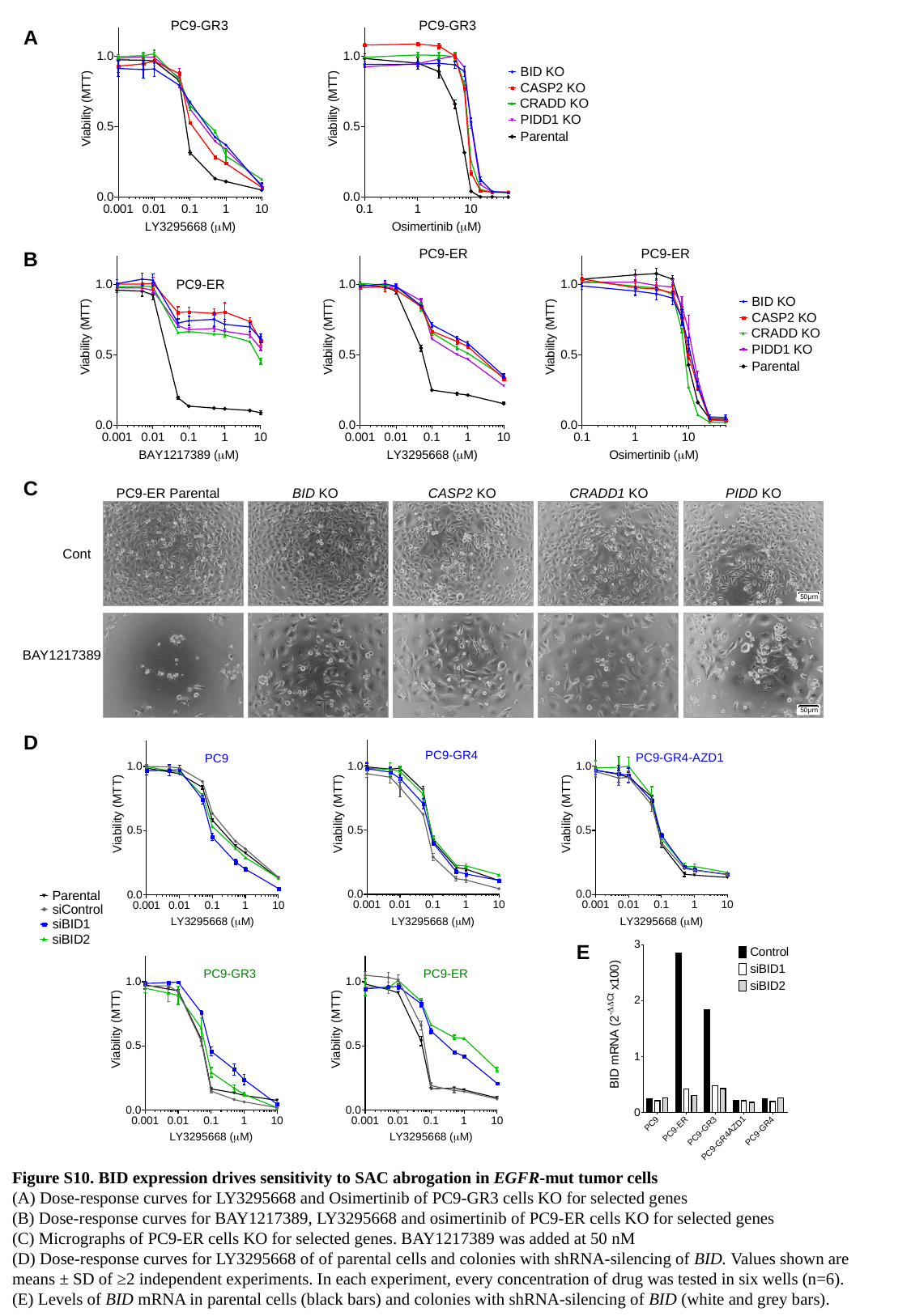

A
B
C
PC9-ER Parental
BID KO
CASP2 KO
CRADD1 KO
PIDD KO
Cont
 50μm
BAY1217389
 50μm
D
E
Figure S10. BID expression drives sensitivity to SAC abrogation in EGFR-mut tumor cells
(A) Dose-response curves for LY3295668 and Osimertinib of PC9-GR3 cells KO for selected genes
(B) Dose-response curves for BAY1217389, LY3295668 and osimertinib of PC9-ER cells KO for selected genes
(C) Micrographs of PC9-ER cells KO for selected genes. BAY1217389 was added at 50 nM
(D) Dose-response curves for LY3295668 of of parental cells and colonies with shRNA-silencing of BID. Values shown are means ± SD of ≥2 independent experiments. In each experiment, every concentration of drug was tested in six wells (n=6).
(E) Levels of BID mRNA in parental cells (black bars) and colonies with shRNA-silencing of BID (white and grey bars).

## Slide 12
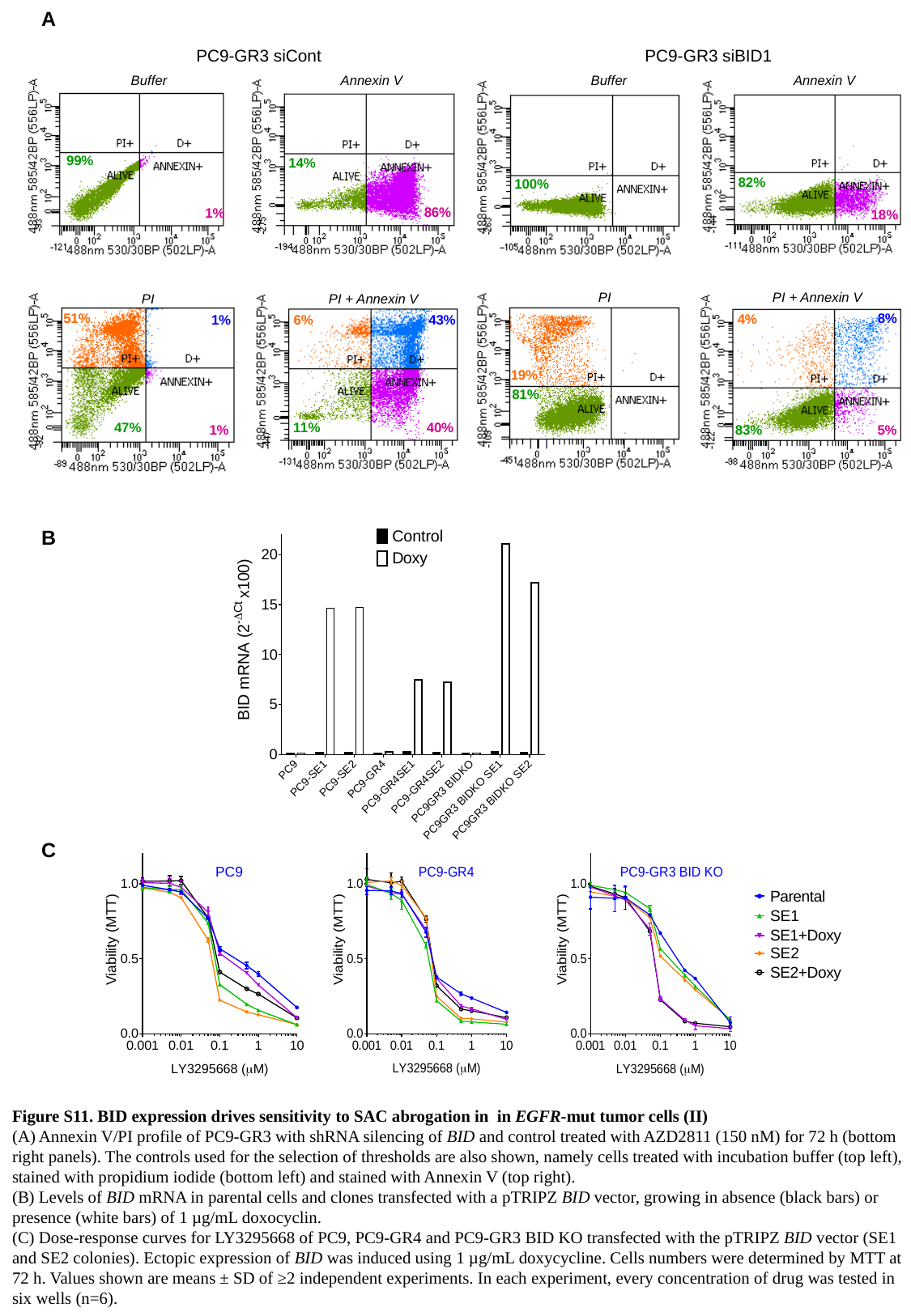

A
PC9-GR3 siCont
PC9-GR3 siBID1
Buffer
Annexin V
Buffer
Annexin V
PI
PI + Annexin V
PI
PI + Annexin V
B
C
Figure S11. BID expression drives sensitivity to SAC abrogation in in EGFR-mut tumor cells (II)
(A) Annexin V/PI profile of PC9-GR3 with shRNA silencing of BID and control treated with AZD2811 (150 nM) for 72 h (bottom right panels). The controls used for the selection of thresholds are also shown, namely cells treated with incubation buffer (top left), stained with propidium iodide (bottom left) and stained with Annexin V (top right).
(B) Levels of BID mRNA in parental cells and clones transfected with a pTRIPZ BID vector, growing in absence (black bars) or presence (white bars) of 1 µg/mL doxocyclin.
(C) Dose-response curves for LY3295668 of PC9, PC9-GR4 and PC9-GR3 BID KO transfected with the pTRIPZ BID vector (SE1 and SE2 colonies). Ectopic expression of BID was induced using 1 µg/mL doxycycline. Cells numbers were determined by MTT at 72 h. Values shown are means ± SD of ≥2 independent experiments. In each experiment, every concentration of drug was tested in six wells (n=6).

## Slide 13
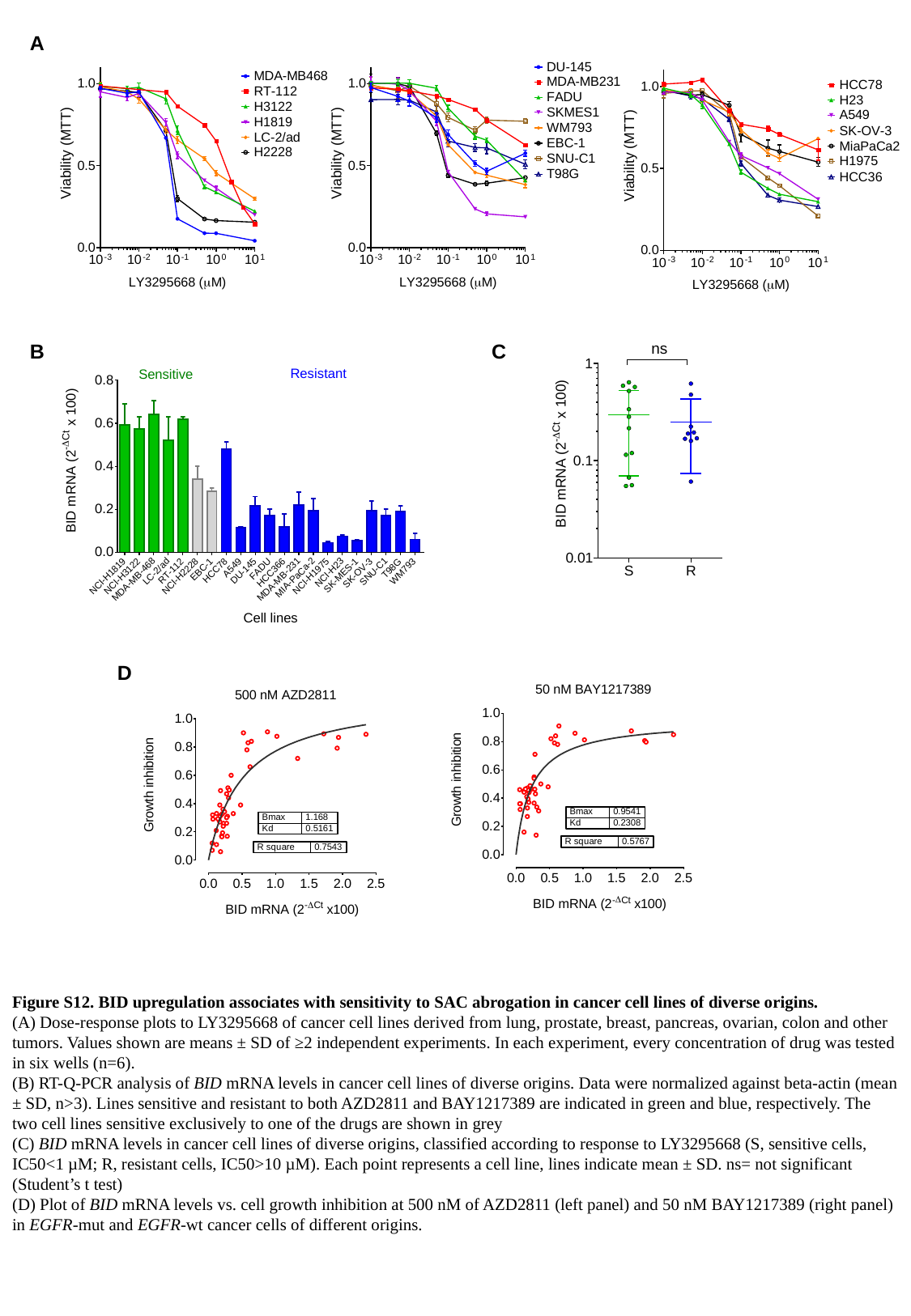

A
B
C
D
Figure S12. BID upregulation associates with sensitivity to SAC abrogation in cancer cell lines of diverse origins.
(A) Dose-response plots to LY3295668 of cancer cell lines derived from lung, prostate, breast, pancreas, ovarian, colon and other tumors. Values shown are means ± SD of ≥2 independent experiments. In each experiment, every concentration of drug was tested in six wells (n=6).
(B) RT-Q-PCR analysis of BID mRNA levels in cancer cell lines of diverse origins. Data were normalized against beta-actin (mean ± SD, n>3). Lines sensitive and resistant to both AZD2811 and BAY1217389 are indicated in green and blue, respectively. The two cell lines sensitive exclusively to one of the drugs are shown in grey
(C) BID mRNA levels in cancer cell lines of diverse origins, classified according to response to LY3295668 (S, sensitive cells, IC50<1 µM; R, resistant cells, IC50>10 µM). Each point represents a cell line, lines indicate mean ± SD. ns= not significant (Student’s t test)
(D) Plot of BID mRNA levels vs. cell growth inhibition at 500 nM of AZD2811 (left panel) and 50 nM BAY1217389 (right panel) in EGFR-mut and EGFR-wt cancer cells of different origins.

## Slide 14
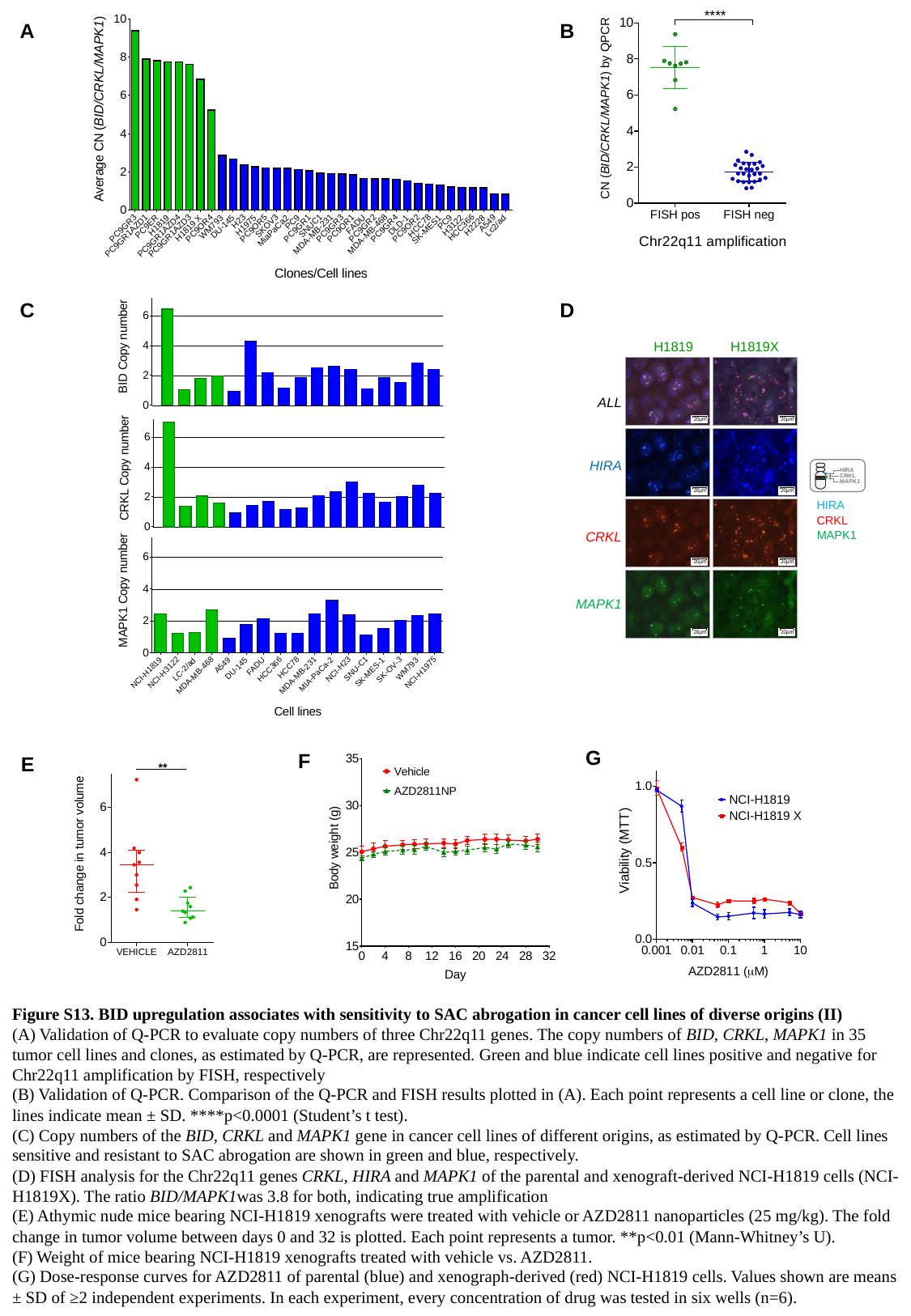

A
B
C
D
H1819X
H1819
ALL
HIRA
HIRA
CRKL
MAPK1
CRKL
MAPK1
G
F
E
Figure S13. BID upregulation associates with sensitivity to SAC abrogation in cancer cell lines of diverse origins (II)
(A) Validation of Q-PCR to evaluate copy numbers of three Chr22q11 genes. The copy numbers of BID, CRKL, MAPK1 in 35 tumor cell lines and clones, as estimated by Q-PCR, are represented. Green and blue indicate cell lines positive and negative for Chr22q11 amplification by FISH, respectively
(B) Validation of Q-PCR. Comparison of the Q-PCR and FISH results plotted in (A). Each point represents a cell line or clone, the lines indicate mean ± SD. ****p<0.0001 (Student’s t test).
(C) Copy numbers of the BID, CRKL and MAPK1 gene in cancer cell lines of different origins, as estimated by Q-PCR. Cell lines sensitive and resistant to SAC abrogation are shown in green and blue, respectively.
(D) FISH analysis for the Chr22q11 genes CRKL, HIRA and MAPK1 of the parental and xenograft-derived NCI-H1819 cells (NCI-H1819X). The ratio BID/MAPK1was 3.8 for both, indicating true amplification
(E) Athymic nude mice bearing NCI-H1819 xenografts were treated with vehicle or AZD2811 nanoparticles (25 mg/kg). The fold change in tumor volume between days 0 and 32 is plotted. Each point represents a tumor. **p<0.01 (Mann-Whitney’s U).
(F) Weight of mice bearing NCI-H1819 xenografts treated with vehicle vs. AZD2811.
(G) Dose-response curves for AZD2811 of parental (blue) and xenograph-derived (red) NCI-H1819 cells. Values shown are means ± SD of ≥2 independent experiments. In each experiment, every concentration of drug was tested in six wells (n=6).

## Slide 15
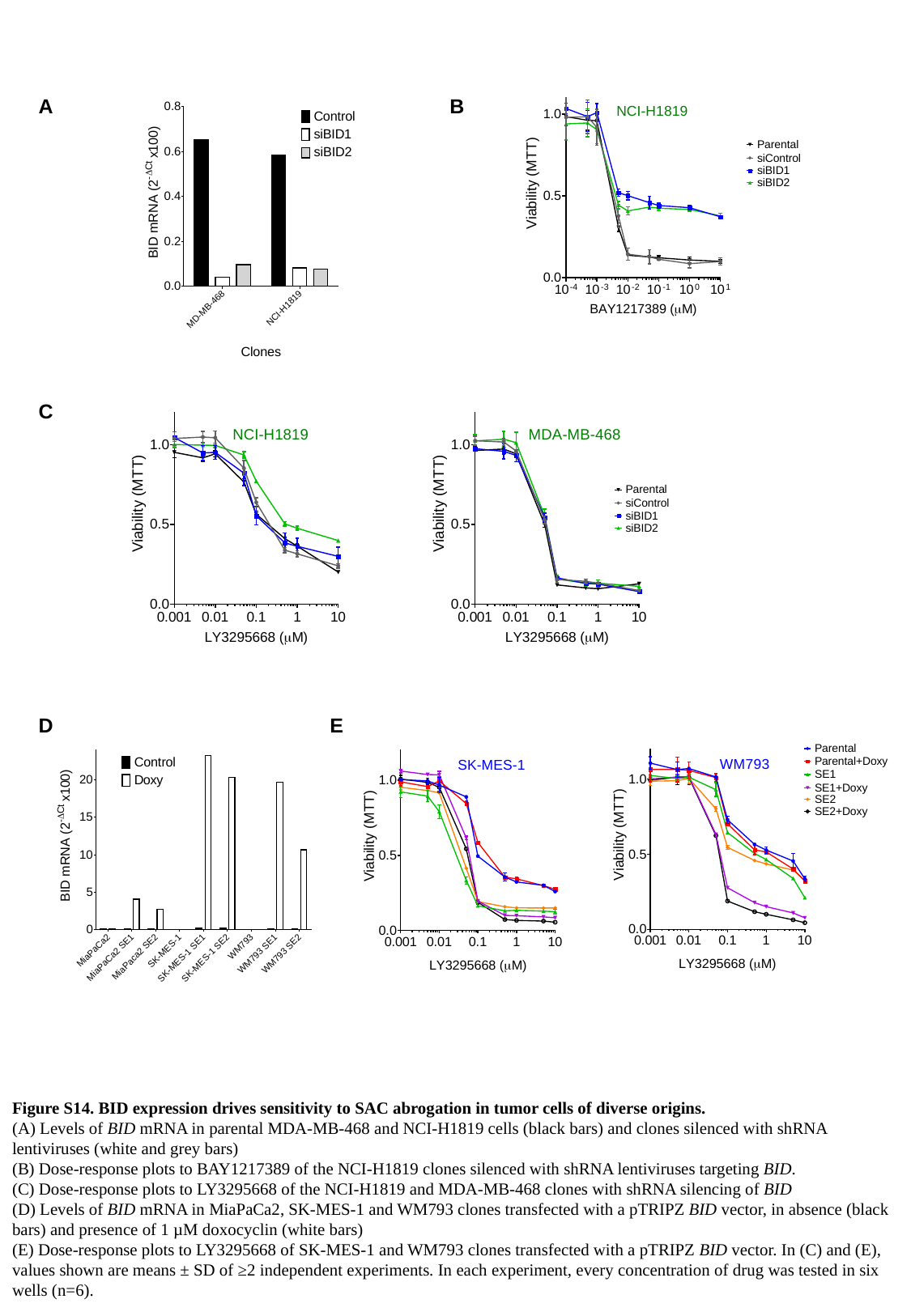

A
B
C
D
E
Figure S14. BID expression drives sensitivity to SAC abrogation in tumor cells of diverse origins.
(A) Levels of BID mRNA in parental MDA-MB-468 and NCI-H1819 cells (black bars) and clones silenced with shRNA lentiviruses (white and grey bars)
(B) Dose-response plots to BAY1217389 of the NCI-H1819 clones silenced with shRNA lentiviruses targeting BID.
(C) Dose-response plots to LY3295668 of the NCI-H1819 and MDA-MB-468 clones with shRNA silencing of BID
(D) Levels of BID mRNA in MiaPaCa2, SK-MES-1 and WM793 clones transfected with a pTRIPZ BID vector, in absence (black bars) and presence of 1 µM doxocyclin (white bars)
(E) Dose-response plots to LY3295668 of SK-MES-1 and WM793 clones transfected with a pTRIPZ BID vector. In (C) and (E), values shown are means ± SD of ≥2 independent experiments. In each experiment, every concentration of drug was tested in six wells (n=6).

## Slide 16
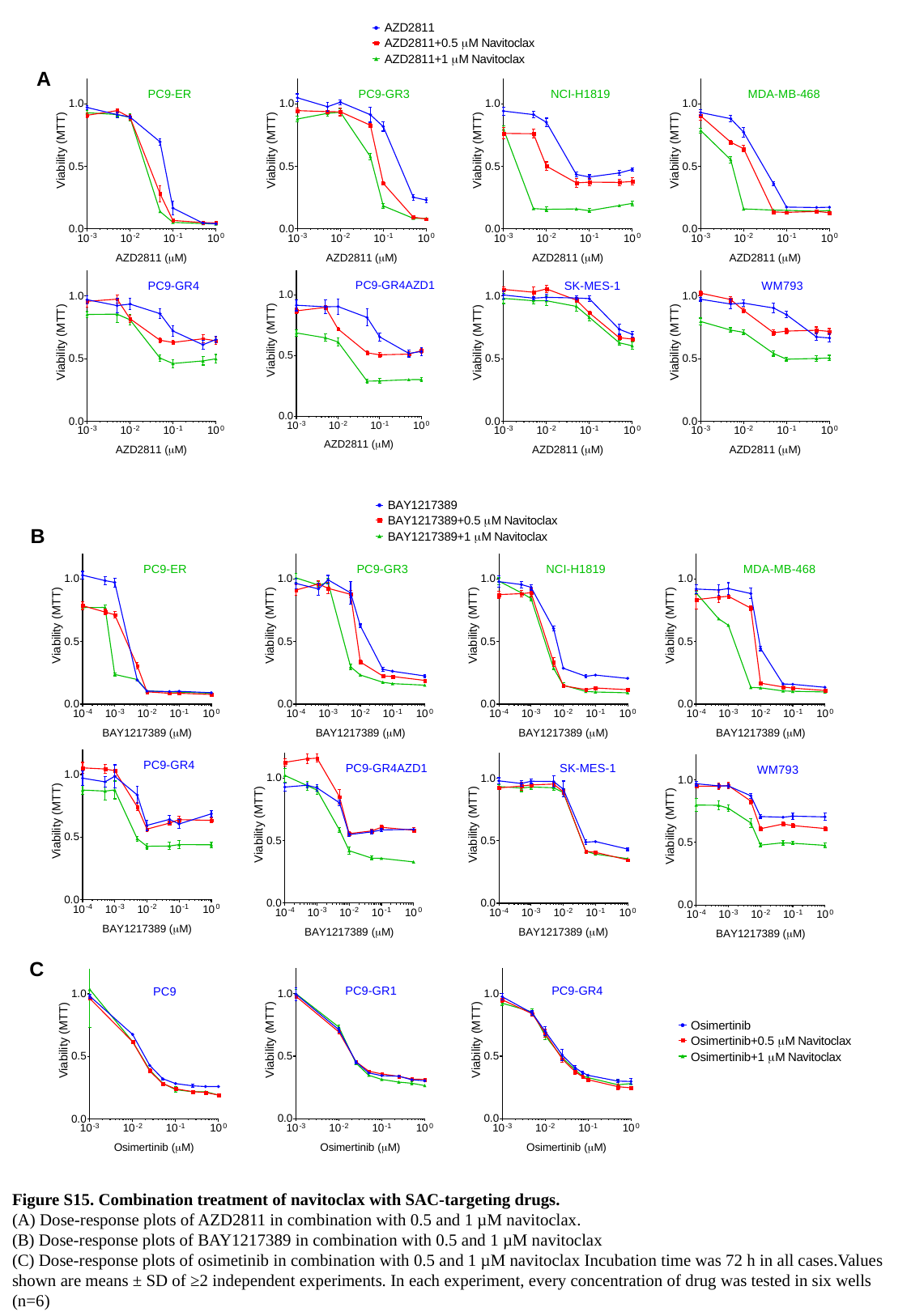

A
B
C
Figure S15. Combination treatment of navitoclax with SAC-targeting drugs.
(A) Dose-response plots of AZD2811 in combination with 0.5 and 1 µM navitoclax.
(B) Dose-response plots of BAY1217389 in combination with 0.5 and 1 µM navitoclax
(C) Dose-response plots of osimetinib in combination with 0.5 and 1 µM navitoclax Incubation time was 72 h in all cases.Values shown are means ± SD of ≥2 independent experiments. In each experiment, every concentration of drug was tested in six wells (n=6)

## Slide 17
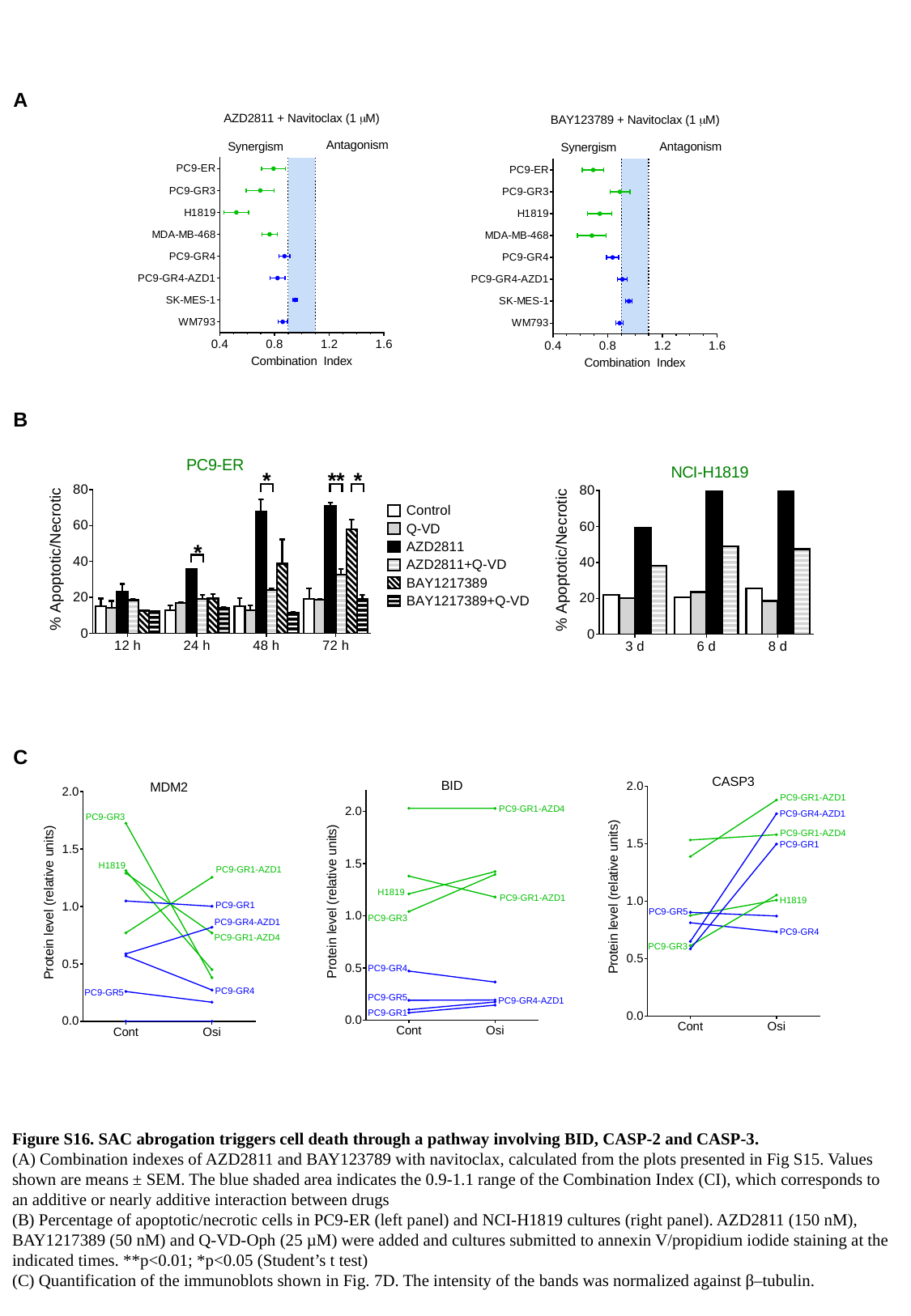

A
B
C
Figure S16. SAC abrogation triggers cell death through a pathway involving BID, CASP-2 and CASP-3.
(A) Combination indexes of AZD2811 and BAY123789 with navitoclax, calculated from the plots presented in Fig S15. Values shown are means ± SEM. The blue shaded area indicates the 0.9-1.1 range of the Combination Index (CI), which corresponds to an additive or nearly additive interaction between drugs
(B) Percentage of apoptotic/necrotic cells in PC9-ER (left panel) and NCI-H1819 cultures (right panel). AZD2811 (150 nM), BAY1217389 (50 nM) and Q-VD-Oph (25 µM) were added and cultures submitted to annexin V/propidium iodide staining at the indicated times. **p<0.01; *p<0.05 (Student’s t test)
(C) Quantification of the immunoblots shown in Fig. 7D. The intensity of the bands was normalized against β–tubulin.

## Slide 18
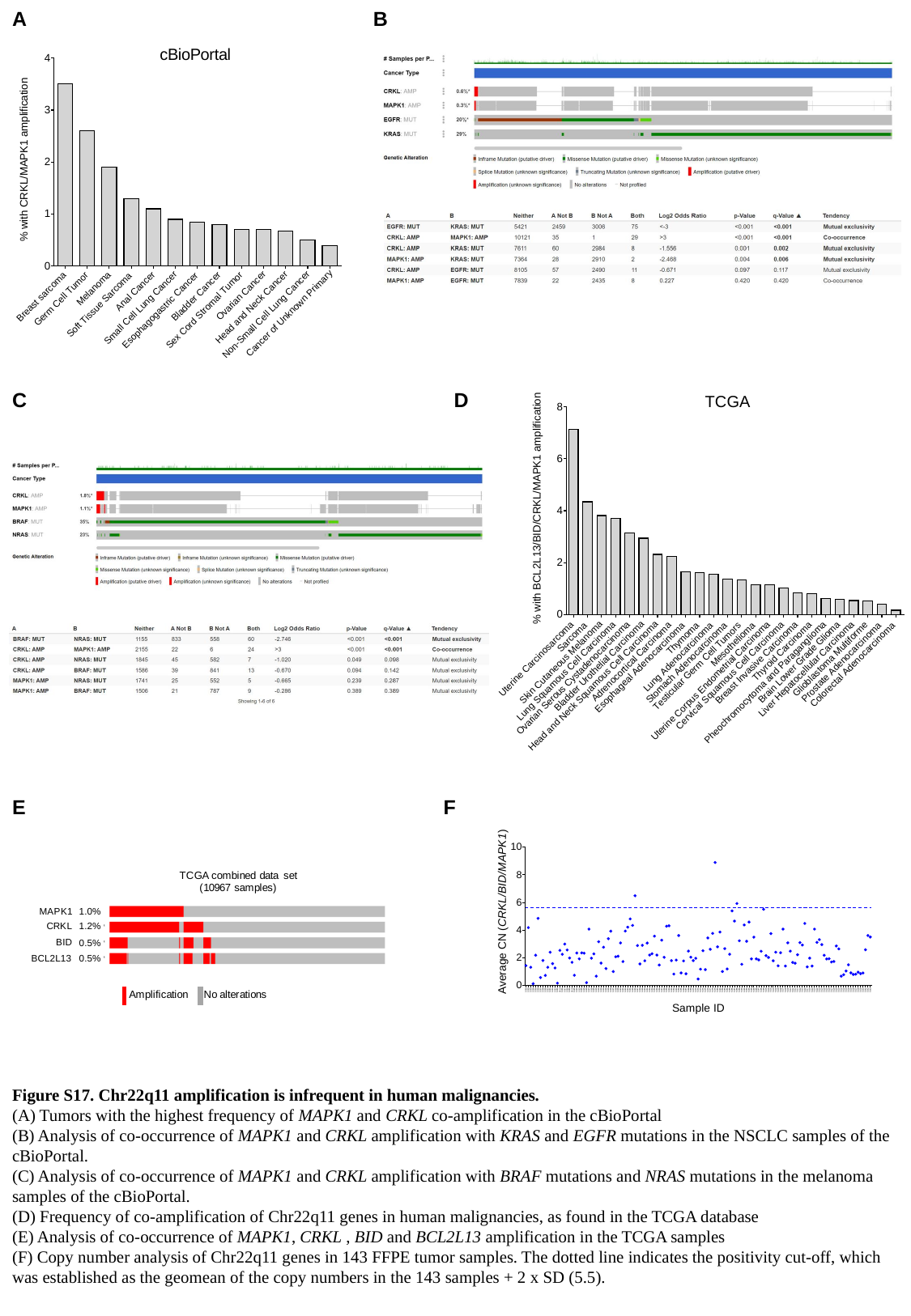

A
B
C
D
E
F
Figure S17. Chr22q11 amplification is infrequent in human malignancies.
(A) Tumors with the highest frequency of MAPK1 and CRKL co-amplification in the cBioPortal
(B) Analysis of co-occurrence of MAPK1 and CRKL amplification with KRAS and EGFR mutations in the NSCLC samples of the cBioPortal.
(C) Analysis of co-occurrence of MAPK1 and CRKL amplification with BRAF mutations and NRAS mutations in the melanoma samples of the cBioPortal.
(D) Frequency of co-amplification of Chr22q11 genes in human malignancies, as found in the TCGA database
(E) Analysis of co-occurrence of MAPK1, CRKL , BID and BCL2L13 amplification in the TCGA samples
(F) Copy number analysis of Chr22q11 genes in 143 FFPE tumor samples. The dotted line indicates the positivity cut-off, which was established as the geomean of the copy numbers in the 143 samples + 2 x SD (5.5).

## Slide 19
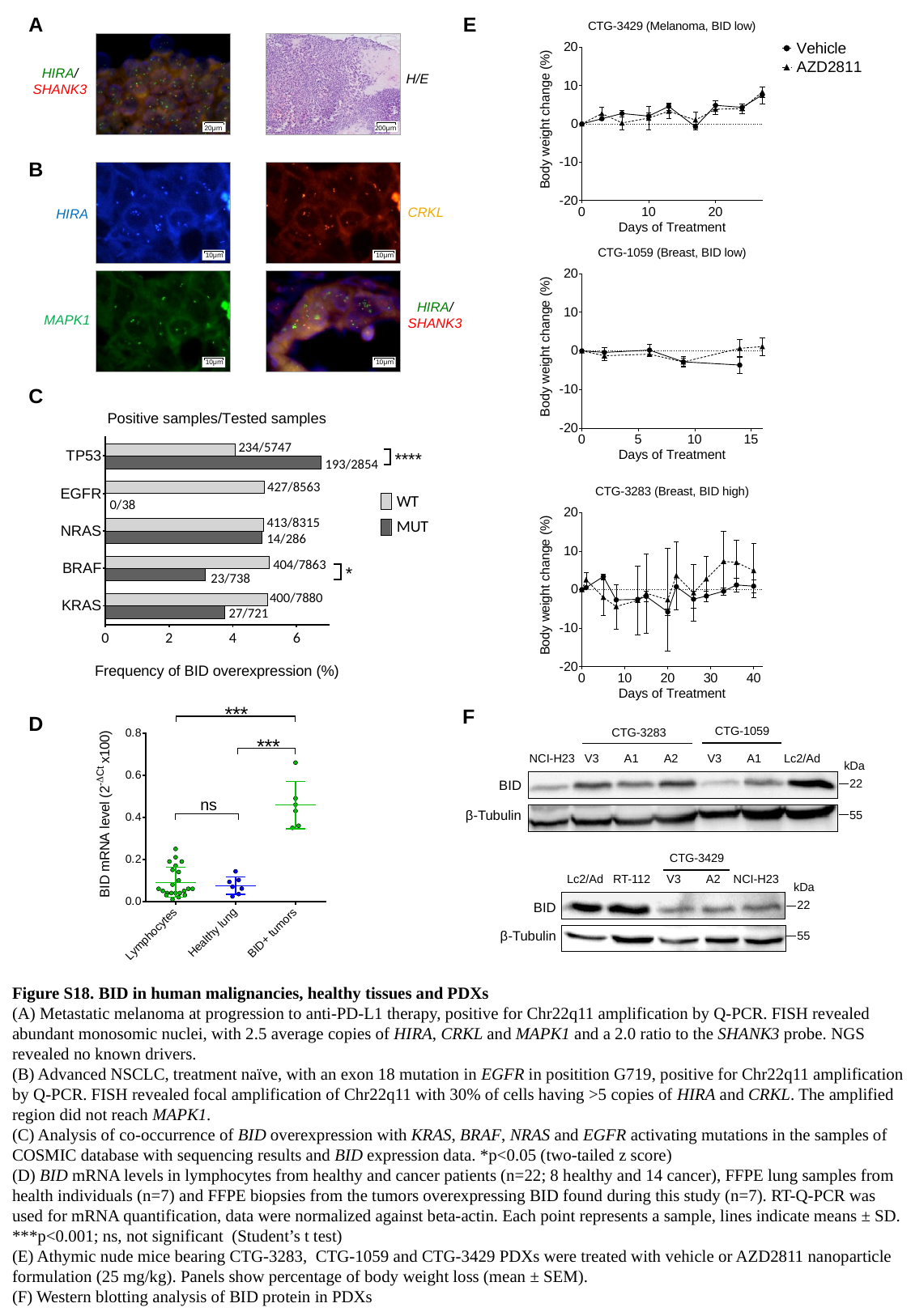

A
E
HIRA/
SHANK3
H/E
 20μm
 200μm
B
CRKL
HIRA
 10μm
 10μm
HIRA/
SHANK3
MAPK1
 10μm
 10μm
C
F
D
CTG-1059
CTG-3283
NCI-H23 V3 A1 A2 V3 A1 Lc2/Ad
kDa
22
BID
β-Tubulin
55
CTG-3429
Lc2/Ad RT-112 V3 A2 NCI-H23
kDa
22
BID
β-Tubulin
55
Figure S18. BID in human malignancies, healthy tissues and PDXs
(A) Metastatic melanoma at progression to anti-PD-L1 therapy, positive for Chr22q11 amplification by Q-PCR. FISH revealed abundant monosomic nuclei, with 2.5 average copies of HIRA, CRKL and MAPK1 and a 2.0 ratio to the SHANK3 probe. NGS revealed no known drivers.
(B) Advanced NSCLC, treatment naïve, with an exon 18 mutation in EGFR in positition G719, positive for Chr22q11 amplification by Q-PCR. FISH revealed focal amplification of Chr22q11 with 30% of cells having >5 copies of HIRA and CRKL. The amplified region did not reach MAPK1.
(C) Analysis of co-occurrence of BID overexpression with KRAS, BRAF, NRAS and EGFR activating mutations in the samples of COSMIC database with sequencing results and BID expression data. *p<0.05 (two-tailed z score)
(D) BID mRNA levels in lymphocytes from healthy and cancer patients (n=22; 8 healthy and 14 cancer), FFPE lung samples from health individuals (n=7) and FFPE biopsies from the tumors overexpressing BID found during this study (n=7). RT-Q-PCR was used for mRNA quantification, data were normalized against beta-actin. Each point represents a sample, lines indicate means ± SD. ***p<0.001; ns, not significant (Student’s t test)
(E) Athymic nude mice bearing CTG-3283, CTG-1059 and CTG-3429 PDXs were treated with vehicle or AZD2811 nanoparticle formulation (25 mg/kg). Panels show percentage of body weight loss (mean ± SEM).
(F) Western blotting analysis of BID protein in PDXs
