# Supplementary material for: BID expression determines the apoptotic fate of cancer cells after abrogation of the spindle assembly checkpoint by AURKB or TTK inhibitors
Source: Mol Cancer. 2023 Jul 13;22:110. doi: 10.1186/s12943-023-01815-w (PMC10339641; doi:10.1186/s12943-023-01815-w)
Supplement: Supplementary file 1 — Additional file 1. [file 12943_2023_1815_MOESM1_ESM.zip › Suppl_Methods_rev2.docx]

Supplementary Methods

Bertran-Alamillo et al.

**Cell culture and cell line genotyping**

All cell culture reagents were acquired from Gibco/Thermo Fisher Scientific (Waltham, MA), unless otherwise specified. All tumor cells were grown in a humidified atmosphere with 5% CO2 at 37ºC in RPMI 1640 + 10% fetal bovine serum (FBS), 50 µg/mL penicillin-streptomycin and 2 mM L-Glutamine.

Cell lines and resistant clones were routinely tested for mycoplasm contamination and authenticated by analyzing >20 polymorphisms using a GeneRead® QIAact Lung DNA UMI NGS panel (Qiagen, Hilden, Germany; see also under “Sequencing Techniques”). The presence of driver alterations (mutations, copy number variations, gene fusions and splicing variants) was also tested by NGS and nCounter in all the cell lines and clones used in this study. The panels used and further details of the techniques can be found in the “RT-Q-PCR and nCounter” and “Sequencing Techniques” sections. In all cases, the genotype of the parental cell lines determined in our laboratory was fully coincident with the genotype reported in the COSMIC [1] and Cellosaurus [2] databases. Regarding the resistant clones, the polymorphism profile was systematically coincident with the corresponding parental cells while the driver *EGFR* mutation was occasionally lost (see Table S13).

**Animal models**

Female, 5-6 months old, athymic nude B-NDG mice (Envigo, Indianapolis, IN) were subcutaneously implanted with tumor fragments of NCI-H1819 xenografts previously grown in B-NDG mice. Treatments were started when the average tumor size reached 150-200 mm^3^ and mice were assigned into groups using randomized block design based upon their tumor volumes. Tumor dimensions were measured three times per week using digital calipers and volumes were estimated according to the formula V= π/6 x L x W^2^, where L is the long axis and W is the short axis of tumor, respectively. Weight and general behavior of the mice were also monitored. Animals were sacrificed after 4 weeks of treatment or earlier if they met the criteria to apply an ethical endpoint; e. g., weight loss of more than 20% of the total body weight, tumor volume greater than 2000 mm^3^, tumor ulceration or any sign of suffering. AZD2811 was administered intravenously at doses 25 mg/kg in four cycles per week, vehicle was saline (0.9%). Osimertinib was administered by oral gavage at dose of 25mg/kg.

PC9-R5 cells were cultured in RPMI1640 (Gibco 61870-010) + 10% FCS (Sigma F7524) supplemented with 160nM Osimertinib (SN1029830886). Nonobese diabetic/severe combined immunodeficiency mice (NOD/SCID; The Jackson Laboratory, Bar Harbor, ME) were injected with 5x10^6^ cells in 100 uL Matrigel:PBS (1:1) subcutaneously in the left flank. Osimertinib treatment (5 mg/kg by oral gavage) was started the day after tumor implant. Pre-study tumor volumes were recorded for each experiment beginning seven to ten days after implantation. When tumors reached an average of 150-300 mm³ animals were matched by tumor volumes and divided into the Osimertinib (5mg/kg) and AZD2811 (25mg/kg) groups.

Tumor fragments from patient-derived xenografts (PDXs) CTG-3283, CTG-3429, CTG-1059 and ST3632 were harvested from stock animals and implanted unilaterally on the left flank of Athymic Nude-Foxn1nu female mice 6-12 weeks of age (The Jackson Laboratory). Tumor volumes were recorded for each experiment beginning seven to ten days after implantation. When tumors reached an average volume of 150-300 mm³ animals were matched by tumor volume into treatment or control groups to be used for dosing, which was initiated on Day 0. Upon the treatment initiation, tumor dimensions were measured twice weekly by digital caliper and tumor volumes calculated using the formula V= π/6 x L x W^2^. In addition, animals were observed daily and weighed twice weekly using a digital scale. AZD2811 was administered intravenously at 25 mg/kg dose once weekly and saline (0.9%) was used as a dilution vehicle. For hormone dependent models, exogenous hormones were supplied either via estradiol pellets (CTG-3283, CTG-1059) or in drinking water (ST3632).

NSCLC PDX models (DFCI-403, CTG-2939, DCFI-367) were implanted unilaterally on the lower right flank of NOD scid gamma (NSG) female mice (The Jackson Laboratory). Tumor volumes were measured starting from around two weeks after implantation. When tumors reached an average volume around 150-200 mm³, animals were matched by tumor volumes into treatment and control groups. Dosing was initiated between 25- and 39-days post-implantation, tumor volumes were calculated as mentioned above. In addition, animals were observed daily and weighed twice weekly using a digital scale. AZD2811 was administered as specified above.

CTG-3283, CTG-1059, ST3632 and all NSCLC PDX models were used as a part of a multi-arm study, with arms of no relevance for this publication, sharing a common control arm (vehicle treated group) as a refinement and reduction strategy in animal use (3Rs). Hence, the same control group data will be used in different publications.

**Cell viability and cell cycle analysis**

Cells were seeded at a density of 2000-8000 per well in 96-well plates, allowed to attach for 24 h and treated with drugs for 2-3 doubling times. AZD2811 was kindly provided by AstraZeneca (Cambridge, UK), the rest of drugs were purchased from Selleckchem Chemicals (Houston, TX) or MedChem Express (Monmouth Junction, NJ). Each concentration of drug was tested in six wells (technical replicates) in each experiment. After treatment, cells were incubated with medium containing 0.75 mg/mL of Thiazolyl Blue tetrazolium bromide (MTT, Thermo Fisher) for 1-2 h at 37ºC, medium was removed and formazan crystals dissolved in 100 µL DMSO. Cell numbers were estimated by measuring the absorbance at 565 nm, using an Infinite MPlex microplate reader (Tecan, Männedorf, Switzerland). Drugs and combinations were tested in a minimum of two independent experiments performed on different days (biological replicates). Combination experiments and calculation of combination indexes (CIs) were performed according to the Bliss method.

For cell cycle and cell death analyses, cells were plated in T-25 flasks, allowed to attach, treated with drugs in RPMI 1640 + 10% FBS, trypsinized and centrifuged. Cell pellets were resuspended in PBS, fixed in 70% ethanol and incubated o/n at -20 ºC. Fixed cells were subsequently re-centrifuged at 400 xg for 5 min at 4 ºC; resuspended in 250 µL of PBS with 50 µg/mL RNAse A (Sigma Aldrich, Saint Louis, MO), incubated for 1 h at 37 ºC and stained with propidium iodide (PI) (Roche Diagnostics, Basel, Switzerland) for 30 min at room temperature.

**Cell transfection**

All transfections were performed on 96-well plates by adding the lentiviral particles (MOI 0.5–5) to exponentially growing, 50–70% confluent cells in presence of hexadimethrine bromide (8 μg/mL) (Sigma-Aldrich). After a 24 h incubation, the viral particle-containing medium was replaced by fresh RPMI + 10% FBS. After an additional 24 h period, new medium was added containing 1 μg/mL puromycin (Sigma–Aldrich) for selection of transfected cells. Resistant colonies were picked up, expanded and assayed for the silencing or ectopic expression of the target gene. The sequences of the 253 sgRNAs, shRNAs and plasmids used for gene knock-out and ectopic expression are available upon request.

**Cell death, senescence, phenotypic reversion and essentiality score assays**

For cell death analyses, the Annexin-V-FLUOS (An) staining kit (Roche Diagnostics) was employed, according to the manufacturer’s instructions. Stained cells were sorted with a FACSCanto II cytometer (BD Biosciences, Franklin Lakes, NJ) using the FACSDiva software version 6.1.2. The combination of Annexin V and PI was used to differentiate four cell populations; viable cells (An -/PI-), early apoptotic (An+/PI-), necrotic (An-/PI+) and later apoptotic/necrotic (An+/PI+). For senescence studies, cells were treated with the selected drugs in 6-well plates and the Senescence Beta-galactosidase Staining kit (Cell Signaling Technology, Danvers, MA) was used to evaluate the presence of senescent cells by direct observation under the microscope.

Phenotypic reversion refers to PC9-GR3 cells reverting from an apoptotic to a senescent phenotype after AZD2811 treatment when certain genes are knocked-out. For phenotypic reversion assays, arrayed CRISPRn KO cells were obtained from Synthego Corporation (Redwood City, CA, US) targeting 80 genes in PC9-GR3 cells (see results). Genome editing efficiency was profiled by Synthego and Inference of CRISPR Edits scores were generated. Cells were seeded at a density of 400-600 cells/well in 384 well plates, allowed to adhere for 24 hours and stained with Incucyte® Nuclight Rapid Red Dye for Live-Cell Nuclear Labelling and Incucyte® Caspase-3/7 Dye for Apoptosis (Sartorius, Gotinga, Germany) at concentrations of 1:500 and 1:1000, respectively. Cells were then treated with AZD2811 or DMSO, subsequently placed in Incucyte® S3 Live-Cell Analysis System (Sartorius) for 60 hours and imaged every 30 minutes at 20x according to manufacturers’ recommendations. Images were analyzed with Incucyte® Software v2019B (Sartorius). To evaluate the phenotypic reversion, the average nuclear size per mm^2^ and the number of caspase-3/7 positive cells per mm^2^ was quantified. Fold change in nuclear size was calculated with the formula (average nuclear size of KO/ average nuclear size of PC9-GR3) x 100. Fold change in apoptosis was determined by (percentage of caspase-3/7 positive KO cells/ average percentage of positive PC9-GR3 caspase-3/7 cells) x 100. The combination of a reduced apoptosis and increased nuclear size in comparison to PC9-GR3 resulted in the classification of a senescent phenotype whilst an increase in apoptosis without nuclei enlargement resulted in an apoptotic phenotype classification. Essentiality scores were also determined by quantifying the rate of proliferation in comparison to PC9-GR3 using the following formula in DMSO-treated cells (((change in nuclear count over 58 hours x 1)/(58-1)) x 100)/ (((change in GR3 nuclear count over 58 hours x 1)/(58-1)) x 100).

**Western blotting**

Cells were seeded in T-75 flasks, allowed to attach o/n and treated for 2-72 h in RPMI+10% FBS, unless otherwise indicated. Cultures were then washed twice with cold PBS, scrapped into RIPA buffer (Cell Signaling Technology) with a protease inhibitors cocktail (Roche Diagnostics) and passed through an insulin syringe. Lysates were transferred to a microfuge tube, incubated on ice 15 minutes, centrifuged for 10 minutes at 14,000 rpm and immediately analyzed or frozen at –80 ºC. After protein quantification using the Pierce BCA Protein Assay (Thermo Fisher), extracts (25 µg) were boiled in NuPAGE-LDS sample (Invitrogen, Carlsbad, CA) with 0.1% β-mercaptoethanol, resolved in 8% SDS-polyacrylamide gels and transferred to a PVDF membrane (Merck Millipore, Burlington, MA). Membranes were blocked for 1 h in Tris Buffered Saline (TBS, Bio-Rad, Hercules, CA) with 3% dry fat-free milk. After blocking, membranes were cut, incubated with primary antibodies o/n at 4 ºC, washed three times for 5 min each in PBS-Tween 0.1% (Bio-Rad) and incubated for 2 h with secondary antibody. Finally, after three additional washes, membranes were soaked in Supersignal Chemiluminiscence substrate (Thermo Fisher) and read with a Bio-Rad ChemiDocMP Imaging System. ImageJ and ImageLab softwares were used to quantify bands.

**DNA and RNA purification**

In the case of FFPE tumor samples, 4 μm-slides were obtained by standard procedures and stained with hematoxylin and eosin. An expert pathologist determined the tumor area and evaluated the percentage of tumor infiltration. In samples with <20% tumor, manual macrodissection or laser capture microdissection (Zeiss, Jena, Germany) was used to ensure a minimum of 80% of tumor cells in the starting material for nucleic acid purification. RNA was extracted with a high purity FFPE RNA isolation kit (Roche Diagnostics), while the GeneRead DNA FFPE Kit or the QIAamp DNA FFPE Tissue Kit (Qiagen) were used for DNA extraction, according to the manufacturer’s instructions. For DNA and RNA purification from cultured cells and lymphocytes, the DNeasy® Blood & Tissue Kit (Qiagen) and the High Pure RNA isolation Kit (Roche Diagnostics) were employed. DNA utilized in WES was extracted from flash frozen cell pellets using the AllPrep DNA/RNA Micro Kit (Qiagen) and eluted in TE buffer, purity was determined by NanoDrop 8000 (Thermo Fisher) and DNA integrity was measured using a 4200 TapeStation (Agilent). All samples had a DIN of >7.0. In all cases, final concentrations of nucleic acids were measured by Qubit (Thermo Fisher).

**Q-PCR**

Q-PCR was used to estimate copy numbers. Template DNA was added to Taqman Genotyping Master Mix (Applied Biosystems, Pleasanton, CA) in 10 µl reactions containing specific primers and probes for each gene analyzed (*BID*, *MAPK1*, *CRLK* and *SHANK3*). The primer and probe sets were purchased as Taqman^TM^ Copy Number Assays (Applied Biosystems), *TERT* was selected as a reference gene and acquired as a Taqman^TM^ Copy Number Reference assay. Four reactions were run per sample, each one containing the assay for one of the tested genes plus the reference assay. All reactions were run in a QuantStudio 6 Flex (Applied Biosystems). After an initial denaturation step (95ºC, 10 min), thermal protocol was 40 cycles of 15 sec at 95ºC followed by 60 sec at 60ºC. To estimate copy numbers, we calculated the 2^-ΔCt^ for each gene vs. the reference gene, which was subsequently normalized with the geometric mean of 2^-ΔCt^ values of the entire patient cohort multiplied by two. A sample was considered not evaluable and repeated when the standard deviation of the Ct values of the *TERT* gene were > 0.30. The methodology was validated by comparison with the gold standard (FISH) in a panel of cell lines (Figure S13A-B). Then, 143 FFPE tumor tissues were analyzed and the cut-off for positivity was established as the geomean + 2 x standard deviation of the copy numbers obtained (Fig S17F), which was validated by submitting 17 selected samples to FISH.

**RT-Q-PCR and nCounter**

Total RNA was converted into cDNA using the M-MLV reverse transcriptase enzyme (Invitrogen, Carlsbad, CA). Quantification of gene expression was performed using a QuantStudio 6 Flex (Applied Biosystems). Template cDNA was added to Taqman Universal Master Mix (Applied Biosystems) in triplicate 12.5 µl reactions with specific primers and probes for each gene, as described [3]. Primer and probe sets for *BID* and *ACTB* (reference gene) were designed using Primer Express 3.0 Software (Applied Biosystems) according to their Ref Seq (http://www.ensembl.org/index.html) and are listed in Table S3. In the case of *BCL2L13*, *CRKL1*, *MAPK1* and *MAPK3*, primers and probes were purchased as Taqman^TM^ Gene expression assays. Levels of mRNA were quantified according to the comparative ΔCt method using *ACTB* (β-actin) as endogenous gene for normalization [3]. A sample was considered not evaluable and repeated when the standard deviation of the Ct values of the three replicates was > 0.30. nCounter was performed with a custom panel (Table S4), the IO360 panel or a fusion panel, according to the manufacturer’s instructions (Nanostring, Seattle, WA). The 96 probes A and B of the custom panel were designed by Nanostring and are available upon request. The fusion panel was used for genotyping of cell lines and targets gene fusions in *ALK*, *ROS1*, *RET* and *NTRK1-3*, together with *EGFRvIII* and *METΔ14* splicing variants [4, 5]. Negative controls were used for background correction and the resulting counts were normalized using the housekeeping genes of the panel, as described [4, 5].

**Sequencing techniques**

DNA-based next generation sequencing (NGS) was performed according to the manufacturer’s instructions with a GeneRead® QIAact Lung DNA UMI Panel (Qiagen), which targets the following 30 genes frequently altered in cancer; *EGFR, BRAF, MET, ERBB2, ALK, ROS1, RET, PIK3CA, KRAS, NRAS, KIT, PDGFRA, TP53, STK11, KEAP1, ARID1A, FAT1, NFE2L2, SETD2, POLE, POLD1, IDH1, IDH2, ERBB4, FGFR1, FGFR2, FGFR3, MYC, CDK4, CDK6*. Up to 40 ng of purified DNA were used as a template. Clonal amplification was performed on pooled libraries (625 pg) and, following bead enrichment, the GeneReader instrument was used for sequencing. Qiagen Clinical Insight Analyze (QCI-A) software was employed to align the read data and call sequence variants, which were imported into the Qiagen Clinical Insight Interpret (QCI-I) web interface for data interpretation and generation of final custom report. WES and WTS were performed as described [6, 7]. In the case of WTS, every sample was run three times. Comparison of the gene expression of the resistant clones vs. the parental PC9 cells was performed as follows. For every gene, we calculated the log2 fold in resistant vs. parental cells using the DESeq2 package (version 1.32.0) [8] for R (version 4.1.0) and the associated p-values using the z test functions contained in DESeq2. Then, we calculated q-values (p-values adjusted for multiple testing) using the fdrtool R package (version 1.2.17) for False Discovery Rate estimation [9]. We considered significative q-values < 0.05 (see Table S10).

REFERENCES

1. Tate JG, Bamford S, Jubb HC, Sondka Z, Beare DM, Bindal N, Boutselakis H, Cole CG, Creatore C, Dawson E, et al: **COSMIC: the Catalogue Of Somatic Mutations In Cancer.** *Nucleic Acids Res* 2019, **47:**D941-D947.

2. Bairoch A: **The Cellosaurus, a Cell-Line Knowledge Resource.** *J Biomol Tech* 2018, **29:**25-38.

3. Rosell R, Molina MA, Costa C, Simonetti S, Gimenez-Capitan A, Bertran-Alamillo J, Mayo C, Moran T, Mendez P, Cardenal F, et al: **Pretreatment EGFR T790M mutation and BRCA1 mRNA expression in erlotinib-treated advanced non-small-cell lung cancer patients with EGFR mutations.** *Clin Cancer Res* 2011, **17:**1160-1168.

4. Aguado C, Teixido C, Roman R, Reyes R, Gimenez-Capitan A, Marin E, Cabrera C, Vinolas N, Castillo S, Munoz S, et al: **Multiplex RNA-based detection of clinically relevant MET alterations in advanced non-small cell lung cancer.** *Mol Oncol* 2020, **15:**350-363.

5. Reguart N, Teixido C, Gimenez-Capitan A, Pare L, Galvan P, Viteri S, Rodriguez S, Peg V, Aldeguer E, Vinolas N, et al: **Identification of ALK, ROS1, and RET Fusions by a Multiplexed mRNA-Based Assay in Formalin-Fixed, Paraffin-Embedded Samples from Advanced Non-Small-Cell Lung Cancer Patients.** *Clin Chem* 2017, **63:**751-760.

6. Bertran-Alamillo J, Cattan V, Schoumacher M, Codony-Servat J, Gimenez-Capitan A, Cantero F, Burbridge M, Rodriguez S, Teixido C, Roman R, et al: **AURKB as a target in non-small cell lung cancer with acquired resistance to anti-EGFR therapy.** *Nat Commun* 2019, **10:**1812.

7. Jacobsen K, Bertran-Alamillo J, Molina MA, Teixido C, Karachaliou N, Pedersen MH, Castellvi J, Garzon M, Codony-Servat C, Codony-Servat J, et al: **Convergent Akt activation drives acquired EGFR inhibitor resistance in lung cancer.** *Nat Commun* 2017, **8:**410.

8. Love MI, Huber W, Anders S: **Moderated estimation of fold change and dispersion for RNA-seq data with DESeq2.** *Genome Biol* 2014, **15:**550.

9. Strimmer K: **fdrtool: a versatile R package for estimating local and tail area-based false discovery rates.** *Bioinformatics* 2008, **24:**1461-1462.
