# Supplementary material for: BID expression determines the apoptotic fate of cancer cells after abrogation of the spindle assembly checkpoint by AURKB or TTK inhibitors
Source: Mol Cancer. 2023 Jul 13;22:110. doi: 10.1186/s12943-023-01815-w (PMC10339641; doi:10.1186/s12943-023-01815-w)
Supplement: Supplementary file 1 — Additional file 1. [file 12943_2023_1815_MOESM1_ESM.zip › Suppl_Tables_rev2.docx]

Supplementary Tables

Bertran-Alamillo et al.

**Table S1**. Cell lines used in the study

| **Cell line** | **Source** | **Identifier** |
| --- | --- | --- |
| 11-18 | Dr. Mayumi Ono | RRID:CVCL_6659 |
| A549 | ATCC | ATCC Cat#CCL-185; RRID:CVCL_0023 |
| DLD-1 | ATCC | ATCC Cat#CCL-221; RRID:CVCL_0248 |
| DU-145 | ATCC | ATCC Cat#HTB-81; RRID:CVCL_0105 |
| EBC-1 | JCRB Cell Bank | JCRB Cat#JCRB0820; RRID:CVCL_2891 |
| FaDu | ATCC | ATCC Cat#HTB-43; RRID:CVCL_1218 |
| H1975 | ATCC | ATCC Cat#CRL-5908; RRID:CVCL_1511 |
| H3122 | Creative Biolabs | Cat.No.#IOC-ZP173; RRID:CVCL_5160 |
| HCC366 | DSMZ | DSMZ Cat#ACC 492; RRID:CVCL_2059 |
| HCC78 | DSMZ | DSMZ Cat#ACC 563; RRID:CVCL_2061 |
| LC-2/ad | ECACC | ECACC Cat#94072247; RRID:CVCL_1373 |
| MDA-MB-231 | ATCC | ATCC Cat#CRM-HTB-26; RRID:CVCL_0062 |
| MDA-MB-468 | ATCC | ATCC Cat#HTB-132; RRID:CVCL_0419 |
| MIA PaCa-2 | ATCC | ATCC Cat#CRM-CRL-1420; RRID:CVCL_0428 |
| NCI-H1819 | ATCC | ATCC Cat#CRL-5897; RRID:CVCL_E099 |
| NCI-H2228 | ATCC | ATCC Cat#CRL-5935; RRID:CVCL_1543 |
| NCI-H23 | ATCC | ATCC Cat#CRL-5800; RRID:CVCL_1547 |
| PC9 | F.Hoffman La Roche Ltd | RRID:CVCL_B260 |
| RT-112 | DSMZ | DSMZ Cat#ACC 418; RRID:CVCL_1670 |
| SK-MES-1 | ATCC | ATCC Cat#HTB-58; RRID:CVCL_0630 |
| SK-OV-3 | ATCC | ATCC Cat#HTB-77; RRID:CVCL_0532 |
| SNU-C1 | ATCC | ATCC Cat#CRL-5972; RRID:CVCL_1708 |
| T98G | ATCC | ATCC Cat#CRL-1690; RRID:CVCL_0556 |
| WM115 | ATCC | ATCC Cat#CRL-1675; RRID:CVCL_0040 |
| WM793 | ATCC | ATCC Cat#CRL-2806; RRID:CVCL_8787 |

**Table S2**. Antibodies used in the study

| **Antibodies** | **Source** | **Identifier** |
| --- | --- | --- |
| Akt Antibody | Cell Signaling Technology | Cat# 9272; RRID:AB_329827 |
| Phospho-Akt (Ser473) Antibody | Cell Signaling Technology | Cat# 9271; RRID:AB_329825 |
| BID Antibody (Human Specific) | Cell Signaling Technology | Cat# 2002; RRID:AB_10692485 |
| Anti-Bim Antibody | Cell Signaling Technology | Cat# 2819; RRID:AB_10692515 |
| Anti-Caspase 2, clone 11B4 | Millipore | Cat# MAB3507; RRID:AB_94894 |
| BCL2L13 Polyclonal antibody | Proteintech | Cat# 16612-1-AP; RRID:AB_1850928 |
| Caspase-3 Antibody | Cell Signaling Technology | Cat# 9662; RRID:AB_331439 |
| [Caspase-9 Antibody (Human Specific)](https://www.cellsignal.com/products/primary-antibodies/caspase-9-antibody-human-specific/9502) | Cell Signaling Technology | Cat# 9502; RRID:AB_2068621 |
| Rabbit Anti-Cdc6 Monoclonal Antibody | Cell Signaling Technology | Cat# 3387; RRID:AB_2078525 |
| CDK2 (78B2) Rabbit mAb antibody | Cell Signaling Technology | Cat# 2546; RRID:AB_2276129 |
| CDK4 antibody | Cell Signaling Technology | Cat# 12790; RRID:AB_2631166 |
| CDK6 (D4S8S) Rabbit mAb antibody | Cell Signaling Technology | Cat# 13331; RRID:AB_2721897 |
| CDKN2A/p16INK4a antibody [EP4353Y] | Abcam | Cat# ab81278; RRID:AB_1640753 |
| CrkL (D4G7G) Rabbit mAb antibody | Cell Signaling Technology | Cat# 38710; RRID:AB_2799138 |
| Cyclin D1 (92G2) Rabbit mAb antibody | Cell Signaling Technology | Cat# 2978; RRID:AB_2259616 |
| Cyclin E1 (D7T3U) antibody | Cell Signaling Technology | Cat# 20808; RRID:AB_2783554 |
| EGF Receptor (D38B1) XP Rabbit mAb antibody | Cell Signaling Technology | Cat# 4267; RRID:AB_2246311 |
| Phospho-EGF Receptor (Tyr1068) (D7A5) XP Rabbit mAb | Cell Signaling Technology | Cat# 3777; RRID:AB_2096270 |
| Phospho-EGF Receptor (Tyr845) (D63B4) Rabbit mAb antibody | Cell Signaling Technology | Cat# 6963; RRID:AB_10839407 |
| Phospho-EGF Receptor (Tyr1173) (53A5) Rabbit mAb antibody | Cell Signaling Technology | Cat# 4407; RRID:AB_331795 |
| Anti-Histone H3 antibody | Abcam | Cat# ab1791; RRID:AB_302613 |
| Anti-phospho-Histone H3 (Ser10), Mitosis Marker antibody | Millipore | Cat#06-570 RRID:AB_310177 |
| HSP90 Antibody | Cell Signaling Technology | Cat# 4874; RRID:AB_2121214 |
| p44/42 MAPK (Erk1/2) Antibody | Cell Signaling Technology | Cat# 9102; RRID:AB_330744 |
| p44/42 MAP kinase (phosphorylated Erk1/2) antibody | Cell Signaling Technology | Cat# 9101; RRID:AB_331646 |
| Mcl-1 antibody | Cell Signaling Technology | Cat# 94296; RRID:AB_2722740 |
| MDM2 (D1V2Z) Rabbit mAb | Cell Signaling Technology | Cat# 86934; RRID:AB_2784534 |
| MERTK antibody [Y323] | Abcam | Cat# ab52968; RRID:AB_2143584 |
| MERTK (phospho Y749/753/754) antibody | Abcam | Cat# ab14921; RRID:AB_2250636 |
| Mouse Anti-PARP Monoclonal Antibody | Sigma-Aldrich | Cat# P248; RRID:AB_260915 |
| PRAS40 (D23C7) XP Rabbit mAb antibody | Cell Signaling Technology | Cat# 2691; RRID:AB_2225033 |
| Phospho-PRAS40 (Thr246) (C77D7) Rabbit mAb antibody | Cell Signaling Technology | Cat# 2997; RRID:AB_2258110 |
| Rb (4H1) Mouse mAb antibody | Cell Signaling Technology | Cat# 9309; RRID:AB_823629 |
| Phospho-Rb (Ser780) (C84F6) Rabbit mAb antibody | Cell Signaling Technology | Cat# 3590; RRID:AB_2177182 |
| Phospho-Rb (Ser807/811) (D20B12) XP Rabbit mAb antibody | Cell Signaling Technology | Cat# 8516; RRID:AB_11178658 |
| Anti-Phosphoserine, clone 4A4 (mouse monoclonal IgG1) | Millipore | Cat# 05-1000; RRID:AB_11210897 |
| Mouse Anti-p14 ARF Monoclonal Antibody | Cell Signaling Technology | Cat# 2407; RRID:AB_490785 |
| p21 Waf1/Cip1 (12D1) Rabbit monoclonal antibody | Cell Signaling Technology | Cat# 2947; RRID:AB_823586 |
| p53 (DO-1) antibody | Santa Cruz Biotechnology | Cat# sc-126; RRID:AB_628082 |
| Anti-rabbit IgG, HRP-linked Antibody | Cell Signaling Technology | Cat# 7074; RRID:AB_2099233 |
| Sheep Anti-Mouse IgG, Whole Ab ECL Antibody, HRP Conjugated | GE Healthcare | Cat# NXA931; RRID:AB_772209 |
| Goat Anti-Rat IgG, Whole Ab ECL Antibody, HRP Conjugated | GE Healthcare | Cat# NA935; RRID:AB 772207 |
| Anti-β-Tubulin antibody | Sigma-Aldrich | Cat# T8328; RRID:AB 1844090 |

**Table S3.** Primers and probes for RT-Q-PCR used in the study. Primers for *BID* and *ACTB* were designed in house. For the rest of genes, predesigned TaqMan® Assays (Applied Biosystems) were purchased, the assay ID numbers are indicated in the table

| **Primers/Probes/Assays** | **Sequences/Assay ID** |
| --- | --- |
| *BID Forward* | 5´-GAGCTGCAGACTGATGGCAAC-3´ |
| *BID Reverse* | 5´-TGGCAATATTCCGGATGATGT-3´ |
| *BID Probe* | FAM 5´-ATAGAGGCAGATTCTGAAAG-3´ MGB |
| *ACTB Forward* | 5´-CGTCTTCCCCTCCATCGTG-3´ |
| *ACTB Reverse* | 5´-CCTTCTGACCCATGCCCAC-3´ |
| *ACTB Probe* | FAM 5´-CACCAGGGCGTGATG-3´ MGB |
| TaqMan® Assay *BCL2L13* | [Hs00209789_m1](https://www.thermofisher.com/taqman-gene-expression/product/Hs00209789_m1?CID=&ICID=&subtype=) |
| TaqMan® Assay *CRKL* | Hs00178304_m1 |
| TaqMan® Assay *MAPK1* | [Hs01046830_m1](https://www.thermofisher.com/taqman-gene-expression/product/Hs01046830_m1?CID=&ICID=&subtype=) |
| TaqMan® Assay *MAPK3* | [Hs00385075_m1](https://www.thermofisher.com/taqman-gene-expression/product/Hs00385075_m1?CID=&ICID=&subtype=) |

**Table S4.** Genes targeted by the nCounter mRNA expression panel used in the study. Chr, chromosome; EMT, epithelial to mesenchymal transition; RTK, receptor tyrosine kinase; SAC, spindle assembly checkpoint

| **Gene** | **Function (protein)** | **Gene** | **Function (protein)** |
| --- | --- | --- | --- |
| *AKT* | Signal transduction | *CDKN2A* | Cell cycle (Rb pathway) (p14 ARF) |
| *ARHGAP35* | Senescence | *CHEK1* | Cell cycle (G2/M) |
| *AURKA* | Cell cycle (Mitosis, SAC) | *CHEK2* | Cell cycle (Mitosis) |
| *AURKB* | Cell cycle (Mitosis, SAC) | *CTNNB1* | EMT (β-Catenin) |
| *AXL* | RTK | *E2F* | Cell cycle (Rb pathway) |
| *BCL2* | Apoptosis | *EGFR* | RTK |
| *BCL2L11* | Apoptosis | *FN1* | EMT (Fibronectin) |
| *BIRC5* | Apoptosis | *FOXM1* | Cell cycle (Mitosis) |
| *CCNA2* | Cell cycle (G2/M) | *MCL1* | Apoptosis |
| *CCNB1* | Cell cycle (Mitosis) | *MDM2* | Cell cycle, apoptosis |
| *CCND1* | Cell cycle (Rb pathway) | *MERTK* | RTK |
| *CCND2* | Cell cycle (Rb pathway) | *MET* | RTK |
| *CCND3* | Cell cycle (Rb pathway) | *PARP1* | Apoptosis |
| *CCNE1* | Cell cycle (Rb pathway) | *PTEN* | Signal transduction |
| *CCNE2* | Cell cycle (Rb pathway) | *RB1* | Cell cycle (Rb pathway) |
| *CDC20* | Cell cycle (Mitosis) | *RRM2* | Cell cycle (S-phase) |
| *CDC6* | Cell cycle (S-phase) | *SNAI1* | EMT (Snail) |
| *CDH1* | EMT (E-cadherin) | *SNAI2* | EMT (Slug) |
| *CDH2* | EMT (N-cadherin) | *TP53* | Cell cycle, apoptosis |
| *CDK2* | Cell cycle (Rb pathway) | *TTK* | Cell cycle (SAC) |
| *CDK4* | Cell cycle (Rb pathway) | *VIM* | EMT (Vimentin) |
| *CDK6* | Cell cycle (Rb pathway) | *ACTB* | Housekeeping |
| *CDKN1A* | Cell cycle (Rb pathway) (p21) | *GAPDH* | Housekeeping |
| *CDKN2A* | Cell cycle (Rb pathway) (p16INK4a) | *PSMC4* | Housekeeping |

**Table S5.** Sensitivity to selected inhibitors of the PC9 and 11-18-derived clones used in the study (first panel). AZD2811 and LY3295668 are specific for AURKB and AURKA, respectively. BAY1217389 is a TTK inhibitor; selumetinib a MEK inhibitor and pictilisib an AKT inhibitor. The IC50s and the % of survival in MTT assays at 500 nM (for AZD2811 and LY3295669) or 50 nM inhibitor (for BAY1217389) are indicated. P, parental; G, gefitinib; E, erlotinib; O, osimertinib; Act, activation; ov, overexpression. Td, doubling time. Values are means ± SD of ≥ 3 independent experiments.

| **Cells** | **Res.**  **to** | **Mechanism**  **of resist** | **Td (h)** | **AZD2811 (AURKBi)** | |  | **LY3295668 (AURKAi)** | |  | **BAY1217389 (TTKi)** | |  | **Cisplatin** | **Selumetinib** | **Pictilisib** | |
| --- | --- | --- | --- | --- | --- | --- | --- | --- | --- | --- | --- | --- | --- | --- | --- | --- |
|  |  |  |  | **IC50(µM)** | **%** |  | **IC50(µM)** | **%** |  | **IC50(µM)** | **%** |  | **IC50(µM)** | **IC50(µM)** | **IC50(µM)** | |
| PC9 | P | -- | 24±2 | **>10** | 51 ± 5 |  | **0.11 ± 0.01** | 46 ± 2 |  | **>1** | 61 ± 5 |  | 2.96 ± 0.31 | >10 | 1.78 ± 0.16 | |
| PC9-GR1 | G | T790M | 27±3 | **>10** | 49 ± 5 |  | **0.09 ± 0.01** | 26 ± 2 |  | **>1** | 57 ± 4 |  | 1.67 ± 0.23 | >10 | 2.08 ± 0.15 | |
| PC9-GR2 | G | MET act. | 31±1 | **>10** | 70 ± 3 |  | **0.04 ± 0.00** | 18 ± 0 |  | **>1** | 63 ± 4 |  | 1.90 ± 0.30 | >10 | 2.29 ± 0.38 | |
| PC9-GR3 | G | Unknown | 30±1 | **0.05 ± 0.00** | 9 ± 1 |  | **0.05 ± 0.00** | 13 ± 0 |  | **0.0036 ± 0.0002** | 14 ± 1 |  | 1.86 ± 0.63 | >10 | 1.21 ± 0.10 | |
| PC9-GR4 | G | T790M | 29±1 | **>10** | 69 ± 4 |  | **0.12 ± 0.01** | 27 ± 1 |  | **>1** | 53 ± 3 |  | 1.52 ± 0.08 | >10 | 2.80 ± 0.22 | |
| PC9-GR5 | G | FGFR1 ov. | 31±5 | **>10** | 67 ± 4 |  | **0.16 ± 0.04** | 23 ± 3 |  | **>1** | 56 ± 9 |  | 1.67 ± 0.14 | >10 | 1.27 ± 0.06 | |
| PC9-ER | E | Unknown | 27±2 | **0.04 ± 0.02** | 11 ± 2 |  | **0.04 ± 0.04** | 17 ± 1 |  | **0.0036 ± 0.0001** | 12 ± 2 |  | 3.08 ± 0.47 | >10 | 2.99 ± 0.10 | |
| PC9-GR1-AZD1 | O | Unknown | 32±5 | **0.04 ± 0.00** | 21 ± 2 |  | **0.03 ± 0.00** | 18 ± 2 |  | **0.0047 ± 0.0002** | 19 ± 2 |  | 0.95 ± 0.06 | >10 | 1.95 ± 0.29 | |
| PC9-GR1-AZD2 | O | Unknown | 25±4 | **0.03 ± 0.00** | 12 ± 3 |  | **0.02 ± 0.01** | 18 ± 1 |  | **0.0039 ± 0.0003** | 19 ± 0 |  | 2.63 ± 0.15 | >10 | 0.79 ± 0.14 | |
| PC9-GR1-AZD3 | O | Unknown | 26±6 | **0.03 ± 0.01** | 13 ± 0 |  | **0.03 ± 0.00** | 12 ± 0 |  | **0.0040 ± 0.0004** | 20 ± 1 |  | 1.95 ± 0.13 | >10 | 2.76 ± 0.08 | |
| PC9-GR1-AZD4 | O | Unknown | 30±3 | **0.03 ± 0.00** | 11 ± 1 |  | **0.02 ± 0.00** | 16 ± 1 |  | **0.0023 ± 0.0000** | 15 ± 0 |  | 1.29 ± 0.16 | >10 | 2.41 ± 0.05 | |
| PC9-GR4-AZD1 | O | Unknown | 26±4 | **>10** | 50 ± 1 |  | **0.09 ± 0.01** | 23 ± 0 |  | **>1** | 66 ± 3 |  | 1.27 ± 0.23 | 0.31 ± 0.08 | 0.52 ± 0.07 | |
| PC9-GR4-AZD2 | O | FGFR1 ov. | 34±7 | **>10** | 56 ± 3 |  | **0.06 ± 0.01** | 16 ± 1 |  | **>1** | 86 ± 2 |  | 1.63 ± 0.24 | >10 | 0.66 ± 0.11 | |
| 11-18 | P | -- | 23±4 | **>10** | 83 ± 8 |  | **1.16 ± 0.12** | 62 ± 1 |  | **0.445 ± 0.075** | 54 ± 1 |  | 5.26 ± 0.04 | >10 | 0.63 ± 0.31 | |
| 11-18 GR1 | G | *NRAS* Q61 | 27±4 | **>10** | 67 ± 7 |  | **0.72 ± 0.03** | 60 ± 1 |  | **>1** | 50 ± 4 |  | 1.02 ± 0.05 | >10 | 0.48 ± 0.23 | |
| 11-18 GR2 | G | *NRAS* Q61 | 25±2 | **>10** | 74 ± 9 |  | **2.51 ± 0.48** | 78 ± 6 |  | **0.249 ± 0.037** | 45 ± 1 |  | 2.72 ± 0.12 | >10 | 0.55 ± 0.27 | |
| 11-18 GR3 | G | Unknown | 24±2 | **2.14 ± 0.14** | 53 ± 6 |  | **0.62 ± 0.04** | 42 ± 3 |  | **0.260 ± 0.048** | 46 ± 4 |  | 1.03 ± 0.08 | >10 | 0.46 ± 0.01 | |
| 11-18 GR4 | G | *NRAS* Q61 | 22±1 | **>10** | 94 ± 4 |  | **1.02 ± 0.22** | 63 ± 4 |  | **>1** | 54 ± 9 |  | 0.71 ± 0.08 | >10 | 1.00 ± 0.35 | |
| 11-18 GR5 | G | Unknown | 27±4 | **6.09 ± 0.47** | 67 ± 12 |  | **2.54 ± 0.33** | 57 ± 6 |  | **0.211 ± 0.072** | 51 ± 4 |  | 1.22 ± 0.12 | >10 | 0.52 ± 0.01 | |
| 11-18 GR6 | G | *NRAS* Q61 | 22±1 | **>10** | 71 ± 9 |  | **0.95 ± 0.30** | 51 ± 4 |  | **>1** | 54 ± 2 |  | 3.49 ± 0.22 | 4.07 ± 1.84 | 0.44 ± 0.03 |  |

**Table S6 (excel file).** Results of the mRNA expression analysis by nCounter in the PC9 and 11-18 derived clones. The custom panel shown Table S4 was used. Samples were run in triplicates, results shown are average counts normalized to the housekeeping genes. Comparisons between groups of clones were done using a two-tailed Student’s t test.

**Table S7.** Chromosome coordinates of the Chr22q11 amplification in EGFR-TKI resistant clones and NCI-H1819 cells.

| **Cells** | **Start** | **End** | **Start**  **(2nd segment)** | **End**  **(2nd segment)** |
| --- | --- | --- | --- | --- |
| PC9-ER, PC9-GR1AZD1 to 4 | 22:17071767 | 22:22842092 | 22:24698200 | 22:25334116 |
| PC9-GR3 | 22:17662373 | 22:23237555 | 22:26829582 | 22:28559080 |
| PC9-R5 | 22:16590877 | 22:22642881 | NA | NA |
| NCI-H1819 | 22:17071767 | 22:21823906 | NA | NA |
| **COMMON** | **22:17662373** | **22:21823906** |  |  |

**Table S8.** Results of the FISH analysis of Chr22q11 genes in EGFR TKI resistant clones. ND, not determined.

| Cells | *HIRA* copies/cell  (average) | *CRKL*  copies/cell  (average) | *MAPK1* copies/cell  (average) | % Cells  >5 copies | Ratio HIRA:SHANK* |
| --- | --- | --- | --- | --- | --- |
| PC9_(R) | 2 | 2 | 2 | 0% | ND |
| PC9-ER Gefit (S) | 7 | 7 | 7 | 96% | 2.5 |
| PC9-GR1 Gefit_(R) | 2 | 2 | 2 | 0% | ND |
| PC9-GR2 Gefit_(R) | 2 | 2 | 2 | 4% | ND |
| PC9-GR3 Gefit (S) | 8 | 8 | 8 | 100% | 2.9 |
| PC9-GR4 Gefit_(R) | 2 | 2 | 2 | 1% | ND |
| PC9-GR5 Gefit (R) | 2 | 2 | 2 | 0% | ND |
| PC9-GR1AZD1 Osi (S) | 10 | 10 | 10 | 73% | 2.6 |
| PC9-GR1AZD2 Osi (S) | 7 | 7 | 7 | 85% | 2.6 |
| PC9-GR1AZD3 Osi (S) | 7 | 7 | 7 | 88% | 2.6 |
| PC9-GR1AZD4 Osi (S) | 8 | 8 | 8 | 89% | 2.1 |
| PC9-GR4AZD1 Osi_(R) | 2 | 2 | 2 | 0% | ND |
| PC9-GR4AZD2 Osi (R) | 3 | 3 | 3 | 20% | ND |
| 11-18_(R) | 3 | 3 | 3 | 0% | ND |
| 11-18 GR1 Gefit_(R) | 2 | 2 | 2 | 0% | ND |
| 11-18 GR2 Gefit_(R) | 3 | 3 | 3 | 0% | ND |
| 11-18 GR3 Gefit (R) | 3 | 3 | 3 | 3% | ND |
| 11-18 GR4 Gefit_(R) | 3 | 3 | 3 | 0% | ND |
| 11-18 GR5 Gefit_(R) | 3 | 3 | 3 | 1% | ND |
| 11-18 GR6 Gefit (R) | 3 | 3 | 3 | 8% | ND |

*In clones >3 copies of HIRA

**Table S9.** Sensitivity to Aurora inhibitors and Chr22q11 status of EGFR TKI clones used in the study (second panel). The IC50s and percentage of cells surviving at 500 nM inhibitor, in MTT assays, are indicated.

| **Clone** | **Resistant to** | **Origin** | **AZD2811**  **IC50 (μM)** | **AZD2811**  **% Cells** | ***HIRA/CRKL/MAPK* copies per cell (average)** | **% Cells**  **>5 copies** |  |
| --- | --- | --- | --- | --- | --- | --- | --- |
| PC9-R2 | Osimertinib | Cambridge | >10 | 76 ± 3 | 2 | 0% |  |
| PC9-R3 | Osimertinib | Cambridge | >10 | 66 ± 3 | 2 | 0% |  |
| PC9-R5 | Osimertinib | Cambridge | 0.05 ± 0.01 | 28 ± 1 | 6 | 65% |  |
| 11-18 R1 | Osimertinib | Cambridge | >10 | 81 ± 9 | 3 | 0% |  |
| 11-18 R2 | Osimertinib | Cambridge | >10 | 83 ± 5 | 3 | 0% |  |
| HCC827ER10 | Erlotinib | Odense | >10 | 88 ± 6 | 2 | 0% |  |
| HCC827ER20 | Erlotinib | Odense | >10 | 61 ± 1 | 2 | 0% |  |
| HCC827ER30 | Erlotinib | Odense | >10 | 64 ± 6 | 2 | 0% |  |
| PC9-OR1 | Osimertinib | Barcelona | >10 | 50 ± 1 | 2 | 2% |  |
| PC9-OR2 | Osimertinib | Barcelona | >10 | 71 ± 8 | 2 | 0% |  |
| PC9-OR4 | Osimertinib | Barcelona | 0.08 ± 0.03 | 14 ± 1 | 8 | 79% |  |
| PC9-OR5 | Osimertinib | Barcelona | >10 | 51 ± 2 | 3 | 9% |  |

**Table S10 (excel file).** mRNAs and proteins significantly up and downregulated in PC9-ER and PC9-GR1 to GR5 clones by WTS and proteomics, respectively. Parental PC9 was used as a reference. RNAs corresponding to genes located in Chr22q11 are highlighted in yellow.

**Table S11.** Genes targeted by the knock-out library used in the study, reason(s) to be selected and essentiality scores.

|  | | **PC-9 GR3 arrayed screen** | | **DepMap data** | |
| --- | --- | --- | --- | --- | --- |
| **Gene Symbol** | **Reason for selection** | **Essentiality in PC9-GR3 from arrayed screen** | **Fitness to GR3** | **Essentiality in DepMap** | **Essential in no of cell lines** |
| *ADA2* | located in 22q11 | Not essential | 1.294453665 | Not essential | 0/1107 |
| *ADORA2A* | located in 22q11 | Not essential | 0.590185105 | Strongly selective | 12/1107 |
| *AIFM3* | located in 22q11 | Not essential | 1.147008179 | Strongly selective | 8/1107 |
| *ARVCF* | located in 22q11 | Not essential | 0.98407232 | Not essential | 0/1107 |
| *AURKB* | Cell cycle. Target of AZD2811 | Essential | -0.119945 | Common essential | 1106/1107 |
| *BCL2* | Cell cycle | Not essential | 1.00796384 | Strongly selective | 39/1107 |
| *BCL2L13* | located in 22q11 | Not essential | 0.933706414 | Strongly selective | 6/1107 |
| *BCR* | located in 22q11 | Not essential | 1.058544985 | Strongly selective | 8/1107 |
| *BID* | located in 22q11 | Not essential | 1.187688334 | Strongly selective | 10/1107 |
| *CABIN1* | located in 22q11 | Not essential | 0.780456307 | Strongly selective | 30/1107 |
| *CASP2* | PIDDosome complex | Not essential | 0.861386139 | Not essential | 0/1107 |
| *CCDC116* | located in 22q11 | Not essential | 1.103314679 | Strongly selective | 3/1107 |
| *CDC45* | located in 22q11 | Essential | -0.09298 | Common essential | 1107/1107 |
| *CDK6* | Cell cycle | Not essential | 1.076625054 | Strongly selective | 555/1107 |
| *CHCHD10* | located in 22q11 | Not essential | 0.856220405 | Strongly selective | 24/1107 |
| *CLDN5* | located in 22q11 | Not essential | 1.030563926 | Not essential | 0/1107 |
| *CLTCL1* | located in 22q11 | Not essential | 1.043126685 | Strongly selective | 73/1107 |
| *COMT* | located in 22q11 | Not essential | 1.24795523 | Strongly selective | 44/1107 |
| *CRADD* | PIDDosome complex | Not essential | 1.068733154 | Strongly selective | 1/1107 |
| *CRKL* | located in 22q11 | Not essential | 0.779164873 | Strongly selective/common essential | 793/1107 |
| *DGCR8* | located in 22q11 | Essential | 0.064690027 | Common essential | 830/1107 |
| *EGFR* | RTK. Target of osimertinib | Not essential (Fitness effected) | 0.402927249 | Strongly selective | 293/1107 |
| *ESS2* | located in 22q11 | Possibly essential | 0.012914335 | Common essential | 1105/1107 |
| *GAB4* | located in 22q11 | Essential | 0.072750753 | Not essential | 0/1107 |
| *GGT1* | located in 22q11 | Not essential | 1.005165734 | Common essential | 921/1107 |
| *GGT2* | located in 22q11 | Not essential | 0.952647439 | n/a |  |
| *GGTLC2* | located in 22q11 | Essential | -0.027550581 | Common Essential | 1102/1107 |
| *GGTLC3* | located in 22q11 | Possibly essential | 0.051226862 | n/a |  |
| *GNB1L* | located in 22q11 | Essential | -0.332328885 | Common essential | 932/1107 |
| *GRK3* | located in 22q11 | Not essential | 0.848041326 | Strongly selective | 2/1107 |
| *GSC2* | located in 22q11 | Not essential | 0.967654987 | Strongly selective | 1/1107 |
| *HDHD5* | located in 22q11 | Not essential | 0.693930263 | Strongly selective | 21/1107 |
| *HIC2* | located in 22q11 | Not essential | 1.077055532 | Strongly selective | 22/1107 |
| *HIRA* | located in 22q11 | Essential | -0.010761946 | Common Essential | 817/1107 |
| *HPS4* | located in 22q11 | Not essential | 1.11708997 | Not essential | 0/1107 |
| *IL17RA* | located in 22q11 | Not essential | 1.147653896 | Not essential | 0/1107 |
| *KLHL22* | located in 22q11 | Not essential | 0.957382695 | Strongly selective | 5/1107 |
| *LZTR1* | located in 22q11 | Not essential | 0.848471804 | Strongly selective | 12/1107 |
| *MAPK1* | located in 22q11 | Not essential | 0.851054671 | Strongly selective | 144/1107 |
| *MED15* | located in 22q11 | Not essential | 1.037021093 | Strongly selective | 191/1107 |
| *MICAL3* | located in 22q11 | Not essential | 0.66078347 | Strongly selective | 3/1107 |
| *MIF* | located in 22q11 | Not essential | 0.83727938 | Strongly selective | 2/1107 |
| *MRPL40* | located in 22q11 | Not essential | 0.725785622 | Not essential | 0/1107 |
| *NNP11* | located in 22q11 | Essential | -0.0606469 | Strongly selective | 221/1107 |
| *P2RX6* | located in 22q11 | Not essential | 1.059836418 | Strongly selective | 6/1107 |
| *PI4KA* | located in 22q11 | Essential | -0.041778976 | Common Essential | 1061/1107 |
| *PIDD1* | PIDDosome complex | Not essential | 1.021954369 | Strongly selective | 2/1107 |
| *PITPNB* | located in 22q11 | Not essential | 1.058114507 | Strongly selective | 24/1107 |
| *PPIL2* | located in 22q11 | Not essential | 0.920361601 | Common Essential | 1097/1107 |
| *PPM1F* | located in 22q11 | Not essential | 0.657681941 | Strongly selective | 5/1107 |
| *PRAME* | located in 22q11 | Not essential | 1.163151098 | Not essential | 0/1107 |
| *PRODH* | located in 22q11 | Not essential | 0.556607835 | Strongly selective | 10/1107 |
| *RANBP1* | located in 22q11 | Essential | -0.067154542 | Common Essential | 711/1107 |
| *RB1* | Cell cycle | Not essential | 1.088678433 | Not essential | 0/1107 |
| *RGL4* | located in 22q11 | Not essential | 0.932414981 | Strongly selective | 127/1107 |
| *RTN4R* | located in 22q11 | Not essential | 0.946620749 | Strongly selective | 3/1107 |
| *SCARF2* | located in 22q11 | Not essential | 0.817046922 | Strongly selective | 22/1107 |
| *SDF2L1* | located in 22q11 | Not essential | 1.07145932 | Strongly selective | 47/1107 |
| *SEPTIN5* | located in 22q11 | Not essential | 0.964700818 | Strongly selective | 8/1107 |
| *SLC25A1* | located in 22q11 | Not essential | 0.851752022 | Strongly selective | 131/1107 |
| *SLC7A4* | located in 22q11 | Not essential | 1.017219113 | Strongly selective | 7/1107 |
| *SMARCB1* | located in 22q11 | Essential | -0.053908356 | Common Essential | 927/1107 |
| *SPECC1L* | located in 22q11 | Fitness effected | 0.476969436 | Strongly selective | 59/1107 |
| *TANGO2* | located in 22q11 | Not essential | 1.06543263 | Not essential | 0/1107 |
| *TBX1* | located in 22q11 | Fitness effected | 0.464916057 | Strongly selective | 11/1107 |
| *THAP7* | located in 22q11 | Not essential | 0.939302626 | Strongly selective | 32/1107 |
| *TOP3B* | located in 22q11 | Fitness effected | 0.412397762 | Strongly selective | 16/1107 |
| *TRMT2A* | located in 22q11 | Not essential | 1.021563342 | Strongly selective | 14/1107 |
| *TSSK2* | located in 22q11 | Not essential | 0.985794232 | Strongly selective | 2/1107 |
| *TXNRD2* | located in 22q11 | Not essential | 1.007318123 | Strongly selective | 8/1107 |
| *UBE2L3* | located in 22q11 | Essential | -0.032716315 | Common Essential | 1090/1107 |
| *UFD1* | located in 22q11 | Essential | -0.060266896 | Common Essential | 1107/1107 |
| *USP18* | located in 22q11 | Fitness effected | 0.401635816 | Strongly selective | 364/1107 |
| *USP41* | located in 22q11 | Not essential | 0.845458459 | Data not available |  |
| *VPREB3* | located in 22q11 | Not essential | 1.082221266 | Not essential | 0/1107 |
| *YPEL1* | located in 22q11 | Fitness effected | 0.494188549 | Strongly essential/Common Essential | 460/1107 |
| *ZDHHC8* | located in 22q11 | Not essential | 0.904864399 | Strongly selective | 37/1107 |
| *ZNF280A* | located in 22q11 | Not essential | 0.834266035 | Strongly selective | 2/1107 |
| *ZNF280B* | located in 22q11 | Not essential | 0.98019802 | Not essential | 0/1107 |
| *ZNF74* | located in 22q11 | Not essential | 0.925096858 | Strongly selective | 6/1107 |

**Table S12.** Sensitivity to AURKB, AURKA and TTK inhibitors of tumor cell lines of different origins. The IC50s and the % of survival in MTT assays at 500 nM (for AZD2811 and LY3295669) or 50 nM inhibitor (for BAY1217389) are indicated. GB, glioblastoma; HNSCC, head and neck squamous cell carcinoma; LA, lung adenocarcinoma; LS, lung squamous cell carcinoma; Pr, prostate; UB, urinary bladder.

| **Cell line** | **Origin** | **Td (h)** | **AZD2811 (AURKBi)** | |  | **LY3295668 (AURKAi)** | |  | | **BAY1217389 (TTKi)** | | | |  |
| --- | --- | --- | --- | --- | --- | --- | --- | --- | --- | --- | --- | --- | --- | --- |
|  |  |  | **IC50(µM)** | **%** |  | **IC50(µM)** | **%** |  | | **IC50(µM)** | | **%** | |  |
| A549 | LA | 22 | **>10** | 79 ± 2 |  | **0.52 ± 0.07** | 50 ± 1 |  | **>1** | | 55 ± 5 | |  | |
| DU-145 | Pr | 30 | **>10** | 64 ± 3 |  | **0.60 ± 0.12** | 51 ± 3 |  | **>1** | | 54 ± 5 | |  | |
| EBC-1 | LS | 42 | **>10** | 69 ± 2 |  | **0.09 ± 0.02** | 39 ± 1 |  | **0.0046 ± 0.0006** | | 29 ± 1 | |  | |
| FADU | HNSCC | 30 | **>10** | 61 ± 7 |  | **>10** | 68 ± 3 |  | **>1** | | 73 ± 4 | |  | |
| HCC366 | LA | 65 | **>10** | 89 ± 12 |  | **0.22 ± 0.02** | 34 ± 2 |  | **>1** | | 84 ± 3 | |  | |
| HCC78 | LA | 38 | **>10** | 62 ± 0 |  | **>10** | 74 ± 3 |  | **>1** | | 52 ± 2 | |  | |
| LC-2/ad | LA | 58 | **0.05 ± 0.01** | 10 ± 1 |  | **0.83 ± 0.20** | 54 ± 2 |  | **0.0036 ± 0.0003** | | 18 ± 1 | |  | |
| MDA-MB-231 | Breast | 25 | **>10** | 74 ± 6 |  | **>10** | 84 ± 1 |  | **>1** | | 53 ± 2 | |  | |
| MDA-MB-468 | Breast | 35 | **0.03 ± 0.01** | 16 ± 2 |  | **0.05 ± 0.01** | 9 ± 0 |  | **0.0041 ± 0.0003** | | 9 ± 1 | |  | |
| MIA-PaCa-2 | Pancreas | 26 | **>10** | 83 ± 3 |  | **>10** | 62 ± 8 |  | **>1** | | 56 ± 5 | |  | |
| NCI-H1819 | LA | 51 | **0.01 ± 0.00** | 17 ± 6 |  | **0.45 ± 0.08** | 41 ± 2 |  | **0.0026 ± 0.0003** | | 16 ± 0 | |  | |
| NCI-H1975 | LA | 39 | **>10** | 88 ± 0 |  | **0.40 ± 0.06** | 44 ± 0 |  | **>1** | | 54 ± 4 | |  | |
| NCI-H2228 | LA | 96 | **0.30 ± 0.11** | 40 ± 5 |  | **0.07 ± 0.01** | 17 ± 1 |  | **>1** | | 69 ± 0 | |  | |
| NCI-H23 | LA | 38 | **>10** | 71 ± 8 |  | **0.09 ± 0.01** | 38 ± 1 |  | **>1** | | 64 ± 6 | |  | |
| NCI-H3122 | LA | 49 | **0.07 ± 0.01** | 22 ± 2 |  | **0.40 ± 0.08** | 37 ± 1 |  | **0.0072 ± 0.0005** | | 21 ± 2 | |  | |
| RT-112 | UB | 24 | **0.31 ± 0.04** | 34 ± 1 |  | **1.57 ± 0.03** | 75 ± 2 |  | **0.0038 ± 0.0003** | | 22 ± 0 | |  | |
| SK-MES-1 | LS | 50 | **>10** | 73 ± 0 |  | **0.10 ± 0.01** | 24 ± 1 |  | **>1** | | 59 ± 9 | |  | |
| SK-OV-3 | Ovarian | 48 | **>10** | 93 ± 4 |  | **>10** | 59 ± 4 |  | **>1** | | 64 ± 4 | |  | |
| SNU-C1 | Colon | 31 | **>10** | 73 ± 5 |  | **>10** | 72 ± 4 |  | **>1** | | 67 ± 5 | |  | |
| T98G | GB | 22 | **>10** | 68 ± 3 |  | **>10** | 61 ± 4 |  | **>1** | | 63 ± 4 | |  | |
| WM793 | Melan. | 48 | **>10** | 68 ± 2 |  | **>10** | 46 ± 0 |  | **>1** | | 68 ± 9 | |  | |

**Table S13.** BID levels, genotype for selected genes and sensitivity to AURKB, TTK and AURKA inhibitors of cell lines and clones used in this study. *TP53*, *Rb1*, *MYC* and *CTNNB1* were sequenced by NGS (see Supplementary Methods) and the results were coincident with the genotypes reported in the COSMIC and Cellosaurus databases. High BID mRNA levels (2^-∆Ct^x100 ≥0.5) are indicated in grey. P, proficient; N, null. R, resistant; S, sensitive. In the two *BID* CNG-positive cell lines, the *BID/MAPK1* ratio is indicated.

**Table S14.** Tumor samples included in the studies of Chr22q11 copy number by Q-PCR and *BID* mRNA levels by RT-Q-PCR.

|  | **Chr22q11 study** | | ***BID* mRNA study (prospective)** | |
| --- | --- | --- | --- | --- |
| **Characteristics** | **N = 143** | **(%)** | **N = 96** | **(%)** |
| **Type of tumor** |  |  |  |  |
| Bladder | 8 | 5.6 | 6 | 6.3 |
| Breast | 16 | 11.2 | 1 | 1.0 |
| Colon | 22 | 15.4 | 10 | 10.4 |
| Lung | 55 | 38.5 | 61 | 63.5 |
| Melanoma | 19 | 13.3 | 2 | 2.1 |
| Ovarian | 8 | 5.6 | 5 | 5.2 |
| Others | 15 | 10.5 | 11 | 11.5 |
| **Collection time** |  |  |  |  |
| No data | **--** | **--** | 6 | 6.3 |
| Basal | 104 | 72.7 | 85 | 88.5 |
| Progression | 39 | 27.3 | 5 | 5.2 |
| *EGFR TKIs* | *20* | *14.0* | *1* | *1.0* |
| *ICIs ± chemotherapy* | *14* | *9.8* | *2* | *2.1* |
| *Chemotherapy* | *5* | *3.5* | *2* | *2.1* |

**Table S15**. Characteristics of the six FFPE tumor samples with high levels of *BID* mRNA found in the prevalence study (see Fig. 8D). All samples were wt for *EGFR*.

| **Sample ID** | **BID mRNA** | **Tumor** | **Histology** | **Collection time** | **Genotype (NGS)** |
| --- | --- | --- | --- | --- | --- |
| P91 | 0.35 | Lung, Stage IV | Adenocarcinoma | Presentation | *TP53*mut (p.P278S) |
| P92 | 0.36 | Lung, extensive stage | Small cell | Progression to cisplatin | *PIK3CA*mut (p.E542K) |
| P93 | 0.43 | Lung, Stage IV | Squamous carcinoma | Presentation | *TP53*mut (p.Q165*) |
| P94 | 0.46 | Lung, Stage IV | Adenocarcinoma | Presentation | wt |
| P95 | 0.49 | Colon, Stage IV | Adenocarcinoma | Presentation | *TP53*mut (p.R175H),  *PIK3CA*mut (p.H1047R) |
| P96 | 0.66 | Liposarcoma | ND | Presentation | *PIK3CA*mut (p.H1047R) |
